# Liquid‐Exfoliated 2D Materials for Optoelectronic Applications

**DOI:** 10.1002/advs.202003864

**Published:** 2021-03-11

**Authors:** Fuad Indra Alzakia, Swee Ching Tan

**Affiliations:** ^1^ Department of Materials Science and Engineering National University of Singapore 9 Engineering drive 1 Singapore 117574 Singapore

**Keywords:** 2D materials, liquid exfoliation, optoelectronic devices, photodetector

## Abstract

Two‐dimensional (2D) materials have attracted tremendous research attention in recent days due to their extraordinary and unique properties upon exfoliation from the bulk form, which are useful for many applications such as electronics, optoelectronics, catalysis, etc. Liquid exfoliation method of 2D materials offers a facile and low‐cost route to produce large quantities of mono‐ and few‐layer 2D nanosheets in a commercially viable way. Optoelectronic devices such as photodetectors fabricated from percolating networks of liquid‐exfoliated 2D materials offer advantages compared to conventional devices, including low cost, less complicated process, and higher flexibility, making them more suitable for the next generation wearable devices. This review summarizes the recent progress on metal–semiconductor–metal (MSM) photodetectors fabricated from percolating network of 2D nanosheets obtained from liquid exfoliation methods. In addition, hybrids and mixtures with other photosensitive materials, such as quantum dots, nanowires, nanorods, etc. are also discussed. First, the various methods of liquid exfoliation of 2D materials, size selection methods, and photodetection mechanisms that are responsible for light detection in networks of 2D nanosheets are briefly reviewed. At the end, some potential strategies to further improve the performance the devices are proposed.

## Graphical abstract

This review summarizes the recent progress on metal–semiconductor–metal photodetectors fabricated from percolating network of 2D materials nanosheets obtained from liquid exfoliation methods, including hybrids with other nanomaterials and polymers. This novel route enables a more facile and low‐cost route to produce optoelectronic devices which are suitable for the next generation of flexible and wearable devices.

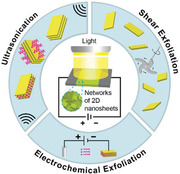

## 1 Introduction

Photodetector is a kind of optoelectronic device that converts the incoming light signal into an electrical signal, where semiconductor is the predominant type of material where electrical signals are generated by way of photoelectric effect. The emergence and development of photodetectors have allowed various modern applications such as optical communications, biomedical imaging, video imaging, security, motion detection, and remote sensing.^[^ ^1^, ^2^ ^]^ The applications of photodetectors can be broadly categorized into two categories: communications, where the light signal is the carrier of the encoded signal, and remote sensing, where the light signal conveys information about the object under observation. In communications applications, photodetectors mainly operate in the near‐infrared (IR) region of the spectrum and need to have extremely fast response and high reliability, but high sensitivity is not the priority. Photodiodes based on indium gallium arsenide (InGaAs) have been successfully used for optical communications, reaching rates as high as 2.5 Gbit s^−1^.^[^ ^3^ ^]^ For remote sensing, metal–semiconductor–metal (MSM) photoconductor type photodetectors are typically used, because they are the cheapest and most rugged type among other detector designs. Examples of applications include home security systems where IR‐sensitive motion detectors are used, and factory safety detectors, where visible or ultraviolet (UV) sensitive detectors are used.

Modern commercial photodetectors are mainly made from crystalline nonlayered inorganic semiconductors such as silicon or III–IV compounds.^[^ ^4^, ^5^ ^]^ Unfortunately, due to their intrinsic bandgap of 1.1 eV, silicon photodetectors have limited spectral response range only from 300 to 1100 nm, limiting their range of applications. Besides, the fabrication cost of silicon photodetectors based on vapor phase processing is very high, due to the use of expensive equipments, complicated processing steps, and huge energy consumption. Similarly, complicated fabrication process is also required for photodetectors based on gallium arsenide (GaAs) and InGaAs. Even though they have higher electron mobility and lower power consumption during operation compared to silicon photodetectors, limited reserves of raw materials on earth and the high toxicity of the arsenic (As) element greatly limits the widespread application of As‐based photodetectors. More importantly, the rigid and mechanically inflexible nature of crystalline silicon, GaAs, and InGaAs semiconductors prevents them to be used in next generation, flexible wearable electronics. Therefore, novel photoactive semiconductor materials that overcome the above limitations need to be investigated.

Solution‐processed semiconductors are emerging class of photoactive materials which is more favorable than vapor phase processing methods because it allows great reduction in energy consumption and obviate the need for expensive experimental instruments and procedures.^[^ ^6^, ^7^ ^]^ Moreover, they are more compatible with large‐area device fabrication on flexible substrate for wearable devices, such as polyethylene terephthalate (PET),^[^ ^8^, ^9^ ^]^ polydimethylsiloxane (PDMS),^[^ ^10^, ^11^ ^]^ polyethylene naphthalate (PEN),^[^ ^12^, ^13^ ^]^ and papers.^[^ ^14^, ^15^ ^]^ Photoactive materials for solution‐processing of photodetectors include inorganic materials such as quantum dots,^[^ ^16^, ^17^ ^]^ nanorods,^[^ ^18^, ^19^, ^20^ ^]^ nanowires,^[^ ^21^, ^22^, ^23^ ^]^ nanoplatelets,^[^ ^24^, ^25^, ^26^ ^]^ and 2D materials,^[^ ^27^, ^28^, ^29^ ^]^ and organic materials, such as organic molecules^[^ ^30^, ^31^, ^32^ ^]^ and polymers.^[^ ^33^, ^34^, ^35^ ^]^ Solution‐processes photoactive materials have great potentials for the next‐generation photodetecting applications.

Layered 2D materials have received tremendous research attention since the discovery of graphene due to their unique and distinctive properties. They offer plenty of opportunities for the next generation of electronic and optoelectronic applications and technologies.^[^ ^36^ ^]^ To date, other than graphene, significant number of 2D materials have been reported, such as transition metal dichalcogenides (TMDs),^[^ ^37^, ^38^ ^]^ phosphorene,^[^ ^39^, ^40^ ^]^ antimonene,^[^ ^41^, ^42^ ^]^ topological insulators,^[^ ^43^, ^44^ ^]^ MXenes,^[^ ^45^, ^46^ ^]^ etc. These materials have been shown to possess properties that are advantageous for the next generation optoelectronic applications, such as tunable band structures,^[^ ^47^, ^48^ ^]^ high carrier mobility,^[^ ^49^, ^50^ ^]^ layer dependent optical properties,^[^ ^51^, ^52^ ^]^ and high light absorption coefficient.^[^ ^53^ ^]^ Exfoliation of these bulk layered 2D materials into single‐ or few‐layer 2D nanosheets opens up wide range of novel interesting properties that are useful in many applications.^[^ ^54^, ^55^ ^]^ To date, production of layered 2D materials can be categorized into two major methods: bottom–up methods and top–down methods. Bottom–up synthesis of 2D materials include chemical vapor deposition (CVD), epitaxial growth, and hydrothermal methods. CVD method has been one of the most important methods for the synthesis of 2D materials, where precursors are involved in an activated chemical reaction in a specially controlled environment. The precursors, the atmosphere, temperature, substrate, and catalysts are some of the key factors determining the quality of the synthesized 2D materials. However, CVD methods require harsh growth conditions (high temperature and high vacuum), and the size of the 2D materials can be limited. Therefore, CVD methods are not suitable for low‐cost and mass production of 2D materials. In hydrothermal methods, 2D materials are synthesized from heterogeneous reactions in aqueous media by applying high temperature and pressure. The aqueous mixture of precursors sealed in stainless steel autoclave and heated above the boiling point of water, thus dramatically increasing the pressure. However, this method requires expensive autoclaves and also has difficulty in observing the crystal growth process during synthesis.

On the other hand, top–down synthesis of 2D materials include micromechanical exfoliation^[^ ^56^, ^57^ ^]^ and liquid‐based exfoliation methods.^[^ ^58^, ^59^ ^]^ Because layered 2D materials are made of strong in‐plane chemical bonds but weak inter‐layer bonds (van der Waals bonds), exfoliating the bulk 2D material down to monolayer limit is possible. Mechanical exfoliation involves the use of mechanical forces to separate bulk 2D materials into mono‐ or few‐layer nanosheets. This process is usually done by using an adhesive tape to attach the surfaces of the 2D material and use force to peel off the tapes alongside the layers of the 2D materials, or by rubbing the surface of the 2D materials against another material to shear of the layers from the bulk material. However, this method only produce small quantity of exfoliated nanosheets, thus only suitable for fundamental studies.

In liquid‐based exfoliation methods, the bulk layered 2D materials are exfoliated in liquid media into mono‐ or few‐layer 2D nanosheets via a variety of methods.^[^ ^59^ ^]^ They consist of a collection of different methods, such as ultrasonication, electrochemical exfoliation, and shear exfoliation. These methods have shown remarkable progress in producing a wide variety (virtually any) 2D nanosheets in large quantity in a low‐cost and environmentally friendly manner, thus more and more research attention has been placed on these methods recently. The exfoliated 2D nanosheets can then be sorted based on size and thickness to produce uniform dispersions. Among liquid‐based exfoliation methods, some techniques apply chemical reactions to the bulk 2D powder to make it more soluble to the liquid medium being used. This is particularly the case for synthesis of graphene oxide (GO) from bulk graphite powder via chemical oxidation,^[^ ^60^, ^61^ ^]^ exfoliation of TMD layered materials by intercalation of lithium ions which results in phase transformation from semiconducting 2H to metallic 1T phase,^[^ ^62^ ^]^ and selective etching of an A‐group element of layered M_n+1_AX_n_ (MAX) phases to obtain single‐layer MXene nanosheets.^[^ ^45^, ^63^ ^]^ Meanwhile in direct liquid exfoliation methods, the bulk 2D materials are directly exfoliated in liquid media without the need for chemical reactions. These methods generally result in a high‐quality, pristine 2D nanosheets with minimal amount of defects, as long as chemical reactions are nonexistent between the solvent and the 2D materials during the exfoliation process.^[^ ^38^, ^64^ ^]^ The exfoliated 2D nanosheets obtained will have desirable material properties and are highly dispersible in the solvent, making them convenient to be mixed with other nanomaterials solutions to form composites and hybrids,^[^ ^65^ ^]^ and can be casted onto any substrate in the form of thin film for device and coating applications.

**Scheme 1:**
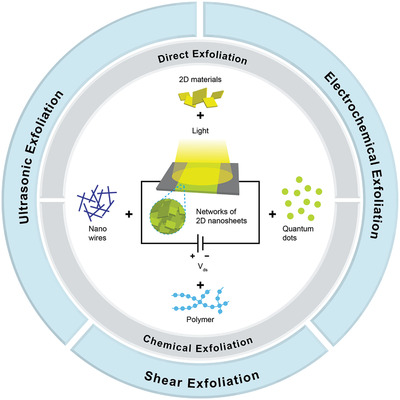
Schematic of metal–semiconductor–metal photodetector made of percolating network of liquid‐exfoliated 2D nanosheets.

In view of the ongoing rapid progress and the great advantages the liquid exfoliation methods bring for the next generation of high‐performance wearable optoelectronic devices, in this article we aim to present a comprehensive and up‐to‐date review of photodetector applications where the active channel materials are obtained from percolating networks of liquid‐exfoliated 2D materials, which include graphene, black phosphorus, antimonene, graphitic carbon nitride (g‐C_3_N_4_), transition metal dichalcogenides (TMDs), layered III–VI semiconductors, topological insulators, and MXenes. We also discuss hybrid and composite photodetectors which combine the liquid‐exfoliated 2D materials with variety of other nanomaterials such as quantum dots, nanowires (NW), nanorods (NR), other 2D materials, etc. All of the materials used in the photodetectors we present in this article are obtained from liquid exfoliation method, or at least liquid exfoliation was involved during the synthesis process. In the discussion of composite and hybrid photodetectors, at least one component of the photoactive materials is obtained from liquid‐exfoliated 2D materials, while the other hybrid material can be synthesized from other methods, such as sol–gel, hydrothermal, solvothermal, or CVD. Regarding the photodetector design, this review article is focused on the MSM photodetector design (**Scheme** **1**), since this is the cheapest, simplest, and the most rugged type among other photodetector configurations. Therefore, we do not discuss other photodetector configurations such as photodiodes and photoelectrochemical detectors, which also have been fabricated from liquid‐exfoliated 2D nanosheets.^[^ ^66^, ^67^, ^68^, ^69^, ^70^ ^]^ In **Table** **1** we summarize the photodetectors made from liquid‐exfoliated 2D nanosheet that we present in this article. After the brief introduction section, we address various methods of liquid exfoliation, followed by size and thickness selection to obtain the desired size/thickness distribution in the dispersion, followed by discussion of various photodetecting mechanisms and performance parameters, and then we summarize recent developments of photodetectors made of liquid‐exfoliated 2D materials. In the last section of this article, we compare the performances of the photodetectors throughout different wavelength ranges and propose some strategies for further improving the performances of photodetectors.

**Table 1:** Summary of photodetectors obtained from liquid‐exfoliated 2D materials

2D materials		Liquid exfoliation method		Hybrid materials	Responsivity [A W^−1^]	Detectivity [Jones]	References
Graphene	Reduced graphene oxide (rGO)	Chemical exfoliation	Ultrasonication	None	2.5 × 10^−3^ − 1.8 × 10^3^ (UV), 3.5 × 10^−5^ − 7.3 × 10^−1^ (Vis), 4 × 10^−5^ − 7.33 × 10^−1^ (NIR), 4 × 10^−3^ − 7.1 × 10^−1^ (SWIR), 2.52 × 10^−4^ (LWIR), 7.25 × 10^−4^ − 9.87 × 10^−2^ (FIR)	N/A	^[^ ^71^, ^72^, ^73^, ^74^, ^75^, ^76^, ^77^, ^78^, ^79^, ^80^, ^81^, ^82^, ^83^, ^84^, ^85^ ^]^
				Metal quantum dots (Au, Ag, Pt)	9.5 × 10^−3^ (UV), 4 × 10^−3^ (Vis)	N/A	^[^ ^86^, ^87^, ^88^ ^]^
				Semiconductor nanoparticles (ZnO, ZnS, TiO_2_, CdSe, PbS, Cu_2_S, InP, Bi_2_S_3_, WO_3_, HfS_3_, HfSe_3_, Cu_2_SnS_3_, CdZnS_)_	2.13 × 10^−7^ − 1.01 × 10^4^ (UV), 12.13 × 10^−7^ − 2 × 10^3^ (Vis), 1.8 × 10^−7^ − 1.3 × 10^−1^ (NIR),	2.83 × 10^[^ ^10^ ^]^ − 3 × 10^13^ (UV), 5.7 × 10^12^ (NIR),	^[^ ^89^, ^90^, ^91^, ^92^, ^93^, ^94^, ^95^, ^96^, ^97^, ^98^, ^99^, ^100^, ^101^, ^102^, ^103^, ^104^, ^105^, ^106^, ^107^, ^108^, ^109^, ^110^, ^111^, ^112^, ^113^, ^114^, ^115^, ^116^, ^117^ ^]^
				Graphene quantum dots	3 × 10^−6^ − 8.7 × 10^2^ (UV)	7.7 × 10^[^ ^13^ ^]^ (UV)	^[^ ^118^, ^119^ ^]^
				Carbon nanoparticles	0.4 (NIR)	N/A	^[^ ^120^ ^]^
				Perovskite (CH_3_NH_3_PbX_3_ (X = Cl, Br, I), CsPbX_3_ (X = Cl, Br, I))	7.39 × 10^−2^ (Vis)	N/A	^[^ ^121^, ^122^, ^123^ ^]^
				Other 2D materials (SnSe, MoS_2_)	3.21 × 10^−3^ − 4.06 × 10^−2^ (UV), 7.2 × 10^−3^ − 0.115 (Vis)	N/A	^[^ ^124^, ^125^ ^]^
				Organic materials (copper phthalocyanine (TSCuPc), TDPP, TTDPP, P3TOPS, P3TOPA, P(VDF‐TrFE))	1.7 × 10^−5^ − 34.2 (white)	N/A	^[^ ^126^, ^127^, ^128^, ^129^ ^]^
Graphene	Graphene quantum dot (GQD)	Chemical exfoliation (oxidation)	Ultrasonication + hydrothermal	None	2.1 × 10^−3^ − 307 (UV), 0.103 (Vis)	9.59 × 10^[^ ^11^ ^]^ − 1.5 × 10^14^ (UV), 1.5 × 10^10^ (Vis)	^[^ ^130^, ^131^, ^132^ ^]^
				Metal quantum dots (Au)	1.36 (Vis)	2.03 × 10^[^ ^11^ ^]^ (Vis)	^[^ ^133^ ^]^
				Semiconductor nanoparticles (ZnO)	5.5 (UV)	N/A	^[^ ^134^, ^135^ ^]^
	Direct exfoliation	Ultrasonication	Few‐layer graphene (FLG)	None	N/A	N/A	^[^ ^136^ ^]^
				Semiconductor nanoparticles (IGZO, PbSe, TiO_2_)	N/A	N/A	^[^ ^137^, ^138^, ^139^ ^]^
				Other 2D materials (MoS_2_)	3.3 × 10^−3^ (Vis), 3.3 × 10^−3^ (IR),	N/A	^[^ ^140^ ^]^
				Organic materials (azobenzene, PEDOT:PSS)	N/A	N/A	^[^ ^141^, ^142^ ^]^
	Partly reduced graphene aerogel (PRGA)	Chemical exfoliation	Ultrasonication + freeze drying	None	N/A	N/A	^[^ ^143^ ^]^
	Graphene foam	Chemical exfoliation	Ultrasonication + solvothermal + freeze drying	None	1.81 × 10^−4^ (Vis), 7.8 × 10^−5^ − 8.33 × 10^−5^ (NIR), 7.1 × 10^−5^ − 8.33 × 10^−5^ (LWIR), 5 × 10^−5^ (FIR), 2.6 × 10^−5^ (microwave)		^[^ ^144^ ^]^
Black phosphorus (BP)		Direct exfoliation	Ultrasonication	None	N/A	N/A	^[^ ^145^ ^]^
				CVD Graphene	7.7 × 10^[^ ^3^ ^]^ (UV), 2 × 10^3^ − 5 × 10^3^ (Vis), 21 (NIR)	N/A	^[^ ^146^ ^]^
				CVD WS_2_	1.3 × 10^−3^ − 1.2 × 10^−1^ (Vis), 4 × 10^−7^ − 7 × 10^−7^ (NIR)	N/A	^[^ ^147^ ^]^
Antimonene		Direct exfoliation	Ultrasonication	CdS quantum dots	1 × 10^−5^ (white)	N/A	^[^ ^148^ ^]^
Graphitic carbon nitride (g‐C_3_N_4_)		Direct exfoliation	Ultrasonication	Metal halide perovskite	14 (white), 2.4 (Vis)	7.4 × 10^[^ ^12^ ^]^ (Vis)	^[^ ^149^ ^]^
Transition metal dichalcogenides (TMD)	MoS_2_	Direct exfoliation	Ultrasonication	None	5 × 10^−5^ − 5 × 10^−2^ (Vis), 1.9 × 10^−4^ (NIR)	4.31 × 10^[^ ^7^ ^]^ − 3.18 × 10^9^ (Vis)	^[^ ^150^, ^151^, ^152^, ^153^, ^154^, ^155^, ^156^, ^157^ ^]^
		Chemical exfoliation (lithium intercalation)	Ultrasonication	None	4.78 × 10^−4^ (NIR), 3.58 × 10^−4^ (SWIR)	1.1 × 10^[^ ^9^ ^]^ (NIR)	^[^ ^158^ ^]^
		Chemical exfoliation (Pb intercalation)	Ultrasonication	PbS quantum dots	0.543 (NIR)	2 × 10^[^ ^12^ ^]^ (Vis), 2.68 × 10^12^ (NIR)	^[^ ^159^ ^]^
		Direct exfoliation	Ultrasonication	PbSe quantum dots	1.9 × 10^−6^ (>1200 nm)	N/A	^[^ ^160^ ^]^
Transition metal dichalcogenides (TMD)	MoS_2_	Direct exfoliation	Ultrasonication	g‐C_3_N_4_ nanosheets	4 (UV), 7 × 10^−1^ (Vis)	4 × 10^[^ ^11^ ^]^ (UV), 8 × 10^10^ (Vis)	^[^ ^161^ ^]^
		Direct exfoliation	Ultrasonication	Polymer	2.2 × 10^−2^ (Vis)	N/A	^[^ ^162^ ^]^
	MoSe_2_	Direct exfoliation	Ultrasonication	None	4 × 10^−6^ (white)	N/A	^[^ ^156^, ^157^ ^]^
		Direct exfoliation	Ultrasonication	Polymer	16 (NIR)	4 × 10^[^ ^12^ ^]^ (NIR)	^[^ ^162^ ^]^
	MoTe_2_	Direct exfoliation	Ultrasonication	None	6.8 × 10^−5^ (white)	N/A	^[^ ^156^, ^157^ ^]^
		Direct exfoliation	Ultrasonication	None	8 × 10^−5^ (UV), 1 × 10^−3^ − 8.6 × 10^−1^ (Vis), 3.2 × 10^−5^ (white)	2.55 × 10^[^ ^9^ ^]^ − 10^14^ (Vis)	^[^ ^153^, ^156^, ^157^ ^]^
	WS_2_	Direct exfoliation	Ultrasonication	Polymer	N/A	N/A	^[^ ^162^ ^]^
		Direct exfoliation	Ultrasonication	None	1.78 × 10^−2^ (Vis), 1.6 × 10^−4^ (white)	5.86 × 10^[^ ^10^ ^]^ (Vis)	^[^ ^156^, ^163^ ^]^
	WSe_2_	Direct exfoliation	Ultrasonication	Polymer	N/A	N/A	^[^ ^162^ ^]^
Transition metal dichalcogenides (TMD)	WTe_2_	Direct exfoliation	Ultrasonication	None	8 × 10^−2^ (white)	N/A	^[^ ^156^ ^]^
	ReS_2_, ZrTe_2_, NbSe_2_	Direct exfoliation	Ultrasonication	Polymer	N/A	N/A	^[^ ^162^ ^]^
Layered III–VI semiconductors	GaTe	Direct exfoliation	Ultrasonication	None	0.2 (Vis)	N/A	^[^ ^164^ ^]^
	InSe	Direct exfoliation	Ultrasonication	None	10–274 (Vis)	N/A	^[^ ^165^, ^166^ ^]^
	In_2_Se_3_	Electrochemical exfoliation		None	1.607 × 10^−3^ − 6.25 × 10^−3^ (Vis), 7.14 × 10^−4^ (NIR)	N/A	^[^ ^167^ ^]^
Topological insulators	Bi_2_Se_3_	Direct exfoliation	Ultrasonication	None	2.8 × 10^−5^ (NIR)	N/A	^[^ ^168^ ^]^
MXenes	Ti_3_C_2_T*_x_*	Chemical exfoliation (etching)		TiO_2_ nanoparticles	N/A	N/A	^[^ ^169^ ^]^
		Chemical exfoliation (etching)	Ultrasonication	CsPbBr_3_ perovskite nanocrystals	N/A	N/A	^[^ ^170^ ^]^
		Chemical exfoliation (etching)	Ultrasonication	Zn_2_GeO_4_ nanowires	2.04 × 10^−2^ (UV)	N/A	^[^ ^171^ ^]^
MXenes	Mo_2_CT*_x_*	Chemical exfoliation (etching + intercalation)	Ultrasonication	None	9 (Vis)	4.7 × 10^[^ ^11^ ^]^ (Vis)	^[^ ^172^ ^]^

## 2 Liquid Exfoliation Methods

To produce large quantities of high quality exfoliated 2D materials in a commercially viable manner, liquid exfoliation methods are highly promising and highly scalable methods available that can be done in mild conditions.^[^ ^58^, ^59^ ^]^ These methods span broad range of methods and can be categorized based on whether chemical reaction is involved or absent, which are direct exfoliation, which does not involve chemical rection and chemical exfoliation, which involves chemical reaction to assist in the exfoliation process. Liquid exfoliation methods can also be categorized based on the equipment used, which can be divided into three types: ultrasonic exfoliation,^[^ ^64^, ^173^, ^174^, ^175^, ^176^ ^]^ electrochemical exfoliation,^[^ ^177^, ^178^, ^179^, ^180^ ^]^ and shear exfoliation.^[^ ^181^, ^182^, ^183^, ^184^ ^]^ Readers interested in more extensive reviews of recent developments of liquid exfoliation of 2D materials methods are encouraged to refer to previous reviews on this subject.^[^ ^58^, ^59^, ^185^, ^186^, ^187^, ^188^ ^]^

### 2.1 Liquid Exfoliation Methods Based on Chemical Reaction

#### 2.1.1 Direct Exfoliation

Direct exfoliation refers to various liquid exfoliation methods that does not involve any chemical reactions.^[^ ^64^, ^150^, ^181^ ^]^ In other words, the exfoliation process is purely mechanical, without introducing any chemical and structural changes to the 2D materials upon exfoliation. Direct exfoliation methods have the advantage of retaining the original physical and electronic properties of the 2D materials, oftentimes resulting in superior qualities of the exfoliated nanosheets, with negligible amount of defects and chemical functionalization upon exfoliation. However, not all 2D materials can be easily exfoliated by direct methods. Only 2D materials that have weak van der Waals interlayer interaction can be exfoliated by direct exfoliation methods.

#### 2.1.2 Chemical Exfoliation

In chemical exfoliation, chemical reactions are intentionally introduced to facilitate the exfoliation process. There are several chemical reaction strategies that have been reported, such as oxidation reaction, intercalation, and chemical etching. Oxidation reaction is mainly used to oxidate graphite powders to obtain graphene oxide (GO), which is much more soluble in aqueous solution compared to pristine graphene.^[^ ^71^, ^72^, ^189^, ^190^, ^191^ ^]^ Functionalization of graphene layers with oxygen functional groups facilitates the exfoliation process and stabilize the GO nanosheet dispersion in aqueous solution. In intercalation reaction, chemical species are inserted into the interlayer spaces of the bulk 2D materials either by diffusion or the application of electrical potential. Oftentimes, charges are transferred between the intercalants and the host 2D materials, inducing phase transformation to the host material.^[^ ^158^, ^192^ ^]^ This phase transformation brings changes to the electrical properties of the materials and can be beneficial for certain applications, but can also degrade the performance for other applications. Chemical etching is useful for exfoliating 2D materials which are bonded by strong covalent or metallic bonds, therefore direct exfoliation is not useful in this case. In this method, selective layers of the bulk 2D materials are etched away, greatly weakening the interlayer interaction, facilitating the exfoliation process. Liquid exfoliation of MXenes have been mainly performed by selective etching of the A‐group element by variety of strong acids.^[^ ^45^, ^63^, ^193^ ^]^

### 2.2 Liquid Exfoliation Methods Based on the Equipment Used

#### 2.2.1 Ultrasonic Exfoliation in Liquid Medium

**Figure 1:**
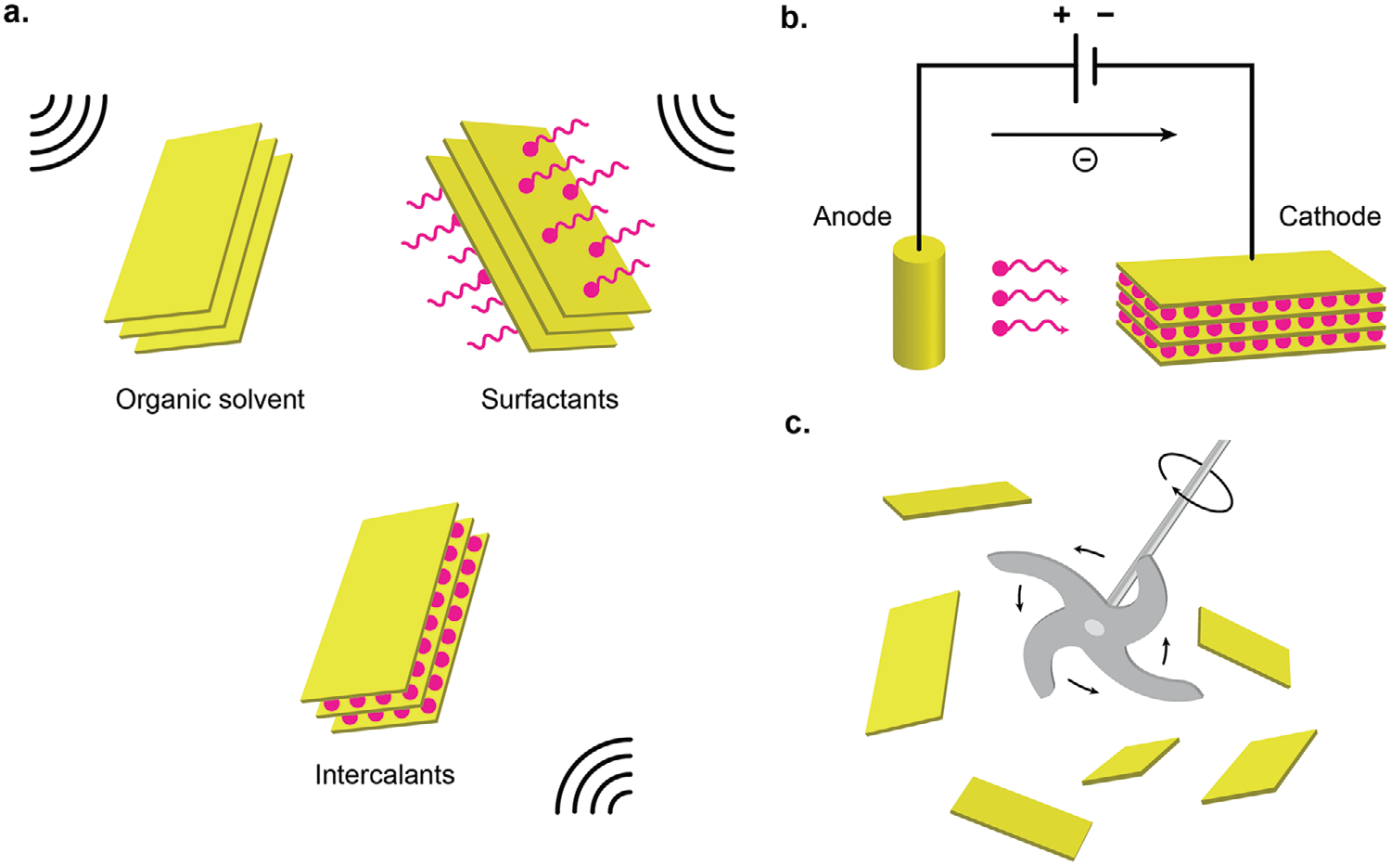
Variety of liquid exfoliation methods to produce 2D nanomaterials. a) Ultrasonic exfoliation in liquid medium. b) Electrochemical exfoliation. c) Shear exfoliation.

Ultrasonic exfoliation in liquid medium include a variety of methods, which rely on the liquid medium and the ultrasonic wave to exfoliate bulk 2D materials into nanosheets (**Figure** **1**a). The commonly used ultrasonic methods are bath sonication^[^ ^38^, ^64^, ^176^, ^194^ ^]^ and probe sonication^[^ ^173^, ^174^, ^195^ ^]^ methods. Ultrasonic waves in liquid media create bubbles or voids in the liquid which generate shear forces or cavitation bubbles upon collapsing, which then break up the bulk 2D materials into mono‐ and few‐layer nanosheets.^[^ ^196^ ^]^ The range of liquid media that are commonly used are organic solvents,^[^ ^64^, ^176^, ^197^, ^198^, ^199^, ^200^ ^]^ surfactants in aqueous media,^[^ ^194^, ^201^, ^202^, ^203^ ^]^ ionic liquids,^[^ ^204^, ^205^ ^]^ and solutions containing intercalants.^[^ ^206^, ^207^, ^208^, ^209^ ^]^ These liquid media play an important role in reducing the potential energy barrier between the interlayers of the 2D bulk materials and in stabilizing the nanosheet dispersions in the liquid media via interfacial interactions.

In organic solvent‐based exfoliation, the matching of the interfacial tensions (energies) between 2D materials and liquid media plays an important role in overcoming the interlayer van der Waals interaction and stabilizing the dispersion of the exfoliated nanosheets via solvent‐nanosheet interaction.^[^ ^38^, ^64^ ^]^ Hernandez et al. discovered that the concentration of graphene nanosheets was maximized when the surface tension (energy) of the organic solvents is ≈40 mJ m^−2^ (70 mJ m^−2^).^[^ ^176^ ^]^ Likewise, boron nitride (BN), molybdenum disulfide (MoS_2_), and tungsten disulfide (WS_2_) also seemed to possess surface energies of ≈70mJ m^−2^.^[^ ^64^ ^]^ Thus, the organic solvents that best results in the highest concentration of 2D materials must have surface tension that is ≈40 mJ m^−2^, such as *N*‐methyl‐2‐pyrrolidone (NMP ≈ 40 mJ m^−2^) and *N*,*N*‐dimethylformamide (DMF ≈ 37.1 mJ m^−2^).^[^ ^38^, ^59^, ^64^, ^176^ ^]^

Since organic solvents that are good for exfoliation are mostly toxic and have high‐boiling points such as NMP and DMF, surfactant‐assisted exfoliation in aqueous media is more appealing because it is more environmentally friendly and nontoxic. The surfactant molecules work as stabilizers that tune the surface tension of the liquid to facilitate better exfoliation and stabilize the exfoliated nanosheets by adsorbing to the nanosheet surface to prevent restacking.^[^ ^59^ ^]^ Surfactant‐assisted liquid exfoliation can be categorized based on the type of surfactant molecules: ionic and nonionic surfactants. In ionic surfactant exfoliation, the hydrophobic tail tends to adsorb to the non‐polar surfaces of the 2D materials such as graphene, while the hydrophilic head group tends to dissociate, forming a charge. This electrostatic charge stabilizes the dispersion by electrostatic repulsion between each other. The concentration of the dispersed nanosheets has been found to scale with the square of the zeta potential, *ζ*, which is proportional to the amount of charge on the surface.^[^ ^174^, ^194^, ^201^, ^210^, ^211^ ^]^ Ionic surfactants that have been used to exfoliate 2D materials include sodium dodecylbenzene sulfonate,^[^ ^201^ ^]^ sodium cholate (SC),^[^ ^174^, ^194^ ^]^ sodium deoxycholate,^[^ ^210^ ^]^ and 7,7,8,8‐tetracyanoquinodimethane.^[^ ^211^ ^]^ In non‐ionic surfactants, the mechanism of dispersion stabilization is attributed to the steric effects from the protruding hydrophilic tail which induces osmotic repulsion upon close contact, while the hydrophobic tail is adsorbed onto the nanosheets. The concentration has been shown to be scaled linearly with the steric repulsive potential barrier.^[^ ^202^, ^203^, ^212^ ^]^ Among the best non‐ionic surfactants that have been used to exfoliate 2D materials are P‐123^203^ and porphyrin.^[^ ^203^ ^]^

Ionic liquids (ILs) have attracted interest recently thanks to its unique properties such as high ionic conductivity, nonflammability, excellent thermal stability, and low vapor pressure.^[^ ^204^ ^]^ They are liquid semiorganic salts with melting point below 100 °C. Ionic liquids that have been used to exfoliate 2D materials include 1‐butyl‐3‐methyl‐imidazolium bis(trifl uoromethanesulfonyl)imide ([Bmim] [Tf2N]^[^ ^205^ ^]^ and 1‐hexyl‐3‐methylimidazolium hexafluorophos‐ phate.^[^ ^213^ ^]^

In intercalation‐assisted exfoliation, the insertion of intercalant molecules causes the interlayer spacings of the bulk layered materials greatly expands and the interlayer interaction weakened, making them easier to be exfoliated by the subsequent ultrasonication treatment. Several chemical species have been attempted, such as Li^+^,^[^ ^214^, ^215^, ^216^ ^]^ acids,^[^ ^208^, ^217^ ^]^ and organic molecules.^[^ ^209^ ^]^ The Li^+^ intercalation of TMD layered materials were first attempted by using *n*‐butyl lithium (BuLi).^[^ ^207^, ^214^, ^218^, ^219^ ^]^ In this process, Li^+^ ions are intercalated into bulk TMD powders by soaking them into hexane solution of BuLi for 48 h. Subsequently, the dried intercalated powder is reacted with water, which releases hydrogen gas that forces the layer apart, facilitating the exfoliation.^[^ ^219^ ^]^ Since Li+ intercalation process is highly sensitive to ambient conditions, the entire procedure must be conducted in an inert gas‐filled glovebox. In acid intercalation, charge transfer usually takes place, which results in partial oxidation, reduction, or covalent modification of the parent 2D materials.^[^ ^208^, ^220^ ^]^ However, Kovtyukhova et al. reported a successful and reversible nonoxidative intercalation of BN by Bronsted acids including H_2_SO_4_, H_3_PO_4_, and HClO_4_.^[^ ^217^ ^]^ Oxidation intercalation of BN only happens when treated with extremely oxidizing agents. Kovtyukhova et al. also studied the intercalation of graphite by Bronsted acids.^[^ ^208^ ^]^ The intercalated bulk crystals are readily exfoliated into mono‐and few‐layered nanosheets. The use of organic molecules for intercalation is usually done for exfoliation of layered ternary MAX phases to obtain 2D MXenes.^[^ ^193^, ^209^, ^221^, ^222^ ^]^ After the removal of the A element by selective etching, the weak bonds MX layers allow the intercalation of variety of organic molecules. The intercalation of hydrazine, dimethyl sulfoxide (DMSO), and urea into Ti_3_C_2_ has been reported which resulted in increase of interlayer distance, which facilitate the exfoliation upon weak sonication in deionized water.^[^ ^209^ ^]^

#### 2.2.2 Electrochemical Exfoliation

In electrochemical exfoliation setup, the bulk layered 2D materials are intercalated by the ionic species under an electrochemically applied bias (Figure 1b). Once intercalated, the bulk 2D bulk materials are then mildly ultrasonicated to complete the exfoliation. The intercalated bulk materials are much easier to be exfoliated by the ultrasonication, where it typically requires only minutes to hours, compared to direct ultrasonication which typically requires several days. Other advantages of the electrochemical exfoliation include simple operation setup, applicability in ambient conditions, and higher production scale on the order of milligrams to grams.^[^ ^186^ ^]^ The ionic species that are used to intercalate the 2D materials can be either anionic^[^ ^178^, ^179^, ^223^ ^]^ or cationic.^[^ ^180^, ^224^ ^]^

In anionic intercalation method, the experimental setup consists of using the bulk layered material as the anode electrode and another material (such as Pt) as the counter electrode. Large number of electrolytes have been investigated, such as the use of acids (HBr, HCl, HNO_3_, and H_2_SO_4_) for graphene exfoliation,^[^ ^178^, ^179^ ^]^ and the use of inorganic salts (such as Na_2_SO_4_ and KSO_4_) for graphene and MoS_2_ exfoliation.^[^ ^225^, ^226^ ^]^ For acid intercalation, KOH is usually added to lessen the oxidation effect of the acid, suppressing the generation of defects on the exfoliated 2D materials.^[^ ^178^ ^]^ In cationic intercalation, the layered material is used as the cathode, which has been shown to produce higher quality graphene with lower content of defects and oxygen functional groups compared to anionic intercalation,^[^ ^224^, ^227^ ^]^ because anionic intercalation usually requires higher potentials than oxidation potential of graphite.^[^ ^186^ ^]^ Several cationic species has been investigated, such as Na^+^ for graphene exfoliation,^[^ ^224^, ^227^ ^]^ Li^+^ for MoS_2_, WS_2_, TiS_2_, TaS_2_, ZrS_2_, and graphene exfoliation.^[^ ^180^ ^]^ To produce the highest exfoliation yield while retaining the good quality of the exfoliated 2D materials (e.g., avoiding over‐oxidation), experimental parameters such as the optimized electrolyte concentration, the magnitude of the applied potentials and the exfoliation time must be carefully considered.

#### 2.2.3 Shear Exfoliation

In shear exfoliation method, a shear mixer (or kitchen blender) which consists of a rotor and a stator is utilized to generate a high shear rate into the liquid media, in which the bulk 2D materials powder is mixed (Figure 1c).^[^ ^181^, ^228^, ^229^ ^]^ It was reported for graphene that the exfoliation start to occur once the local shear rate exceeded 10^4^ s^−1^,^[^ ^181^ ^]^ and for MoS_2_ and WS_2_, the value is 3 × 10^4^ s^−1^.^[^ ^182^ ^]^ It was reported that this shear exfoliation method could be applied in liquid volume as large as hundreds of liters with the production rate of 1.44 g h^−1^ for graphene^[^ ^181^ ^]^ and 0.95 g h^−1^ for WS_2_,^[^ ^182^ ^]^ much higher than previously reported for ultrasonication methods.

It is important to understand the parameters that control the exfoliation yield to maximize the production rate. It was discovered that the scalability of this shear exfoliation method was intimately related to variables such as initial 2D materials concentration *C*_i_, mixing time *t*, rotor diameter *D*, liquid volume *V*, and rotor speed *N*. Quantitatively, the concentration of the exfoliated 2D materials C can be empirically expressed as:

(1) $$C\propto C_{i}^{\chi }t^{\tau }N^{n}D^{d}V^{v}$$

where the exponential parameters *χ*, *τ*, *n*, *d*, and *v* were obtained empirically.^[^ ^181^, ^182^ ^]^ These values were found to be 1 (*χ*), 0.66 (*τ*), 1.13 (*n*), 2.28 (*d*), and −0.68 (*v*) for shear mixing of graphene and MoS_2_. The production rate, *P*_R_, can be expressed as:^[^ ^181^, ^182^ ^]^

(2) $$P_{R}=\frac{CV}{t}\propto C_{i}^{\chi }t^{\tau -1}N^{n}D^{d}V^{v+1}$$

Since *v* > −1, the production rate can be increased by increasing the mixing volume.^[^ ^181^, ^182^ ^]^ This is in contrast with the ultrasonication method, in which the *P*_R_ cannot be increased by increasing the liquid volume.^[^ ^181^ ^]^ Therefore, shear exfoliation has better scalability compared to ultrasonication method.

## 3 Size Selection of 2D Nanosheets

**Figure 2:**
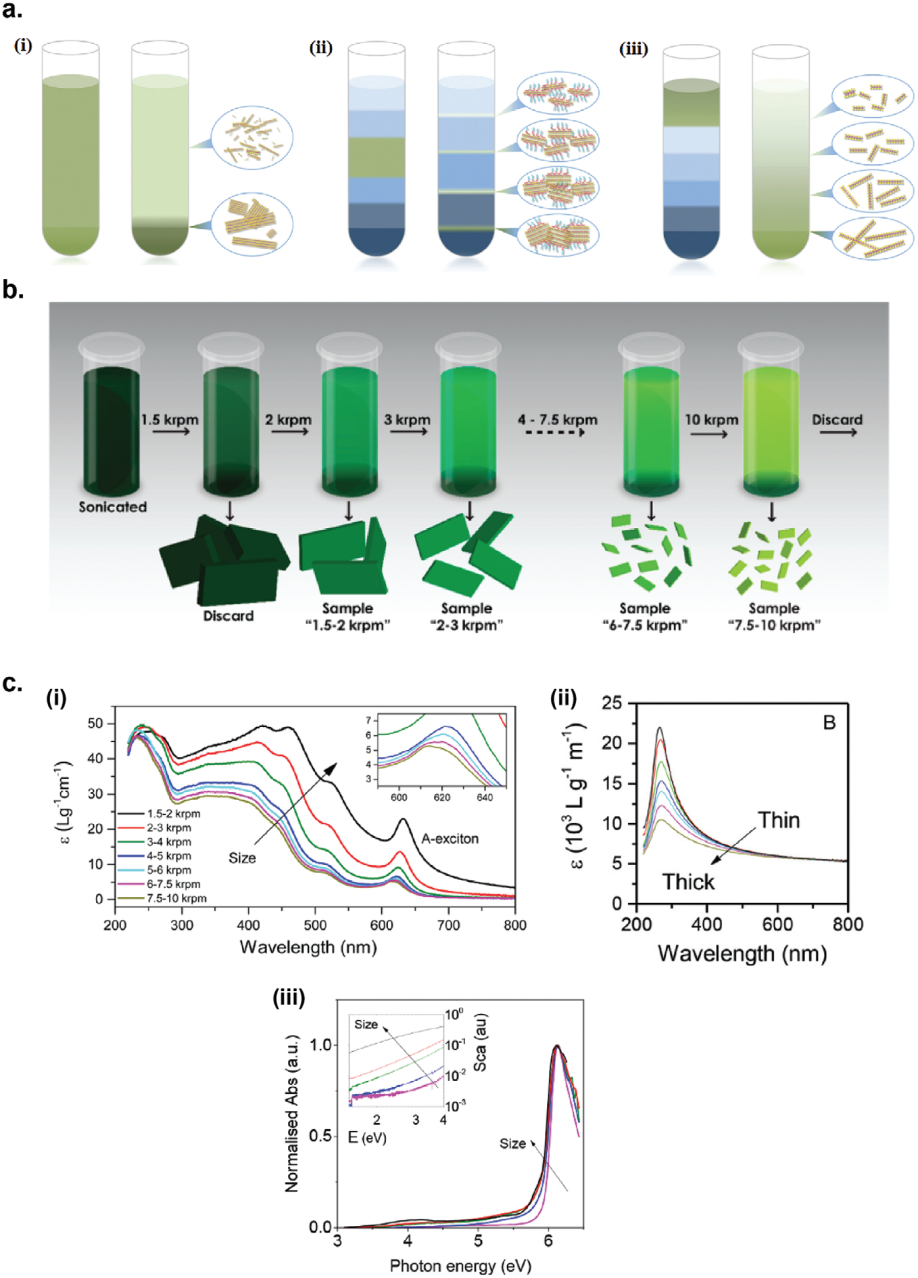
Size selection methods for liquid exfoliated 2D materials nanosheets. a) Three categories of centrifugation methods: i) sedimentation‐based separation or centrifugation (SBS), ii) sedimentation‐based density gradient ultracentrifugation (sDGU), and iii) isopycnic density gradient ultracentrifugation (iDGU). Reproduced with permission.^[^ ^233^ ^]^ Copyright 2016, WILEY‐VCH. b) Schematic illustration of the cascade centrifugation method. Reproduced with permission.^[^ ^236^ ^]^ Copyright 2016, American Chemical Society. c) Change in the shape of UV–vis spectra depending on the average size/thickness of the nanosheets: i) WS_2_, ii) graphene, and iii) boron nitride. i) Reproduced with permission.^[^ ^236^ ^]^ Copyright 2016, American Chemical Society. ii) Reproduced with permission.^[^ ^237^ ^]^ Copyright 2016, Royal Society of Chemistry. iii) Reproduced with permission.^[^ ^238^ ^]^ Copyright 2018, American Chemical Society.

Once exfoliated in a large scale by one of the liquid exfoliation methods above, the as‐exfoliated 2D nanosheets dispersion contains a wide range of lateral sizes and thicknesses.^[^ ^230^, ^231^ ^]^ Since physical properties of 2D materials such as bandgap and catalytic properties are strongly dependent on the average layer number and size of the nanosheet dispersions, sorting of these nanosheets to obtain monodisperse dispersion is desirable if one desires to obtain specific physical properties of 2D materials for a certain application. To achieve that end, centrifugation method is commonly used to controllably separate 2D nanosheets based on size and thickness.^[^ ^230^, ^231^, ^232^ ^]^ Centrifugation method can be broadly separated into three categories: 1) sedimentation‐based separation or centrifugation (SBS) which separates nanosheets based on their different masses; 2) sedimentation‐based density gradient ultracentrifugation (sDGU), which also separates nanosheets based on their masses, but with more precise lateral size separation via controlled density gradient; and 3) isopycnic DGU (iDGU), which separates nanosheets based on their buoyant density (**Figure** **2**a).^[^ ^233^ ^]^

### 3.1 Sedimentation‐Based Separation (SBS)

SBS is the simplest and most widely used centrifugation technique (Figure 2a(i)).^[^ ^234^ ^]^ It is performed by using a liquid medium with constant physical properties (density and viscosity), such as pure organic solvents. In a dispersion of polydisperse 2D materials nanosheets, thicker and larger nanosheets sediment faster compared to thinner and smaller nanosheets, due to their larger mass to surface area ratio. In general, higher centrifugation speed will increase the sedimentation rate regardless of the particle size. The rate of sedimentation is governed by the Stoke's law, which depends on the nanosheet size/thickness, solvent's viscosity, solvent's density, and the centrifugation speed^[^ ^235^ ^]^

(3) $$v=\frac{2\left(\rho_{s}-\rho_{l}\right)ar^{2}}{\eta }$$

where *v* is the solute sedimentation speed during centrifugation, *ρ*_s_ is the nanosheet density, *ρ*_l_ is the solvent density, a is the centrifugal acceleration, *r* is the effective radius of the solute, and *η* is the solvent viscosity.

The SBS method can be used iteratively to produce nanosheet dispersions with narrow size/thickness distributions. Backes et al. also introduced the cascade centrifugation method, based on the iteration of SBS centrifugation with increasing speed, which allowed for a highly efficient size/thickness selection of 2D nanosheets (Figure 2b).^[^ ^236^, ^237^, ^238^ ^]^ This method has been utilized to obtain size selection of variety of liquid‐exfoliated 2D materials including WS_2_,^[^ ^236^ ^]^ graphene,^[^ ^237^ ^]^ and boron nitride.^[^ ^238^ ^]^ Interestingly, the size selected dispersions of 2D nanosheets exhibit changing extinction spectra depending on the average size/thickness of the nanosheets contained in the dispersion (Figure 2c).^[^ ^232^, ^236^, ^237^, ^238^ ^]^ This allows a simple in situ spectroscopic method to determine the average size/thickness distribution of 2D nanosheets in a given dispersion without the necessity to perform microscopic analysis.

### 3.2 Sedimentation‐Based Density Gradient Ultracentrifugation (sDGU)

For sDGU, the liquid medium used has a gradient of density and viscosity, which allows for a more controlled sedimentation rates for nanosheets with different sizes upon centrifugation, and also to avoid cross contamination of nanosheets between different sizes (Figure 2a(ii)).^[^ ^230^, ^232^ ^]^ In this method, the dispersion of 2D nanosheets is placed on top of the density gradient medium, and upon centrifugation the 2D nanosheets sediment with different sedimentation rates through the density gradient medium based on their size/thickness.

The use of sDGU method for 2D nanosheet sorting was demonstrated by Backes et al. to produce dispersions of MoS_2_ nanosheets with different thickness and lateral size distributions.^[^ ^232^ ^]^ The as‐exfoliated MoS_2_ stock dispersion was placed on top of a race layer of higher density and then subjected to short centrifugation, which led to the spreading of the nanosheets across the centrifuge vial where the nanosheet mass increased from top to bottom. Subsequent fractionation allowed the collection of MoS_2_ nanosheets with specified size/thickness distribution. This technique is general and was also applied to other materials including WS_2_, MoSe_2_, and WSe_2_.^[^ ^232^ ^]^

### 3.3 Isopycnic Density Gradient Ultracentrifugation (iDGU)

While SBS and sDGU methods are based on mass sedimentation, iDGU is mainly based of the buoyant density of the nanosheets (Figure 2a(iii)).^[^ ^230^ ^]^ In this method, 2D materials nanosheets covered by surfactants sediment across the liquid density gradient until they arrive at their isopycnic points. Buoyant density is mainly determined by the thickness of the nanosheets and not by their lateral size, making this method suitable for thickness sorting of 2D materials.

The use of iDGU for thickness sorting of 2D materials was first demonstrated by Green et al. for sorting graphene nanosheets.^[^ ^239^ ^]^ Sodium cholate (SC) was used to encapsulate graphene nanosheets which separated into multiple bands which contains graphene nanosheets with different thickness upon centrifugation.^[^ ^239^ ^]^ iDGU method was also demonstrated for sorting out other 2D materials, such as BN,^[^ ^240^ ^]^ MoS_2_,^[^ ^241^ ^]^ and ReS_2_.^[^ ^242^ ^]^

## 4 Methods of Deposition

The as‐exfoliated or size‐selected 2D materials dispersions can then be directly deposited on a substrate or can be subsequently formulated into a highly concentrated functional ink for device printing application.^[^ ^243^, ^244^ ^]^ There are variety of deposition methods for fabrication of devices, which include drop casting,^[^ ^180^, ^245^ ^]^ dip coating,^[^ ^246^, ^247^ ^]^ spin‐coating,^[^ ^248^, ^249^ ^]^ spray coating,^[^ ^250^, ^251^ ^]^ and various printing methods (inkjet and electrohydrodynamic printing).^[^ ^151^, ^153^, ^243^, ^252^ ^]^

### 4.1 Drop Casting

In drop casting, drops of 2D materials dispersion is cast onto a target substrate, and then heated or subjected to vacuum to remove the solvent. Since it does not require any equipment for the process, it is the simplest deposition method and thus widely used in laboratory settings. The thickness of the drop‐casted film can be controlled by changing the concentration of the dispersion and the number of drops. Drop casting has been applied in many 2D materials dispersions, such as graphene,^[^ ^253^ ^]^ black phosphorus,^[^ ^254^ ^]^ MoS_2_,^[^ ^255^ ^]^ WS_2_,^[^ ^256^ ^]^ InSe,^[^ ^257^ ^]^ and Bi_2_S_3_.^[^ ^258^ ^]^ Shortcomings of this simple deposition method include the difficulty in precisely controlling the thickness and uniformity of the film and inability to obtain large‐area thin film.^[^ ^6^, ^259^ ^]^ Since controlling the thickness of the thin film down to tens of nanometer is nearly impossible by drop‐casting method, this technique is not suitable for variety of optoelectronic applications.^[^ ^259^ ^]^

### 4.2 Dip Coating

In dip coating method, a dispersion of 2D materials is prepared in a container into which a target substrate is immersed for a short time and then pulled back to dry. Typically, the immersion direction is vertical, but different angle of immersion can also be done to obtain asymmetrical deposition thicknesses. When pulling the substrate out from the dispersion, the speed at which the substrate is pulled is important. High pulling speed typically results in thicker films. The concentration of the dispersion also determines the quality of the deposited film. Very low concentration leads to uneven deposition, while very high concentration leads to aggregation of nanoparticles, causing multilayer assembly. During the drying stage, which usually assisted by heating, drying time and temperature required depends on the boiling point of the solvent used. Dip coating method has been applied to variety of 2D materials such as graphene,^[^ ^247^ ^]^ TMDs,^[^ ^67^, ^260^ ^]^ and MXenes.^[^ ^261^, ^262^ ^]^

### 4.3 Spin Coating

In spin coating, the dispersion of 2D materials is spun at a very high speed on a target substrate, creating a uniform thin film due to the centripetal force and surface tension of the solvent. This method has the advantage of producing a very uniform thin film very quickly and easily. The thickness of the film can be controlled by rotation speed, duration, liquid quantity, concentration, and viscosity. Repeating the spin‐coating process can increase the thickness of the deposited film. One major shortcoming of this method, especially for expensive nanomaterials, is the waste of the liquid material expelled during spinning. Spin‐coating has been applied to form 2D materials thin films, including graphene,^[^ ^248^ ^]^ MoS_2_,^[^ ^263^, ^264^ ^]^ InSe,^[^ ^264^ ^]^ Bi_2_S_3_,^[^ ^265^ ^]^ and Bi_2_Te_3_.^[^ ^266^ ^]^

### 4.4 Spray Coating

In spray coating, the 2D materials dispersion is atomized into uniform and fine droplets with the assistance of gas pressure or high electric voltage, and then uniformly coated into a target substrate to form thin film. By using a mask superimposed on the substrate, the location of the coating can be controlled, useful for patterning the thin film. This method is derived from the existing industrial processes and has a great potential for large‐area and low‐cost deposition. However, large amount of waste can be generated during the spray‐coating process, making it unsuitable for rare or expensive nanomaterials. 2D materials that have been used for spray‐coating deposition include graphene^[^ ^267^ ^]^ and TMDs.^[^ ^250^, ^268^ ^]^ One form of spray‐coating method, the dynamic spray‐gun method, has been used to deposit graphene and its composites with other nanomaterials for various electronic and energy devices, including supercapacitors and random access memories.^[^ ^269^ ^]^

### 4.5 Inkjet Printing

Inkjet printing is a non‐contact printing technique where rapid succession of ink droplets are jetted and deposited onto a target substrate to form a predefined pattern.^[^ ^243^ ^]^ There are two types of jetting mechanisms available for inkjet printing: continuous inkjet (CIJ) and drop‐on‐demand inkjet (DoD).^[^ ^270^ ^]^ In CIJ technique, the ink droplets are continuously generated and ejected, assisted by the electrostatic field deflecting the charged droplets on the substrate. In DoD technique, the ink droplets are only ejected when demanded by means of piezoelectric or thermal activation process. Even though CIJ technique allows for higher jetting speed and efficiency, the difficulty in controlling the jetting process and recycling issues of the inks have prevented them for widespread use. On the other hand, DoD technique has become the mainstream inkjet printing technology due to its simpler operation compared to CIJ technique.^[^ ^243^ ^]^ Liquid‐exfoliated 2D materials have been demonstrated for variety of device structures fabricated from inkjet printing, such as graphene,^[^ ^152^, ^271^ ^]^ BP,^[^ ^272^, ^273^ ^]^ and MoS_2_.^[^ ^37^, ^151^ ^]^

### 4.6 Electrohydrodynamic Printing

Electrohydrodynamic (EHD) printing technique is a high‐resolution printing method, which can generate printing features much smaller than the nozzle size.^[^ ^274^, ^275^ ^]^ During the printing process, the electric field applied between the nozzle tip and substrate causes the mobile ions in the ink to accumulate at the surface, deforming the meniscus at the nozzle end into a conical shape (Taylor cone).^[^ ^276^ ^]^ When the repulsion between charged ions at the cone apex becomes larger than the surface tension by increasing the electric field, a jet of the fluid is printed onto the substrate. Since the diameter of the jet is much smaller than the nozzle size, EHD printing can generate printing patterns with much better resolution than inkjet printing technique, which could reach micro‐ to nanoscale resolutions.^[^ ^276^ ^]^ EHD printing has been applied for wide range of 2D materials, such as graphene,^[^ ^277^, ^278^, ^279^ ^]^ BN,^[^ ^279^ ^]^ MoS_2_,^[^ ^28^, ^278^, ^279^ ^]^ and WS_2._^[^ ^28^, ^278^ ^]^

## 5 Metal–Semiconductor–Metal Photodetector

**Figure 3:**
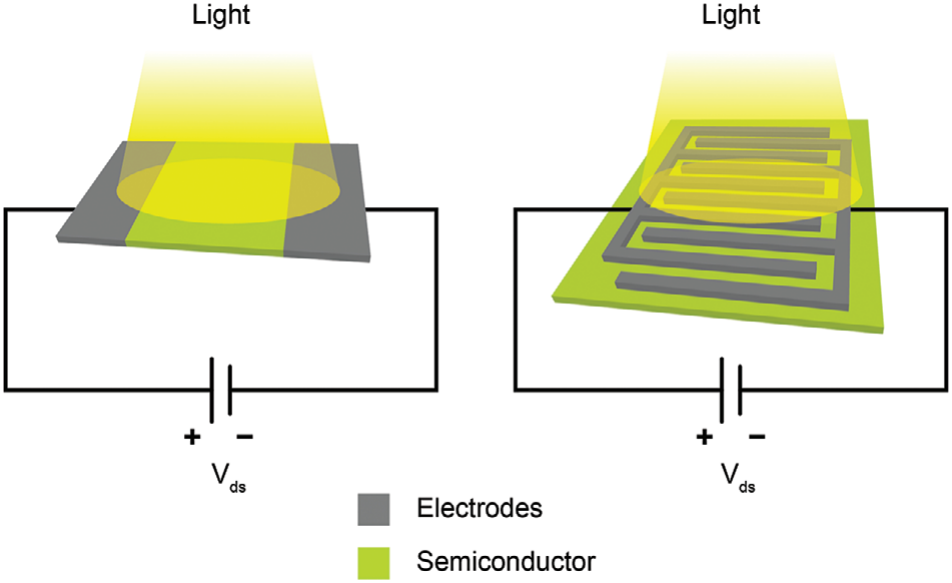
Schematics of different metal–semiconductor–metal (MSM) photodetector configurations, with i) parallel electrodes and ii) interdigitated electrodes.

In a MSM photodetector design, the device contains two Schottky contacts, that is, two metallic electrodes placed across each other on a semiconductor channel material. The electrode configuration can either be parallel or interdigitated electrode structure (**Figure** **3**a,b, respectively). The use of interdigitated electrodes is beneficial because the area of the photoactive channel can be maintained while the distance between the electrodes can be made smaller, increasing the device speed and sensitivity, without the necessity to apply high source–drain bias. During the operation, light is absorbed in the semiconductor channel area, creating the photogenerated charge carriers (electrons and holes). At the same time, source–drain bias is applied across the electrodes, driving the photogenerated carriers to be collected at the electrodes. By incorporating gate and dielectric layers into the device structure, MSM photodetectors can also function as phototransistors.^[^ ^80^ ^]^ By applying the gate voltage, the noise signal can be reduced and photocurrent signals amplified, improving the responsivity and gain.^[^ ^6^ ^]^

Other than MSM photodetector structure, liquid exfoliation of 2D materials have also been applied in other photodetector structures, such as photodiode^[^ ^280^, ^281^, ^282^, ^283^, ^284^ ^]^ and photoelectrochemical (PEC) detectors.^[^ ^69^, ^70^, ^285^, ^286^, ^287^ ^]^ In photodiode detectors, the photogenerated carriers (electrons and holes) are separated at the junction by the presence of built‐in potential, similar to photovoltaic devices. Then the separated electrons and holes are transported to the opposite electrodes. Oftentimes, a reverse bias is applied to increase the efficiency of charge collection. The photodiode detectors exhibit low dark current, fast photoresponse, and high charge collection efficiency even at low voltage.^[^ ^6^, ^7^ ^]^ However, due to vertical structure, careful control of the interface between different types of 2D materials must be done to ensure that redispersion is minimized.^[^ ^153^ ^]^ Also, the top electrode must be highly transparent, making the fabrication process more complicated compared to MSM photodetectors. In PEC detectors, the photogenerated electron–hole pairs in the photoactive materials are transferred to electrode surface and the interface with electrolyte solution, generating an oxidation–reduction reaction, creating a photocurrent.^[^ ^288^, ^289^ ^]^ PEC detectors exhibit high photosensitivity and low dark current. However, the requirement for electrolyte solution complicated the overall design, preventing them for monolithic integration within optoelectronic integrated circuits. Compared to other photodetector structures mentioned above, MSM photodetector design has a simple planar structure, which makes them compatible with the existing field effect transistor (FET) fabrication techniques. Therefore, they are more suitable for monolithic integration with other components of the optoelectronic integrated circuits.^[^ ^6^ ^]^

Below we describe the classifications of modes of photodetection mechanisms that might take place inside the photoactive channel that give rise to light‐induced electrical signal in the MSM photodetector. They can be broadly categorized into photovoltaic effect, bolometric effect, photothermoelectric effect, photogating effect, and plasmonic‐enhanced photodetection mechanism.

### 5.1 Mechanisms of Photodetections

#### 5.1.1 Photovoltaic Effect

**Figure 4:**
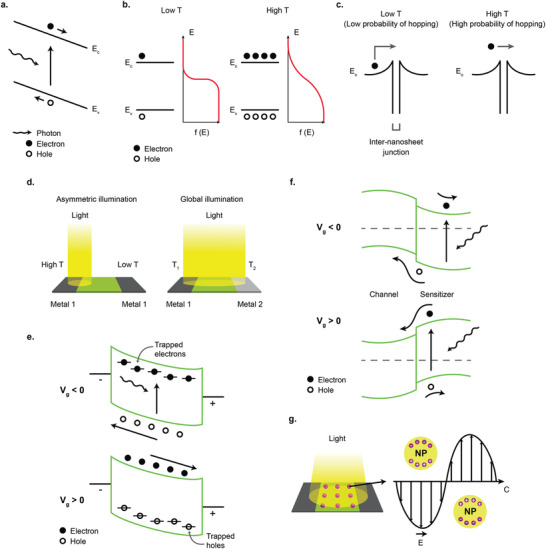
Different photodetection mechanisms. a) Photovoltaic effect. b) Bolometric effect via thermal excitation of electrons from valence band to conduction band. c) Increased probability of inter‐nanosheet carrier hopping due to photoinduced temperature increase. d) Photothermoelectric (PTE) effect due to asymmetric illumination (left) and asymmetric electrode configuration (right). e) Photogating effect due to carriers trapped in defects. f) Photogating effect due to trapped carriers in the sensitizer material. g) Plasmonic‐enhanced photodetection by metal nanoparticles.

Photovoltaic (PV) effect occurs when free electron–hole (e–h) pairs are generated by absorbed photons with energy higher than the bandgap of the photoactive material, and then separated either by a built‐in electric field at the junction or by the application of source–drain bias that creates an external electric field (**Figure** **4**a).^[^ ^290^ ^]^ The photogenerated electrons would be collected at the drain terminal, while the photogenerated holes would be collected at the source terminal.

#### 5.1.2 Bolometric Effect

The bolometric effect is the change of the resistivity of the photoactive materials due to the light induced heating. Almost all materials exhibit a well‐defined change in the resistivity as a function of temperature in a wide range of temperature range. For semiconductor materials, the simple explanation of the mechanism is the thermal activation of the electrons from valence band to the conduction band due to thermal activation (Figure 4b). The relation of the resistivity *ρ* of a semiconductor as a function of temperature *T* due to thermally excited electrons can be expressed as:^[^ ^291^ ^]^

(4) $$\rho \left(T\right)=\rho_{0}e^{E_{A}/k_{B}T}$$

where *E*_A_ is the activation energy and *k*_B_ is the Boltzmann constant. However, there are other factors that also contribute to the change in the material's resistivity by temperature increase, such as increased carrier scattering, vacancy formation, strain, etc, which makes the thorough analysis of the constituent mechanisms complicated. For photodetectors made from liquid‐exfoliated 2D materials, bolometric effect can also manifest from increased probability of inter‐nanosheet carrier hopping as the temperature is increased due to the photo‐induced heating (Figure 4c).^[^ ^292^ ^]^ Bolometric effect has been exploited for the detection of light in mid‐infrared range (3–8 µm) by using reduced graphene oxide (rGO) network and partly reduced graphene aerogel (PRGA).^[^ ^82^, ^83^, ^143^, ^293^, ^294^, ^295^ ^]^ The photon energies of mid‐infrared light is below the bandgap of rGO, which rules out the photovoltaic mechanism for mid‐infrared photodetection.

#### 5.1.3 Photothermoelectric Effect

Photothermoelectric (PTE) effect mechanism is based on both the photothermal conversion, which converts light energy into thermal energy and thermoelectric effect, which converts temperature difference into electric potential difference (Seeback effect).^[^ ^290^, ^296^ ^]^ The fundamental principle of the photodetecting mechanism is the net diffusion of charge carriers driven by the gradient of the charge carrier concentration or energy, due to light induced temperature gradient generated in the device. In an open circuit device configuration, the photovoltage *V*_PTE_ created by the photothermal induced hot carriers can be expressed as

(5) $$V_{\mathrm{PTE}}=\left(S_{1}-S_{2}\right)\Delta T$$

where *S*_1_ and *S*_2_ are the Seeback coefficients of two regions of the device, and Δ*T* is the temperature difference between two regions. The Seeback coefficient *S* of a material is strongly dependent on the material's electrical conductivity, *σ*, which can be expressed by the Mott formula^[^ ^297^ ^]^

(6) $$S=-\frac{\pi^{2}k_{B}^{2}T}{3e}{\left.\left(\frac{d\ln\sigma }{dE}\right)\right|}_{E=E_{F}}$$

where *k*_B_ is the Boltzmann constant, *e* is the elementary charge, and *E*_f_ is the Fermi energy. In order for the PTE effect to be dominant, an asymmetric temperature difference must be built up in the device, either by absorbing photons only on one side of the photodetector, or if global illumination is used, by the asymmetric device configuration (Figure 4d). By exploiting the PTE effect, the spectral sensitivity of the photodetector can be significantly extended from UV region into microwave region, as has been demonstrated by 3D graphene foam (3D‐GF) with asymmetric electrode configuration,^[^ ^144^ ^]^ enabling an ultra‐broadband photodetecting application.

#### 5.1.4 Photogating Effect

Photogating effect occurs when one type of the photogenerated carriers are trapped either by defects, impurities, or any hybrid structures, and in effect prolonging the excess carrier lifetimes of the other type of the photogenerated carriers.^[^ ^298^, ^299^ ^]^ The trapped photocarriers create an additional electric field that acts as a gate voltage that modulates the conductance of the device (Figure 4e). The magnitude of photocurrent *I*_ph_ can be roughly expressed as

(7) $$I_{\mathrm{ph}}=\frac{\partial I_{d}}{\partial V_{g}}\Delta V_{g}=g_{m}\Delta V_{g}$$

where *g*_m_ is the transconductance, *I*_d_ is the dark current, and *V*_g_ is the effective gate voltage exerted by the trapped photogenerated carriers. Due to the prolonged lifetime of the trapped carriers *τ*, photodetectors that operate on the photogating effect generally have limited response speed, but can have considerable responsivity.^[^ ^290^, ^300^, ^301^, ^302^ ^]^ Therefore, the tradeoff has to be made between the gain and bandwidth of the photodetector.

As the size of the photoactive materials decrease to nanometer scale, the role of individual defect and impurity as charge traps becomes more important, causing unpredictable photogating effect. The indication that the photogating effect is taking place can be observed from the light intensity dependence of photocurrent.^[^ ^299^ ^]^ Normally, when photogating effect exists, the light intensity dependence of photocurrent follows a power law with exponential factor *α* less than 1. This is due to the saturation of trapped photogenerated carriers with increasing light intensity, which reduces the incremental increase in the photoinduced gate voltage.^[^ ^299^ ^]^ It should be noted that other than photogating effect, carrier recombination processes can also influence the power dependence of photocurrent on light intensity, which are monomolecular process (when number of recombination traps greatly exceeds carrier concentration) if *α* = 1 and bimolecular process (when carrier concentration greatly exceeds recombination traps) if *α* = 0.5, or a combination of them if 0.5 < *α* <1.^[^ ^303^, ^304^ ^]^ The difference is that for photogating effect, mostly shallow traps are responsible for trapping charges, which do not lead to recombination; on the other hand, for bimolecular process, deep traps are mostly responsible for charge trapping, which lead to carrier recombination. Therefore, care should be taken when determining the origin of the sublinear dependence of photocurrent on light intensity.

In photodetectors made from liquid‐exfoliated 2D materials, the photogating effect can be the dominating mechanism when the 2D materials nanosheets channel are hybridized with sensitizer nanoparticles such as quantum dots, nanowires, and nanorods, where the photogenerated electron–hole pairs are separated at the interface between the two materials, and one type of photogenerated carriers remain or trapped for a prolonged time in one of the materials, creating an effective gate voltage (Figure 4f).^[^ ^89^, ^95^, ^98^, ^159^, ^160^ ^]^

#### 5.1.5 Plasmonic‐Enhanced Photodetection

Metallic nanostructures have the ability to constrain light by coupling the electromagnetic wave with the oscillations of the charged electrons at the surface of the metal.^[^ ^305^ ^]^ This excitation of surface electronic oscillations (surface plasmons) by the light leads to the formation of surface plasmon polaritons (SPP), which is a hybrid particle composed of the electronic oscillations and the electromagnetic wave. When coupled with a photoactive material of the photodetector, this confinement of the electromagnetic wave can enhance the electromagnetic field in the photoactive material, increasing the photoresponse (Figure 4g). The wavelength of the resonance and the extent of the light confinement can be adjusted depending on the shape and size of the metallic nanostructures. Because the resonation of the local plasmonic enhancement in the metallic nanostructures is occurring independently, the integration with 2D materials nanosheets can be done in a straightforward manner by depositing them directly from the solution or by mixing to form composite materials. This has allowed the applications of metallic nanoparticles to a variety of liquid‐exfoliated 2D materials for fabrication of plasmonic‐enhanced photodetectors.^[^ ^86^, ^87^, ^88^ ^]^

### 5.2 Charge Transport Mechanisms

#### 5.2.1 Variable‐Range Hopping

Variable‐range hopping (VRH) mechanism describes carrier transport in a disordered system;^[^ ^292^ ^]^ in the case of a network of 2D materials, the disorder stems from the random stacking of large number of 2D nanosheets. The signature of this transport mechanism can be seen in the dependence of conductivity *σ* on the temperature:^[^ ^292^ ^]^

(8) $$\sigma =\sigma_{0}e^{-{\left(T_{0}/T\right)}^{\beta }}$$

where *β* is a parameter which depends on the model used. Since the VRH is the main transport mechanism in a percolating network of 2D materials,^[^ ^306^, ^307^ ^]^ where the additional junction resistances between different nanosheets and possible recombination pathways at the junctions due to trap states, the magnitude of the photocurrents of photodetectors made of networks of 2D material nanosheets is generally smaller than photodetectors made of single‐crystalline 2D materials obtained from micromechanical exfoliation or CVD methods.

#### 5.2.2 Space‐Charge‐Limited Transport

Space‐charge‐limited‐current (SCLC) occurs when the injected carrier density *n* exceeds the intrinsic carrier density *n_0_* at high drain–source voltage (*V*_ds_).^[^ ^308^, ^309^ ^]^ Assuming the contacts are Ohmic, that results in the *I*–*V* characteristic deviating from the linear function. For example, in the Mott–Gurney regime where the materials is trap‐free, while the *I*–*V* characteristic display Ohmic relation at low *V*_ds_, at high *V*_ds_ the current becomes a quadratic function of the *V*_ds_.^[^ ^310^ ^]^ In the presence of trap distribution, the exponential factor can deviate from two.^[^ ^311^, ^312^ ^]^ Another signature of the SCLC mechanism is that the exponential factor increases monotonically as the temperature decreases.^[^ ^308^ ^]^

#### 5.2.3 Effect of Contacts

In MSM photodetector design, the electrodes are normally metallic or semi metallic materials. The contact between the semiconductor photoactive material and the metallic electrode (MS contact) is very important in determining the *I*–*V* characteristic of the device. There are basically two types of MS contacts: rectifying Schottky contact and non‐rectifying Ohmic contact. In ideal MS contact, in which the interface is sharp and clean and without any impurities or defects, the above types of MS contacts are determined by physical parameters of each materials, such as the work function, electron affinity, and bandgap. The Schottky barrier contact forms when there is a large potential barrier height for either electron or hole. This results in *I*–*V* characteristic of the MSM photodetector deviating from linear function of the voltage, and instead becomes an exponential function due to the increasing number of carriers overcoming the potential barrier as the voltage increases. Ohmic contact forms when there is no or negligible potential barrier between the two materials. This type of contact has a low resistance and has the typical linear *I*–*V* characteristics. However, in real devices, obtaining perfectly clean and impurity/defect free contacts are very difficult, causing the MS barrier height to be pinned regardless of the metal work function.^[^ ^313^, ^314^ ^]^ This is due to the presence of high density of trap states at the interface, acting as electron/hole reservoir that readily accept/donate electrons from/to the metal upon contact, bringing the work function of the metal pinned at the charge neutrality point.^[^ ^313^, ^314^ ^]^ However, the research into heterostructures of 2D materials have made possible the realization of atomically‐clean interface, without the presence of dangling bonds and impurities, which greatly minimizes the effect of this Fermi level pinning.^[^ ^314^, ^315^ ^]^

### 5.3 Performance Parameters

Key performance parameters of photodetectors in general include photoresponsivity (*R*), external quantum efficiency (EQE), detectivity (D*), gain (*G*), and response time (*t*_R_).

#### 5.3.1 Photoresponsivity

Photoresponsivity (*R*) is the ratio of the photocurrent per unit area of the photodetector divided by the incident light power density at a given wavelength

(9) $$R=\frac{I_{\mathrm{ph}}}{\mathrm{PA}}=\frac{I_{\mathrm{light}}-I_{\mathrm{dark}}}{\mathrm{PA}}$$

where *I*_ph_ is the photocurrent, which is the difference between the current under illumination *I*_light_ and the dark current *I*_d_. The spectral response of the photodetector's responsivity as a function of the light wavelength usually follows the absorption spectrum of the photoactive semiconductor material.

#### 5.3.2 External Quantum Efficiency

External quantum efficiency (EQE) is the ratio of the number of charge carriers collected at the electrodes of the photodetector to the number of photons of a given energy incident on the photodetector, and can be expressed as

(10) $$\mathrm{EQE}=\frac{\mathrm{electrons}/\sec}{\mathrm{photons}/\sec}=\frac{Rhc}{e\lambda }\times 100\%$$

where *h* is the Planck constant, *c* is the speed of light, *e* is the electronic charge, and *λ* is the wavelength of the light in question.

#### 5.3.3 Detectivity

Detectivity D* is a normalized measure of the minimum detectable light power (the smallest detectable signal) of a photodetector. Noise from the dark current gives a fundamental limit on the detectivity. Detectivity is given by

(11) $$D*=\frac{\sqrt{A\Delta f}}{\mathrm{NEP}}$$

where *A* is the photoactive area of the photodetector, Δ*f* is the bandwidth, and NEP is the noise equivalent power in unit of Watts, which results in signal‐to‐noise ratio of 1 in a one Hertz output bandwidth. The NEP can be expressed as function of the responsivity *R* and the noise spectral density *S*_n_ of the dark current (in units of A Hz^−1/2^)

(12) $$\mathrm{NEP}=\frac{S_{n}}{R}$$

therefore, the detectivity can also be expressed in terms of responsivity *R* as

(13) $$D*=\frac{\sqrt{A\Delta f}R}{S_{n}}$$

If the noise from the dark current *I*_d_ is limited by the shot noise, the detectivity can be simply expressed as

(14) $$D*=\frac{R}{\sqrt{2eI_{d}}}$$

which is the highest limit of the detectivity if all other noises are absent.

#### 5.3.4 Gain

The gain of the photodetector, *G*, is defined as the number of photogenerated carriers collected by the electrodes per number of photogenerated e–h pairs, which is the amplification of the photocurrent due to current modulation by the photogating effect. *G* can also be expressed as the ratio between the carrier transit time *τ*_T_ and the trapped carrier lifetime *τ*, *G* = *τ* / *τ*_T_.^[^ ^300^, ^301^ ^]^ This expression can be understood as follows. Due to the applied source–drain bias, it takes an average transit time *τ*_T_ for the photogenerated carriers to drift across the photodetector. If the trapped carrier lifetime *τ* is longer than the transit time *τ*_T_, the free carriers will keep flowing around the photodetector multiple times before it recombines with the trapped carrier of the other type. This amplifies the magnitude of photocurrent detected by the device.

#### 5.3.5 Response Time

The photodetector response time (*t*_R_) is the time required for the photodetector to rise from 10% to 90% of the maximum photocurrent. The response speed of the photodetector can either be limited by the transit time between the opposite electrodes or by the recombination time of the photogenerated carriers. But, if the RC time constant of the device is larger than both the transit time and the recombination time of the carriers, the RC time constant will be the limiting factor that determines the response time. To decrease the response time (or increase the response speed), one can shorten the distance between the electrode (if *t*_R_ is limited by the transit time) or introduce high‐density recombination centers to shorten the recombination time (if *t*_R_ is limited by the recombination time). However, introducing high‐density recombination centers would lower the device sensitivity because more photogenerated carriers are recombined before being collected at the electrodes.

## 6 Recent Developments of Liquid‐Exfoliated 2D Photodetectors

### 6.1 Graphene Photodetectors

Graphene has offered extraordinary potential in a wide variety of applications due to its excellent electronic,^[^ ^316^, ^317^ ^]^ thermal,^[^ ^318^ ^]^ and mechanical properties.^[^ ^319^ ^]^ The room temperature mobility of single‐layer graphene was reported to be over 10 000 cm^2^ V^−1^ s^−1^, theoretically is a zero‐bandgap semiconductor and exhibits ballistic transport at submicrometer distances.^[^ ^320^ ^]^ Its high mobility, combined with its transparency and flexibility, has made graphene excellent material for transparent conducting electrodes (TCE).^[^ ^321^, ^322^ ^]^ Therefore, its electronic and optoelectronic properties are of particular interest for its use in optoelectronic applications, such as photodetectors.

Majority of the reports of liquid‐exfoliated graphene‐based photodetector devices were fabricated by exfoliation of graphene oxide (GO) nanosheets in aqueous solvents, where the GO were obtained by a modified Hummers method^[^ ^189^, ^190^, ^191^ ^]^ by oxidizing the starting graphite bulk powder.^[^ ^71^, ^72^, ^73^, ^74^, ^76^, ^78^, ^82^, ^83^, ^84^, ^293^, ^323^, ^324^, ^325^, ^326^, ^327^, ^328^, ^329^, ^330^ ^]^ Variety of oxygen‐containing functional groups on the basal planes and at the edge sites makes the GO highly hydrophilic and easily dispersible in water and sonicated to achieve the exfoliated state. For further optoelectronic device applications, chemical reduction and/or annealing of GO is required to restore the initial aromatic conjugation structure of graphene and to restore the electrical conductivity.^[^ ^189^, ^190^ ^]^ In spite of that, the electrical conductivity could not return to its initial value because certain amount of defects would still remain in the graphene sheet after chemical reduction and/or annealing. This type of liquid‐exfoliated graphene is generally called reduced graphene oxide (rGO). Another type of liquid‐exfoliated graphene are obtained by directly exfoliating the raw graphite powder in liquid, such as by sonication or shear mixing, to obtain a dispersion of few‐layer graphene nanosheets with various thickness and size, which is generally called few layer graphene (FLG).^[^ ^64^, ^181^, ^231^ ^]^ The advantages of using FLG is they generally have higher electrical conductivity compared to rGO, making the photocurrent collection much faster, thus increasing the operation speed of the device. However, since FLG are more pristine than rGO due to much less defects and residual functional groups, the bandgap is nearly zero, resulting in poor photoresponse due to ultrafast carrier recombination in the order of femtoseconds.^[^ ^331^, ^332^ ^]^ Therefore, the use of FLG in photodetecting application is usually combined with other photosensitive material that transfers charge upon photoexcitation, such as quantum dots and photoactive polymer, where FLG's role is to accelerate charge collection to the electrodes.^[^ ^138^ ^]^

**Figure 5:**
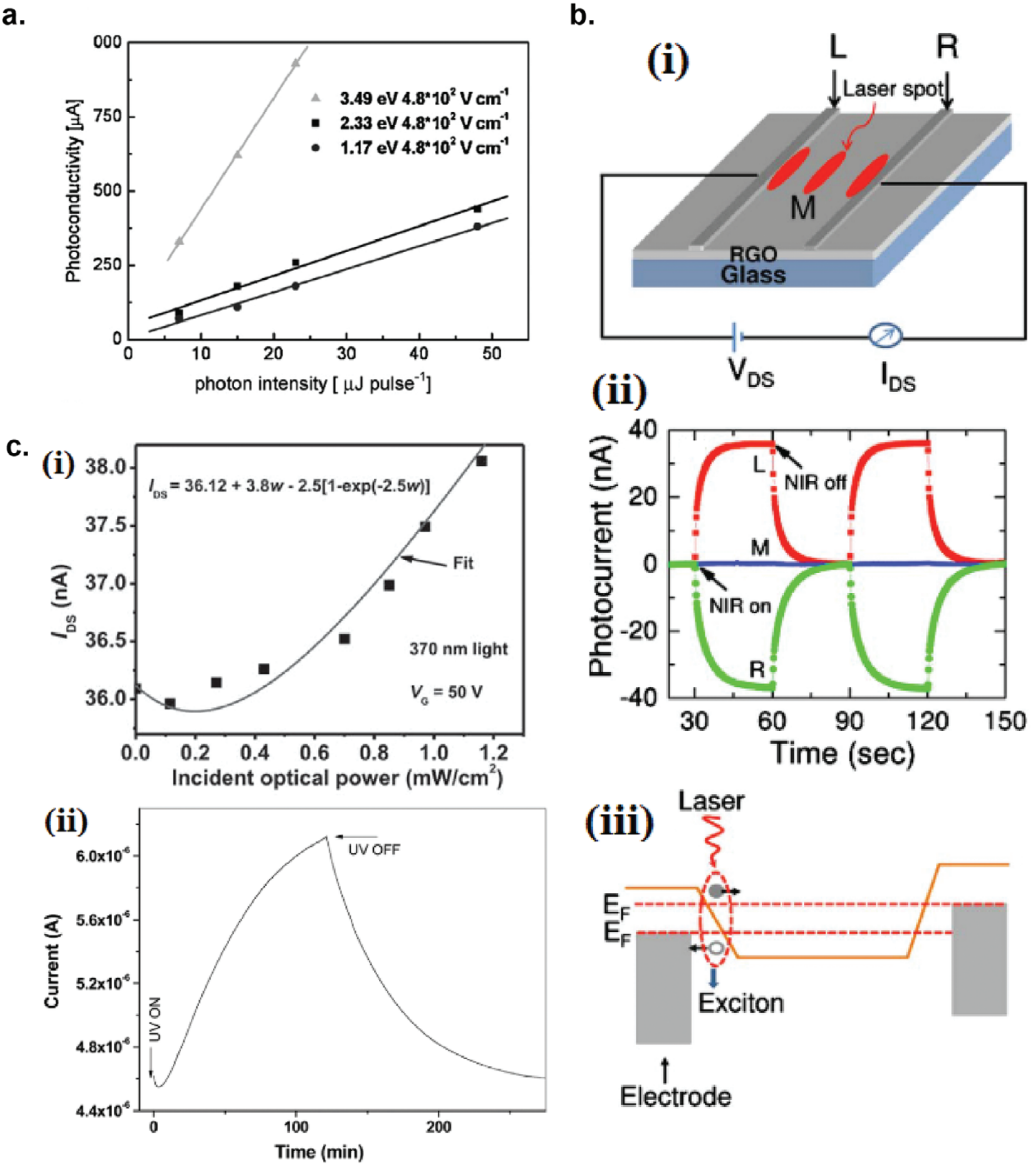
Photodetectors based on rGO. a) Incident light intensity dependence of photoresponse of drop‐casted rGO film with different photon energies. Reproduced with permission.^[^ ^71^ ^]^ Copyright 2009, Wiley‐VCH. b) Asymmetric photoresponse of rGO film. i) Schematic diagram of the photodetector and the experimental setup. L, M, and R denote the three different positions for NIR illumination. ii) Photocurrent transients for the rGO film under NIR illumination at positions L, M, and R. iii) Mechanism of photocurrent generation at the metallic electrode/rGO interface. Reproduced with permission.^[^ ^72^ ^]^ Copyright 2010, AIP. c) Negative photocurrent of rGO film at i) low light intensity and at ii) initial stage of photoresponse transient. i) Reproduced with permission.^[^ ^80^ ^]^ Copyright 2010, Wiley‐VCH. ii) Reproduced with permission.^[^ ^81^ ^]^ Copyright 2011, AIP.

Lv et al. first investigated the photoconductivity of the drop‐casted bulk reduced graphene oxide (rGO) film.^[^ ^71^ ^]^ **Figure** **5**a shows the incident light intensity dependence of photoresponse with different photon energies of the film. The graphene film has a strong photoresponse for all the wavelengths in the tested, because it has a wide absorption in a wide frequency range, unlike SWNTs which have a strong wavelength dependent absorption spectra.^[^ ^333^ ^]^ Indeed, ultrabroadband photodetection range from ultraviolet^[^ ^81^, ^325^, ^334^ ^]^ to infrared^[^ ^76^, ^79^, ^82^, ^83^, ^293^, ^334^, ^335^, ^336^ ^]^ have been demonstrated for rGO‐based photodetectors.

Ghosh et al. fabricated an rGO film photodetector and showed that the photocurrent either increased, decreased, or remained almost zero depending on the laser illumination position with respect to the electrodes (Figure 5b(i,ii)).^[^ ^72^ ^]^ This can be explained by the presence of a locally generated electric field near the rGO‐electrode interface due to Schottky barrier. The change in sign of photocurrent when illuminated at the vicinity of opposite electrodes is due to the opposite direction of electric fields at opposite electrodes (Figure 5b(iii)). Moon et al. also reached to similar results where the photovoltage is maximum when the laser illumination spot was near the rGO/electrode region, but minimum when illuminated at the center of the photodetector, due to the Schottky barrier‐induced band‐bending.^[^ ^78^ ^]^

Negative photoconductivity at low power irradiation^[^ ^79^, ^80^ ^]^ and/or at initial stage of photoresponse^[^ ^81^ ^]^ have been observed for rGO photodetector and followed by increase in photoconductivity at higher irradiation power and/or later stage of photoresponse (Figure 5c). This phenomenon can be explained by the competition between trapping of photoexcited electrons by the oxygenous functional groups, which tends to decrease the photocurrent, and the photogenerated electron–hole pairs, which tends to increase the photocurrent. At higher irradiation power and/or later stage of irradiation, the trapping sites are saturated, with the resultant net increase in the photocurrent.^[^ ^79^, ^80^, ^81^ ^]^ Such nonmonotonic optoelectronic behavior disappears when the reduction time of rGO was increased to 260 min, where oxygenous functional groups as electron trapping sites decreased significantly.

#### 6.1.1 Factors Influencing the Photocurrent of Graphene‐Based Photodetectors

There are several factors influencing the photocurrent responsivities of graphene‐based photodetectors: bandgap, type and level of doping, type of substrate, and annealing conditions.

##### Effect of Bandgap

Pristine graphene has very low photoresponses due to very fast photocarrier dynamics as a result of zero bandgap.^[^ ^79^, ^331^, ^333^ ^]^ Therefore, to exploit solution‐processed graphene films into devices, opening the bandgap of graphene for specific electronic and optoelectronic applications is becoming important. Two general approaches to open up the bandgap of graphene are nanostructuring of graphene and reduction of graphene oxide.^[^ ^337^, ^338^, ^339^ ^]^ Nanostructuring of graphene involves structuring of graphene into nanoribbons or nanodots in which the quantum confinement effects start to occur that increase the bandgap.^[^ ^337^ ^]^ Nanostructured water‐soluble graphene quantum dots (GQD) have been shown to exhibit absorption spectra that peaked at energies up to ≈6 eV, making them especially useful for UV photodetectors.^[^ ^340^ ^]^ On the other hand, reduction method involves converting the insulating GO into a more conducting material by either chemical or thermal reduction processes.^[^ ^189^, ^339^, ^341^ ^]^

**Figure 6:**
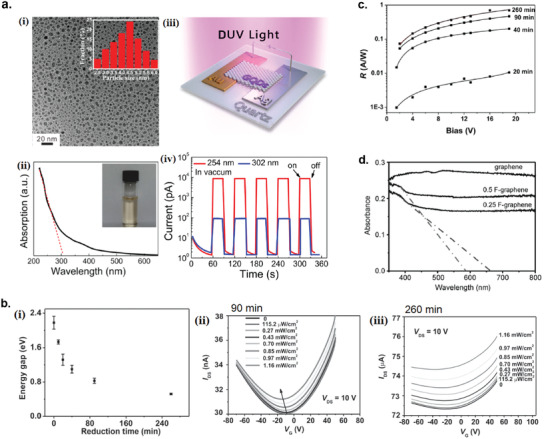
Bandgap engineering of graphene‐based photodetectors. a) GQD deep‐UV photodetector. i) TEM image of the GQDs. Inset: size distribution of the GQDs. ii) UV–vis absorption spectrum of the GQDs. Inset: photo of the aqueous solution of the GQDs. iii) Schematic of the GQD photodetector with asymmetric electrode configuration. iv) Photoresponse transients of the device under pulsed 254 and 302 nm DUV light (42 µW cm^−2^). The voltage bias was 5 V. Reproduced with permission.^[^ ^130^ ^]^ Copyright 2015, American Chemical Society. b) Controlling the bandgap of rGO by thermal reduction. i) The bandgap of rGO as a function of reduction time. ii) *I*_DS_–*V*_G_ curves of rGO FET thermally reduced for 90 min under irradiation at 370 nm. iii) *I*_DS_–*V*_G_ curves of rGO FET thermally reduced for 260 min under irradiation at 370 nm. Reproduced with permission.^[^ ^80^ ^]^ Copyright 2010, WILEY‐VCH. c) Responsivity as a function of bias for rGO/SiO_2_/Si‐based infrared photodetector based with different reduction times of rGO (0, 20, 40, 90, 260 min). Incident radiation power is ≈14 mW cm^−2^. Reproduced with permission.^[^ ^79^ ^]^ Copyright 2013, American Chemical Society. d) Diffuse reflectance spectroscopy of the as‐made graphene, 0.25 F‐graphene, and 0.5 F‐graphene. Reproduced with permission.^[^ ^136^ ^]^ Copyright 2011, WILEY‐VCH.

*Graphene Quantum Dot*: Photodetection of deep ultraviolet (DUV) at wavelength less than 320 nm has been an interesting topic due to various applications in important fields, such as chemical analysis, remote control, flame detection, and secure space‐to‐space communications.^[^ ^342^ ^]^ Zhang et al. fabricated a solution‐processed, large bandgap GQD photodetector that can detect DUV light with wavelength as short as 254 nm.^[^ ^130^ ^]^ The diameter of the GQD was in the range of 2.5–6 nm, which results in the greatly enhanced bandgap that was estimated to be ≈3.8 eV (**Figure** **6**a(i,ii)). On top of that, by introducing an asymmetric electrode structure Au–Ag, the photoresponsivity could be further increased by up to ≈500 times. The introduction of the asymmetric electrode structure increases the carrier collection at the electrodes under the forward bias direction, effectively increases the photocurrent and suppresses the carrier recombination (Figure 6a(iii,iv)). The device only responds to the DUV light and completely blind to the visible light, which exhibits the great superiority of GQD as solar‐blind DUV‐photodetector. The responsivity was estimated to be 2.1 mA W^−1^ and detectivity was 9.59 × 10^11^ cm Hz^1/2^ W^−1^ for illumination at 254 nm.^[^ ^130^ ^]^

Reduced graphene oxide QDs (rGOQDs) has a larger bandgap than GQDs, making it a promising deep‐UV photodetecting material.^[^ ^343^ ^]^ However, long response time of rGOQD photodetectors made it necessary to be measured under vacuum conditions.^[^ ^130^ ^]^ Nitrogen doping has been reported to greatly reduce the response time of rGOQDs.^[^ ^344^ ^]^ Zhang et al. synthesized a hybrid CVD‐grown graphene/N‐rGOQD photodetector which exhibited a high photoresponsivity of 1.8 × 10^3^ A W^−1^ and fast response time of 0.13 s under deep‐UV illumination (254 nm) and at ambient environment.^[^ ^131^ ^]^ The N‐rGOQDs were synthesized by the improved Hummers method and DMF hydrothermal treatment, while DMF was used as a reducing agent. Al_2_O_3_ capping layer was used to prevent molecule adsorption during operation in ambient environment. The synthesized N‐rGOQDs has strong photoresponse to deep UV light at 254 nm, but at 365 nm the photoresponse was not observable, which is advantageous for photodetection in solar‐blind field.

*Bandgap Engineering by Control of Thermal Reduction*: The bandgap of rGO can be precisely controlled by changing the thermal annealing condition for reduction.^[^ ^80^, ^329^, ^345^ ^]^ Velasco‐Soto et al. studied the modulation of the rGO bandgap by changing the reducing agents (glucose, fructose, and ascorbic acid), and successfully tuned the bandgap from 2.7 to 1.15 eV.^[^ ^345^ ^]^ Chang et al. reported a facile controlling of rGO bandgap ranging from 0.5 to 2.2 eV via a low‐temperature thermal reduction of liquid‐exfoliated graphene oxide nanosheets.^[^ ^80^ ^]^ Specifically, the bandgap of rGO can be tuned in a very precise manner by varying the annealing time at 150 °C (Figure 6b(i)). Figure 6b(ii,iii) shows the comparison of transfer characteristics of rGO phototransistors reduced at 90 and 260 min under varying illumination power at 370 nm light. rGO reduced at 260 min displayed less modulation in drain–source current *I*_ds_ under gate bias *V*_g_ because longer reduction time results in less oxygen‐containing functional groups, which makes rGO more metallic in nature. The effect of the different amount of oxygenated functional groups in rGO by different reduction time on the infrared responsivity is shown in Figure 6c. The longer reduction time of rGO results in significantly decreased density of oxygenated defects and larger area of ordered graphene sheets, which increase the device mobility and higher photogain, thus higher responsivity.^[^ ^79^ ^]^ Therefore, this facile and low‐temperature reduction method provides huge advantages for graphene‐based optoelectronic devices for large‐scale production.

*Bandgap Engineering by Fluorination*: Another method for bandgap opening of solution‐processed graphene is through fluorination of graphene nanosheets (F‐graphene).^[^ ^136^, ^346^, ^347^, ^348^ ^]^ Aguilar‐Bolados et al. attempted the fluorination of GO by using diethylaminodifluorosulfinium tetrafluoroborate, to replace the oxygen functional groups of GO by fluoride.^[^ ^346^ ^]^ This fluorination reaction modifies the bandgap of the material, increasing the band gap from 2.05 to 3.88 eV. Chang et al. reported that the bandgap of F‐graphene was widely tunable by varying the coverage and configuration of the fluorination.^[^ ^136^ ^]^ Ionic liquid was used to exfoliate the bulk F‐graphite powder due to the surface‐energy matching between the ionic liquid and the carbon sheet in F‐graphite. Figure 6d shows the changing of the bandgap of F‐graphene as a function of fluorination coverage by diffuse reflectance spectroscopy, where bandgap openings were observed for 0.25 F‐graphene and 0.5 F‐graphene but was not observed for graphene. The bandgap for 0.25 F‐graphene and 0.5 F‐graphene were 1.8 and 2.2 eV, respectively. The bandgap opening is comparable to that of rGO (Figure 6b(i)).

##### Effect of Doping on the Photocurrent

**Figure 7:**
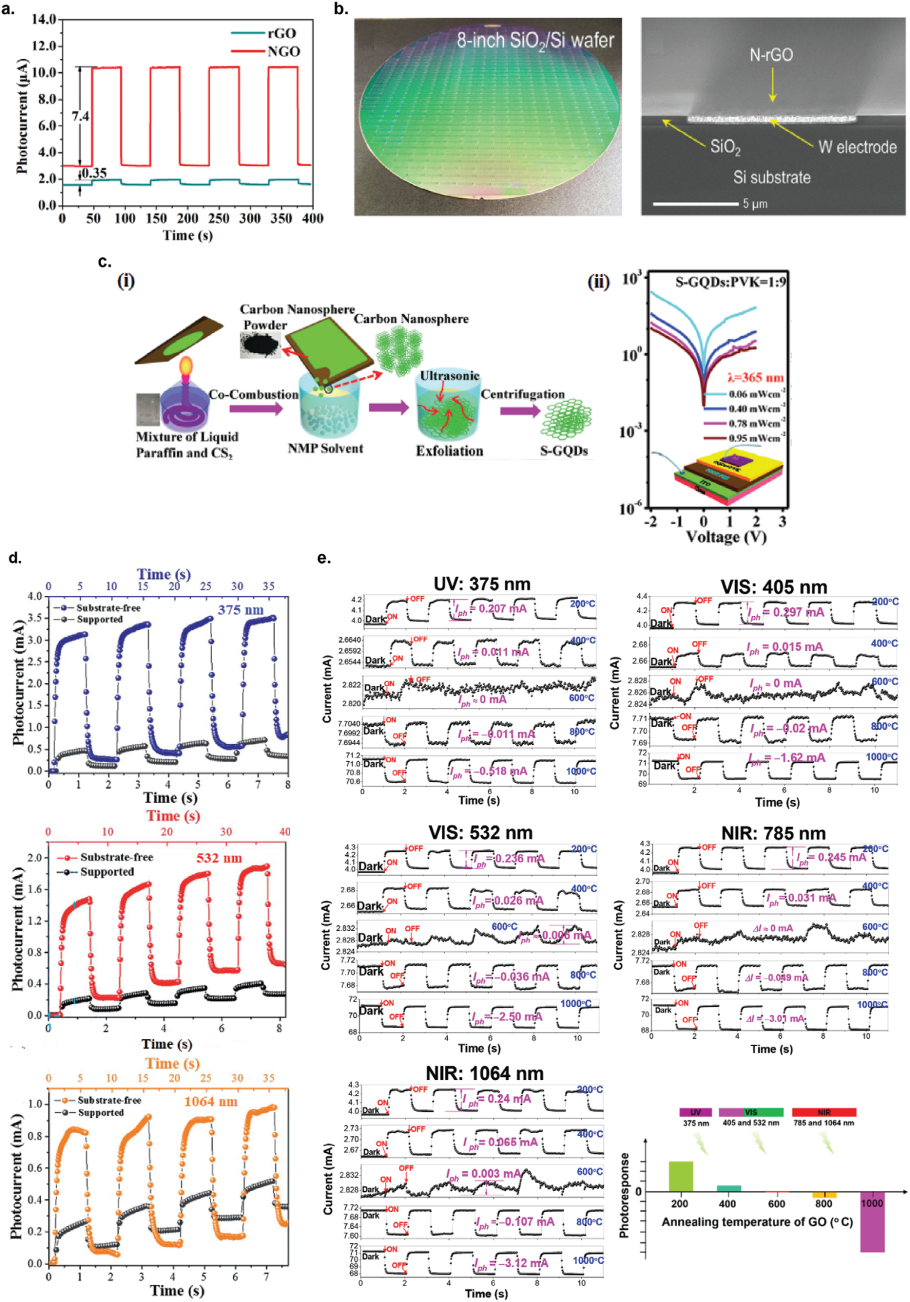
Effect of doping, substrate, and annealing temperature on the photocurrent of graphene‐based photodetectors. a) Photocurrent transients of rGO and N‐rGO films under 3.0 mW cm^−2^ white‐light illumination. Reproduced with permission.^[^ ^85^ ^]^ Copyright 2015, Elsevier Ltd. b) Photograph and the cross‐sectional FESEM images of the N‐rGO photodetector devices on an 8 in. oxide silicon wafer. Reproduced with permission.^[^ ^73^ ^]^ Copyright 2019, American Chemical Society. c) Sulphur‐doped graphene QD (S‐GQD) photodetector for ultraviolet detection. i) Schematic of the synthesis of S‐GQDs. ii) Photoresponsivity of the S‐GQD/poly(9‐vinylcarbazole) (PVK) hybrid photodetector. Inset: The schematic diagram of the PVK/S‐GQD hybrid UV photodetector. Reproduced with permission.^[^ ^132^ ^]^ Copyright 2017, Royal Society of Chemistry. d) Photoresponse transients of the substrate‐free rGO photodetectors under 375, 532, and 1064 nm illumination. Reproduced with permission.^[^ ^74^ ^]^ Copyright 2017, Royal Society of Chemistry. e) Photocurrent transients of suspended rGO photodetectors after subjected to varying annealing temperatures under laser illumination of 375, 405, 532, 785, and 1064 nm at a bias voltage of 0.5 V. Reproduced with permission.^[^ ^75^ ^]^ Copyright 2017, American Chemical Society.

He et al. synthesized N‐doped graphene oxide (N‐ rGO) by a photochemical method for simultaneously realize N‐doping and reduction of GO film in NH_3_ atmosphere at low temperature.^[^ ^85^ ^]^ Compared to the rGO thin film, N‐rGO film exhibits significantly enhanced photoconductivity up to 20 times and showed a faster photoresponse (**Figure** **7**a). Since N‐rGO and rGO have similar oxygen content according to XPS measurement, the photocurrent enhancement and faster photoresponse might be due to the extent of nitrogen doping. It is known that the amino‐like N doping at the basal plane and the pyridine‐like doping at vacancy sites are electron donating and can be excited by light illumination to produce enhanced photocurrent.^[^ ^85^ ^]^ Mohammad Hanif et al. first reported a large‐scale fabrication of N‐ rGO photodetector on an 8 in. SiO_2_/Si wafer by in situ plasma treatment of the spin‐coated GO film in an acetylene‐ammonia atmosphere to produce n‐type semiconductor with substantial quaternary‐N substitution in the rGO lattice (Figure 7b).^[^ ^73^ ^]^ The introduction of plasma in the acetylene‐ammonia atmosphere simultaneously reduces and n‐dopes the GO film with enhanced quaternary‐N substitution. Quaternary‐N substitution results in better n‐doping and carrier mobility enhancement in rGO film compared to other N‐substitution species.^[^ ^73^ ^]^

Gao et al. synthesized sulfur doped GQD (S‐GQD) via the co‐combustion of liquid mixture of paraffin and carbon disulfide (CS_2_) in a low‐cost alcohol lamp (Figure 7c(i)).^[^ ^132^ ^]^ Although the undoped GQD already has a large bandgap at absorption maximum at ≈280 nm (DUV),^[^ ^130^ ^]^ the addition of sulfur doping enhances the absorption in the DUV region. Vertical junction photodetector based on S‐GQD was fabricated, which exhibited photoresponsivity of 307 A W^−1^ under a light intensity of 0.06 mW cm^−2^ at 365 nm (Figure 7c(ii)).

##### Effect of Substrate on the Photocurrent

Partially or fully suspending the monolayer or multilayer graphene sheet has been shown to increase photocurrent by fourfold^[^ ^349^ ^]^ or even tenfold.^[^ ^350^ ^]^ Removing the substrate from the active channel eliminates the carrier scattering due to the interaction with the substrate's surface polar phonons and charge impurity, thus increasing the mobility of the photoexcited carriers. Carrier cooling through heat dissipation via the substrate also causes the carrier to lose mobility. Tian et al. successfully fabricated fully suspended solution‐processed rGO photodetector.^[^ ^74^ ^]^ The removal of the substrate enhanced the photoresponse by up to ≈6 times compared to the supported rGO photodetector (Figure 7d). The responsivities increased from 53/65.3/29 mA W^−1^ to 330/428/96 mA W^−1^ for illumination at 375/532/1064 nm, for the supported and suspended rGO. Cao et al. fabricated a series of fully‐suspended rGO photodetectors annealed at various temperatures (200–1000 °C) and discovered that the suspended‐rGO photodetectors exhibited 1–4 orders of magnitude faster photoresponse time compared to supported rGO photodetectors under visible and near‐irrared irradiation.^[^ ^75^ ^]^ Similarly, Wen et al. also discovered that the response time of the suspended rGO photodetector was below 50 ms, one to three orders of magnitude faster than the supported rGO photodetectors.^[^ ^351^ ^]^ It was theorized that the absence of heat transfer between the rGO and the substrate in the suspended device accelerates the photocurrent to reach the steady state. Cao et al. compared the THz responsivities of rGO photodetectors between suspended and substrate‐supported device, and the suspended device showed a fourfold increase in responsivity and at least one order of magnitude increase in response speed compared to the substrate‐supported device.^[^ ^84^ ^]^

##### Effect of Annealing Temperature on the Photocurrent

The temperature of annealing during thermal reduction of rGO film has a tremendous effect on the photoresponse behavior of the photodetector. Cao et al. investigated the photoresponse behavior of a suspended rGO photodetector annealed at different temperature (from 200 to 1000 °C) and observed that annealing at temperatures below 600 °C resulted in positive photocurrent, while annealing above 600 °C resulted in negative photocurrent (Figure 7e).^[^ ^75^ ^]^ The author argued that this might originate from the competition between the positive response from the rGO and the negative photoconductivity from Au interdigitated electrode. This finding that the photocurrent can be precisely controlled to be positive or negative by the thermal annealing temperature of rGO opens up possibilities for the development of rGO ‐based light triggered logic devices. Wen et al. fabricated a self‐powered ultra‐broadband photodetector based on free‐standing rGO annealed at different temperatures (200–1000 °C).^[^ ^351^ ^]^ Under ultraviolet (375 nm) to terahertz (118.8 mm) illumination, increasing the annealing temperature was shown to decrease the responsivities of the rGO‐based photodetector, which is due to the differences in the content of oxygen functional groups in the rGO.

#### 6.1.2 Tuning the Detection Wavelength of Graphene‐Based Photodetectors beyond Infrared Region

In this section, we introduce variety of engineering strategies to tune the detection wavelength of graphene‐based photodetectors beyond the infrared region. Wavelengths ranging from mid‐infrared, terahertz, and microwave will be discussed.

##### Mid‐Infrared Photodetection

**Figure 8:**
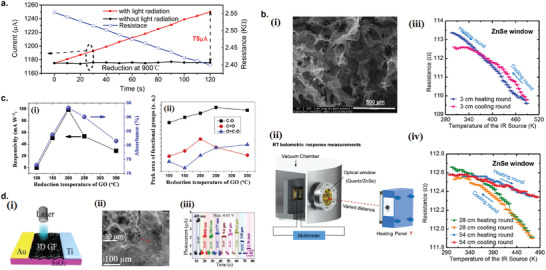
Graphene‐based photodetectors for detection of wavelengths above mid‐IR. a) Illumination time‐dependent current and resistance of rGO films annealed at 900 °C. Reproduced with permission.^[^ ^82^ ^]^ Copyright 2014, AIP. b) Graphene aerogel‐based bolometer for ultrasensitive sensing from ultraviolet to far‐infrared. i) Structure of the partly reduced graphene aerogel (PRGA) film under SEM. ii) Experimental setup for bolometric response measurement. iii,iv) The electrical resistance of the PRGA film versus the surface temperature of the IR source through the ZnSe window with varying distances. Reproduced with permission.^[^ ^143^ ^]^ Copyright 2019, American Chemical Society. c) rGO photodetector for terahertz detection. i) Responsivity and absorbance as a function of reduction temperature of the rGO film under 2.52 THz laser illumination. ii) Peak areas of the oxygen‐containing functional groups versus the reduction temperature. Reproduced with permission.^[^ ^84^ ^]^ Copyright 2018, Elsevier Ltd. d) 3D graphene foam (3D‐GF) photodetector for broadband detection from ultraviolet to microwave light. i) Schematic structure of the 3D‐GF photodetector. ii) SEM images of the 3D‐GF film. iii) Photocurrent under multiple wavelengths from 405 nm to 1.36 mm under 0.05 V bias voltage. Reproduced with permission.^[^ ^144^ ^]^ Copyright 2020, Chinese Laser Press.

For photodetection in mid‐infrared range (3–8 µm), bolometric effect has been shown to be the dominant mechanism of photocurrent increase in rGO photodetectors.^[^ ^82^, ^293^, ^294^ ^]^ Experiments on the evolution of rGO bandgaps have shown that the bandgap cannot be decreased indefinitely by the reduction process because some of the oxygenous groups cannot be removed even after long thermal reduction process (see Figure 6b(i)).^[^ ^352^ ^]^ Thus, the electrons in rGO cannot be photoexcited by mid‐infrared illumination from valence band to conduction band, but can be thermally excited (bolometric effect) to higher energy level to give increased conductance/decreased resistance. **Figure** **8**a(i) shows the time‐dependent current and resistance change of rGO film while being placed nearby a blackbody object heated at 473.14 K, which emits a blackbody radiation peaked at 6.125 µm.^[^ ^82^ ^]^ Sahatiya et al. demonstrated a very sensitive rGO infrared photodetector that was sensitive to the temperature of human hand.^[^ ^83^ ^]^ When a human hand was brought closer to the photodetector, the current increases by ≈10% and when away, the current decreases and the responses were reproducible for many cycles. It is theorized that electrons captured in residual oxygen groups and defect states are easily thermally excited into free electrons due to small energy difference between the bound and free states.^[^ ^82^ ^]^ As the thermal conductivity of rGO is only up to ≈1% of pristine graphene,^[^ ^353^ ^]^ bolometric effect could play a significant role in mid‐infrared detection of rGO photodetectors compared to pristine graphene. Many studies have shown that rGO is a good IR absorber, has a strong temperature dependent electrical resistance and large thermal resistance. Therefore, this materials is a good candidate for bolometric applications.^[^ ^295^, ^354^ ^]^

*Graphene Aerogel‐Based Bolometer*: An extraordinary bolometric response has been demonstrated by a free‐standing PRGA devised by Xie et al.,^[^ ^143^ ^]^ which is an interconnected, porous 3D framework composed of randomly stacked rGO nanosheets (Figure 8b). The high performance of PRGA is attributed to its extremely low thermal conductivity, high porosity, very low density, and very tunable bandgap due to abundant functional groups. Bolometers need to have the following three properties to achieve high sensitivity: very high IR absorption, high temperature coefficient of resistance (TCR), and small thermal conductance. On top of that, to achieve fast response, the bolometer's heat capacitance needs to be as small as possible. PRGA has a wideband photon absorption due to the excellent photon absorption properties of graphene and GO, has a very high TCR due to abundant functional groups, ultralow thermal conductance (6–0.6 mW m^−1^ K^−1^ from 295 to 10 K), and has a very small volumetric heat capacitance due to ultralow density from high porosity. These combinations of beneficial properties have enabled PRGA to be an ultra‐sensitive IR bolometric detector. The PRGA film could detect a temperature change of 0.2, 1, and 3 K of a target at 3, 25, and 54 cm distance. At room temperature, it could even detect 1550 nm laser at a power as low as 5.9 µW.^[^ ^143^ ^]^

##### Terahertz Photodetection

Cao et al. fabricated a fully suspended rGO photodetector that could detect terahertz (THz) signal with the responsivity adjustable over a wide range from 10^−2^–10^2^ mA W^−1^ by tuning the thermal reduction temperature of GO film that resulted in different reduction degree of the rGO film.^[^ ^84^ ^]^ The responsivity for THz signal at 118.8 µm peaked at 98.71 mA W^−1^ at thermal reduction temperature of 200 °C (Figure 8c(i)). The variation of responsivity as a function of reduction temperature coincided with the ratio of the peak area for C=O functional group (Figure 8c(ii)), and thus the author suggested that the C=O group was the most important functional group for the absorption of THz signal in rGO. The author argued that the absorption of THz waves by the C=O group intensifies the vibration of the group, increasing the temperature of the device. The thermally excited electron–hole pairs then contributed to the increase of the current.^[^ ^84^ ^]^

##### Microwave Photodetection

Li et al. reported the ultra‐broadband photodetector based on 3D graphene foam (3D‐GF) with asymmetric electrodes that can detect wavelengths ranging from ultraviolet to microwave (Figure 8d).^[^ ^144^ ^]^ The 3D‐GF was synthesized by a solvothermal treatment of GO nanosheets dispersed in ethanol. The asymmetric configuration of the photodetector with Au electrode on one side and Ti on the other is important for the enhanced photodetecting performance of microwave signal, due to the combination of the photovoltaic and photothermoelectric (PTE) mechanism. Due to the asymmetric electrode configuration, the local temperature gradient created by the light illumination creates a potential gradient Δ*V* = Δ*S*Δ*T*, where Δ*S* is the net Seeback coefficient difference across the opposite electrodes, which assists in enhancing the photocurrent. In this asymmetric structure, the PTE effect makes 90% contribution to the total photocurrent, while photovoltaic and photoconductive effect makes up only than 10%.^[^ ^144^ ^]^ Therefore, by leveraging this beneficial PTE effect by designing an asymmetric device structure, the photodetector sensitivity could be enhanced up to the microwave region.

#### 6.1.3 Other Engineering Strategies to Improve Photocurrent of Graphene‐Based Photodetectors

##### Laser Scribing of GO for Microscale‐Controlled Reduction

**Figure 9:**
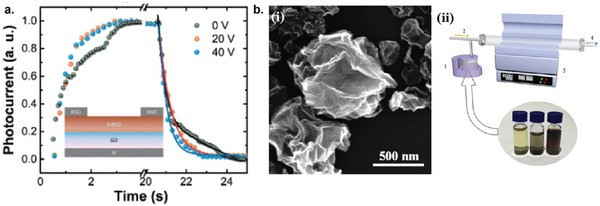
Further engineering to improve the performance of graphene‐based photodetectors. a) Photoresponse of the laser‐scribed rGO/s‐rGO/rGO photodetector structure on top of GO with *V*_g_ = 0 V (black), 20 V (orange), and 40 V (blue). The inset shows a schematic view of the photodetector. Reproduced with permission.^[^ ^76^ ^]^ Copyright 2018, The Japan Society of Applied Physics. b) Crumpled reduced graphene oxide (c‐rGO). i) High magnification SEM image the of c‐rGO. ii) Schematic diagram of the fabrication apparatus of c‐rGO: 1, the ultrasonic atomizer; 2, the carrier gas (N_2_); 3, the tube furnace; and 4, the exhaust gas. Reproduced with permission.^[^ ^77^ ^]^ Copyright 2017, Elsevier Ltd.

Feng et al. demonstrated the use of laser beams to reduce GO film in a more controlled manner on a micrometer scale.^[^ ^76^ ^]^ The C/O ratio of the rGO can be precisely controlled by adjusting the number of current pulses applied to the laser. The semi‐reduced rGO (s‐rGO) with C/O ratio of 1–1.3 was obtained by this method. Compared to rGO which has C/O ratio of 1.84, the s‐rGO film has a much lower dark current than the rGO film, but with conductivity high enough to allow photocurrent generation, thus increasing the photosensitivity of the device. Furthermore, by precisely controlling the laser reduction annealing only to the upper part of the film, a monolithic field‐effect transistor (FET) structure of rGO/s‐rGO/rGO on top of GO/aluminum for infrared photodetection was fabricated (**Figure** **9**a). The dark current could be further modulated by applying the gate bias to reach a very small value of 1 nA. The responsivity obtained under illumination at 1550 nm was 0.18 A W^−1^.^[^ ^76^ ^]^

##### Crumpled Reduced Graphene Oxide

Higher light absorption by graphene can be achieved by optimizing the morphology of the active graphene layer. An order‐of‐magnitude enhancement of the optical extinction of graphene has been achieved by increasing graphene's areal density by buckled 3D structure.^[^ ^355^ ^]^ Gao et al. demonstrated the preparation of crumpled reduced graphene oxide (c‐rGO) by a simple ultrasonic pyrolysis method for photodetecting application (Figure 9b(i)).^[^ ^77^ ^]^ Once formed into uniform film, c‐rGO is thought to scatter and absorb more incoming light, hence boosting the photocurrent. Further photocurrent enhancement was obtained by incorporating ZnO and PbS nanoparticles with the c‐rGO, demonstrating tenfold increase in the photocurrent. Figure 9b(ii) shows the schematic diagram of the ultrasonic pyrolysis apparatus to obtain c‐ rGO. The detailed steps of the c‐rGO preparation are as follows (labelled in Figure 9b(ii)): 1) prefabricated GO alcohol solution was poured into an ultrasonic atomizer; 2) the nebulized GO aerosol droplets were carried by nitrogen gas from a steel tube; 3) the droplets were carried through a preheated tube furnace at 550 °C. The GO aerosol droplets gradually evolved into c‐rGO and deposited onto the substrate at the cold rear part of the furnace; and 4) discharge of exhaust gas.

#### 6.1.4 Graphene‐Based Hybrid Photodetectors

Introducing photosensitive nanostructures such as quantum dots, nanorods, nanowires, other 2D materials, or photosensitive polymers as hybrids with graphene materials has the potential to improve the photodetector performance in terms of photosensitivity, photoresponse speed, or tuning the photoresponse at specific wavelength, by combining the superior light absorption capability of the photosensitive materials and the ultrahigh charge mobility of graphene. Here we summarized recent developments in photodetectors based on liquid‐exfoliated graphene hybridized with variety of photosensitive materials.

##### Graphene/Metal Quantum Dot

Photodetectors hybridized with plasmonic structures such as metal quantum dots have been proposed recently as an effective method to increase the light absorption and hence improve the photoresponse.^[^ ^356^, ^357^ ^]^ Oscillation of conducting electrons at the interface between metal and other material with different permittivity gives rise to surface plasmon resonance (SPR) that enhances light absorption. Radoi et al. reported a very large spectral bandwidth photodetection that encompasses ultraviolet, visible, and near infrared of solution‐processed graphene photodetectors decorated with either gold, siver QDs, or gold QDs encapsulated with bovine serum albumin.^[^ ^86^ ^]^ Photodetectors made from Ag‐functionalized graphene inks showed significantly higher responsivities compared to bare graphene or Au‐functionalized graphene. It was argued that the enhancement of the photoresponsivities for Ag‐functionalized compared to Au‐functionalized graphene photodetector was because Ag quantum dots have much higher optical extinction (absorption + scattering) compared to Au quantum dots. Moreover, for the diameter of Ag quantum dots used in the experiment, the scattering part dominates the absorption, enhancing the electromagnetic field amplitude over significant distance.^[^ ^86^ ^]^ Later on, Kumar et al. demonstrated the synthesis of graphene photodetector hybridized with Cu–Ni bimetallic QDs by in situ synthesis.^[^ ^87^ ^]^ Bimetallic composites often possesses very exciting physical as well as chemical properties compared to single metallic component, which might improve light absorption for better photodetecting performance.^[^ ^358^ ^]^ Fabrication of rGO/Pt QDs photodetector via layer‐by‐layer (LbL) assembly of oppositely charged rGO nanosheets and charged Pt QDs was also demonstrated by Zhu et al. and the MIR photoresponse of the device was evaluated.^[^ ^88^ ^]^ Compared to bare rGO film, the rGO/Pt QD film exhibited 50% increase in the photocurrent under MIR illumination.

**Figure 10:**
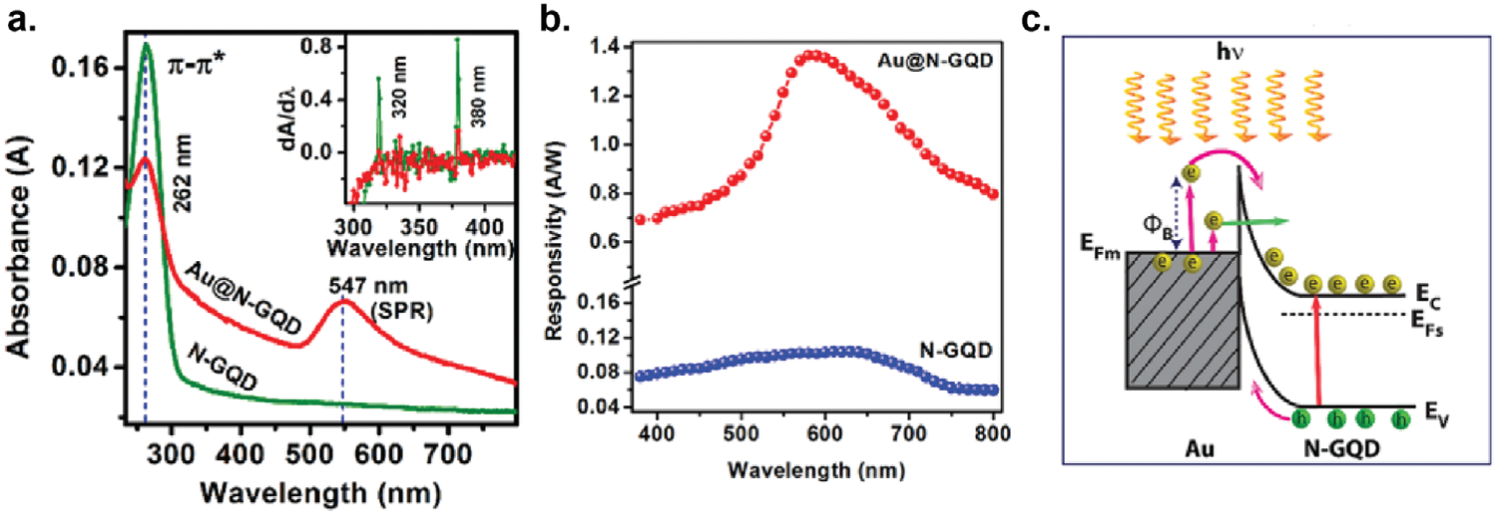
N‐doped GQD photodetector hybridized with Au nanoparticles. a) UV–vis absorption spectra of N‐GQDs and Au/N‐GQDs. The vertical dashed lines show the absorption peaks corresponding to N‐GQDs (262 nm) and plasmonics Au NPs (547 nm). b) Responsivities of of N‐GQDs and Au/N‐GQDs photodetector under 5 V bias. c) Schematic illustration of the band diagram and the electron transfer in Au/N‐GQDs under light illumination. Reproduced with permission.^[^ ^133^ ^]^ Copyright 2020, American Chemical Society.

Graphene quantum dots (GQDs) decorated with plasmonic nanoparticles such as metal QDs could significantly alter the optoelectronic properties of the GQDs, resulting in exceptional properties. Das et al. prepared nitrogen‐doped GQDs (N‐GQDs) hybridized with gold quantum dots (Au QDs) by a one‐step green reduction process and utilized them as a photodetector with a high photoresponsivity (≈1.36 A W^−1^) and showed ≈10^4^ faster response time compared to bare N‐GQD photodetector (≈0.103 A W^−1^).^[^ ^133^ ^]^ The absorption spectra of the composite N‐GQD/Au QD structure showed additional absorption peak due to SPR absorption from Au QDs at ≈547 nm and resulted in overall enhancement of light absorption across UV–vis–NIR spectrum (**Figure** **10**a). The photoresponsity peak of N‐GQD/Au QD device near its plasmonic absorption peak (≈547 nm) is a strong indication that the enhanced photoresponse is attributed to the plasmon resonance‐enhanced absorption and subsequent hot electron generation (Figure 10b). Figure 10c shows the band diagram schematic of photoexcited charge transfer during illumination from Au QD to GQD via hot carrier injection and tunneling mechanism.

##### Graphene/Semiconductor

**Figure 11:**
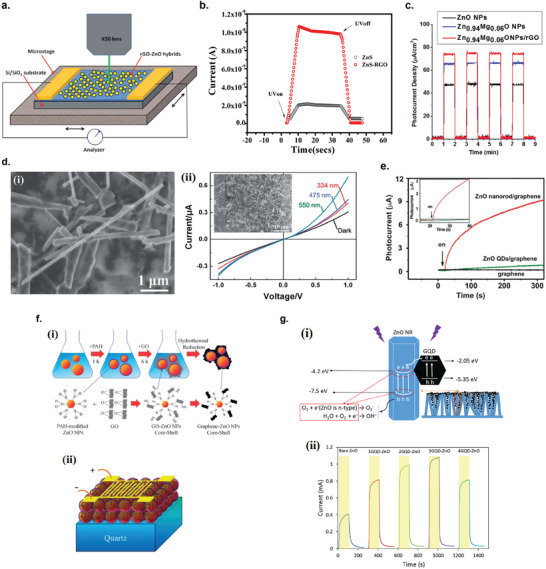
Hybrid graphene/Zn‐based nanoparticle photodetectors. a) Schematic of the hybrid ZnO QD/rGO photodetector and the in situ measurement setup. Reproduced with permission.^[^ ^101^ ^]^ Copyright 2012, Royal Society of Chemistry. b) Photoresponse transients of ZnS and ZnS/rGO photodetectors under on/off UV light irradiation. Reproduced with permission.^[^ ^111^ ^]^ Copyright 2009, American Chemical Society. c) Photocurrent response of ZnO, Zn_0.94_Mg_0.06_O NPs, and Zn_0.94_Mg_0.06_O/rGO nanocomposites under a xenon lamp. Reproduced with permission.^[^ ^113^ ^]^ Copyright 2016, Elsevier Ltd. d) rGO/C‐doped ZnO NW photodetector. i) SEM image of the C‐doped ZnO/rGO hybrid nanocomposites. ii) Current–voltage characteristics of the device under different illumination conditions. Reproduced with permission.^[^ ^114^ ^]^ Copyright 2014, Royal Society of Chemistry. e) Background‐subtracted photocurrent transients of pure rGO, ZnO QD/rGO hybrid and the ZnO nanorod/rGO hybrid photodetectors at 1.084 mW cm^−2^ 370 nm radiation with bias of 5 V. Inset: an enlarged view of the exact starting point when light is on. Reproduced with permission.^[^ ^115^ ^]^ Copyright 2011, Royal Society of Chemistry. f) ZnO nanoparticle–rGO core–shell photodetector. i) Schematic of the fabrication process for ZnO NP/rGO core–shell structures. ii) Schematic of the rGO/ZnO NP core–shell UV photodetector. Reproduced with permission.^[^ ^117^ ^]^ Copyright 2013, Royal Society of Chemistry. g) GQD/ZnO NR photodetector. i) Energy band diagram of the GQD/ZnO NR composite and its carrier transport mechanism at the interfacial region under UV irradiation. ii) Photocurrent transients of bare ZnO and nGQD/ZnO NR photodetectors under UV irradiation. Reproduced with permission.^[^ ^134^ ^]^ Copyright 2017, Elsevier Ltd.

Graphene/Zn‐Based Nanoparticles: ZnO, a wide‐bandgap semiconductor, has drawn a great deal of attention due to its unique optical electrical properties including a wide bandgap (3.37 eV) and strong resistance to high‐energy photon irradiation.^[^ ^359^ ^]^ However, UV photodetectors based solely on ZnO suffered very slow response time due to adsorption and desorption of oxygen on ZnO surface.^[^ ^360^ ^]^ Liu et al. reported a method to fabricate rGO/ZnO QDs by a simple hydrothermal process without using any surfactant.^[^ ^89^ ^]^ Because the ZnO QDs are anchored to rGO without any linkers, the interfacial contact between the QDs and rGO was much improved. Later, they developed a method to prepare rGO/ZnO QD nanocomposites by an ultralow‐temperature solvothermal process in which the GO was reduced at low temperature (<170 °C).^[^ ^90^ ^]^ Zhan et al. reported the fabrication of a visible light photodetector based on rGO/ZnO QDs hybrid structure (**Figure** **11**a).^[^ ^101^ ^]^ The thermal treatment process at the elevated temperature during fabrication not only reduces the GO into rGO but also simultaneously dopes the ZnO QDs with carbon atoms, enabling the visible‐light photodetection. The doping process introduces new energy levels within the ZnO gap, narrowing the bandgap, thus extending the absorption range from UV zone to the visible zone. Like ZnO, ZnS, a wide bandgap semiconductor (≈3.7 eV), also has the capacity to work as efficient UV photodetectors.^[^ ^361^ ^]^ Roy et al. fabricated rGO/ZnS QD photodetector which exhibited on/off ratio of ≈1.96 × 10^2^ under UV irradiation, which was a significant improvement compared to pure ZnS QD photodetector with on/off ratio of only ≈1.19 × 10^1^ (Figure 11b).^[^ ^111^ ^]^ The doping of ZnO QDs for bandgap engineering was also attempted with other oxide semiconductor, such as CdO by sol–gel synthesis method, which resulted in Zn_(1−_ *_x_*_)_Cd*_x_*O (*x* = 0.02) QDs used to decorate rGO for improved UV light detection.^[^ ^112^ ^]^ Doping ZnO QDs with MgO is also a good option since the ionic radii of Mg^2+^ (0.66 Å) and Zn^2+^ (0.74 Å) are relatively similar, thus keeping the crystalline structure and lattice parameter unchanged.^[^ ^362^ ^]^ Kharatzadeh et al. reported the fabrication of rGO/ Zn_(1−_ *_x_*_)_Mg*_x_*O (*x* = 0.02, 0.04, and 0.06) QD photodetector in which the photoresponse in the UV region can be controlled by the amount of MgO doping, due to bandgap modulation of the QDs.^[^ ^113^ ^]^ Under UV light illumination, the photocurrent intensity increased from pure ZnO QD photodetector to Zn_0.94_Mg_0.06_O QD photodetector, and further increased for rGO/ Zn_0.94_Mg_0.06_O QD photodetector (Figure 11c).

Liu et al. fabricated rGO/carbon (C)‐doped ZnO nanowire (NW) nanocomposites by ultrasonication mixing of rGO with C‐doped ZnO NW in isopropanol (Figure 11d(i)).^[^ ^114^ ^]^ C‐doped ZnO NWs were synthesized on carbon cloth by carbothermal evaporation method and then the central carbon fiber was removed by annealing at 800 °C. Figure 11d(ii) shows the typical *I*–*V* characteristics of the hybrid photodetector under dark and illumination at 314, 475, and 550 nm. Since pristine ZnO has a bandgap of 3.37 eV and only responds to UV light,^[^ ^363^ ^]^ the photoresponse in the visible region is due to C‐doping of ZnO which extends the photoresponse into visible region.

Chang et al. demonstrated the first time the development of visible‐blind, UV photodetectors based on rGO/ZnO nanorod (NR) which was synthesized via a facile in situ aqueous seeded growth method.^[^ ^115^ ^]^ In this hybrid structure, ZnO NR function as UV absorbing and charged carrier generating materials, while rGO functions as charge transporting, highly conductive channel. The ZnO NRs were synthesized by in situ growth of ZnO NRs from ZnO QD seeds by immersing in zinc nitrate solution. The photoresponsivity of the hybrid device under UV irradiation reached 22.7 A W^−1^, over 45 000 times higher than single‐layer graphene device (≈0.1–0.5 mA W^−1^).^[^ ^331^ ^]^ As a control, the photoresponse of rGO/ZnO QD and pure rGO photodetectors were also measured, and the photoresponsivity only reached 0.35 and ≈0 A W^−1^, respectively (Figure 11e). The origin of better photoresponsivity of rGO/ZnO NR device compared to rGO/ZnO QD and pure rGO is better connection between ZnO nanorods and rGO due to in situ hydrothermal growth, compared to connection between ZnO QDs and rGO which was formed by simple mixing.

Despite excellent performance of ZnO NR hybrid devices, there are large number of surface states and defects on ZnO, which can act as trap centers that could limit the sensitivity to UV light.^[^ ^360^ ^]^ By applying surface modification to the ZnO NR with 3‐aminopropyl triethoxysilane (APTES), the sensitivity of the rGO/ZnO NR photodetector to UV light could be increased.^[^ ^116^ ^]^ The origin of better UV sensitivity is due to better interfacial contact between the surface‐modified ZnO NR (ZnO(H)) and rGO, due to opposite surface charges, which improves the photogenerated charge transfer efficiency.

Shao et al. fabricated a high‐performance UV photodetector by wrapping graphene onto ZnO QDs to form ZnO/graphene core–shell structure.^[^ ^117^ ^]^ The core–shell structure was synthesized by surface modification of ZnO QDs with amine groups, coating of GO onto the QDS, and finally converting the GO into graphene by hydrothermal reduction process (Figure 11f(i)). Figure 11f(ii) shows the schematic illustration of the fabricated UV photodetector. The responsivity of the device under 375 nm illumination was 640 A W^−1^ at 20 V bias. This method of wrapping graphene onto ZnO QDs can also be generally applied to various metal oxide nanoparticles, such as In_2_O_3_ and porous Co_2_O_3_ for variety of applications.

Graphene quantum dots (GQD) have unique properties inherited from graphene sheets, but compared to graphene, they have additional advantages: 1) they have higher penetrability into pores and small spaces due to their remarkably smaller size, therefore improving charge transfer; and 2) their semiconducting nature gives them lower conductivity than graphene, increasing the light sensitivity due to lower dark current. Rahimi et al. prepared thin films of almost vertically‐grown ZnO NRs via a solvothermal method and hybridized them with GQDs synthesized by pyrolyzing citric acid (Figure 11g(i)).^[^ ^134^ ^]^ The dip‐coating method was employed to coat the synthesized GQD solution onto the ZnO NR thin film. The number of dip‐coating process greatly determined the photocurrent of the hybrid device under UV (365 nm) irradiation, where *n* = 3 resulted in the highest photocurrent, which is ≈2.75 times higher than pure ZnO NR device (Figure 11g(ii)). Further dip‐coating would result in decreased photocurrent because less UV light would reach to the ZnO NRs.

**Figure 12:**
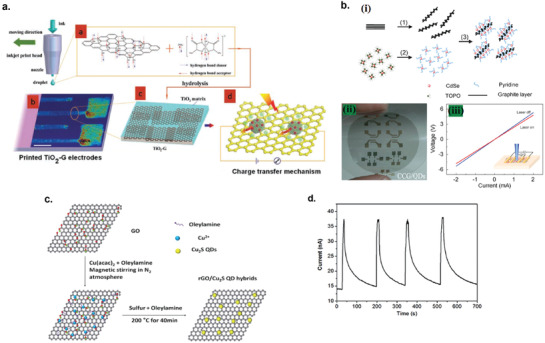
Graphene/semiconductor hybrid photodetectors. a) Schematic of inkjet printing of rGO/TiO_2_ composite for photodetector fabrication. Reproduced with permission.^[^ ^91^ ^]^ Copyright 2010, WILEY‐VCH. b) rGO/CdSe QD photodetector. i) Schematic illustration of the formation of rGO/CdSe QD composites. ii) rGO/CdSe QD photodetector arrays with patterned electrodes on a flexible and transparent plastic substrate. iii) Current–voltage characteristics measured by a four‐probe method of the hybrid photodetector on a flexible substrate with and without laser irradiation. Reproduced with permission.^[^ ^95^ ^]^ Copyright 2010, WILEY‐VCH. c) Schematic of the synthesis procedure of the rGO/Cu_2_S QD hybrids using a hot‐injection technique. Reproduced with permission.^[^ ^102^ ^]^ Copyright 2013, Royal Society of Chemistry. d) The photocurrent transients of rGO/InP QD photodetector under intermittent blue light irradiation with a voltage of 1 V. Reproduced with permission.^[^ ^103^ ^]^ Copyright 2016, Royal Society of Chemistry.

*Graphene/TiO_2_*: Manga et al. developed a solution‐processable method to fabricate a hybrid rGO/TiO_2_ QD hybrid photodetector via an inkjet printing of the precursor solution of GO and titanium (IV) bis(ammonium lactate) dihydroxide.^[^ ^91^ ^]^ This technique was based on a direct conversion of TiO_2_ precursor source in GO matrix (**Figure** **12**a). The fabricated device was then subjected to reduction by keeping in hydrazine vapor overnight and annealed in vacuum. Compared to the previous method of combining the already pre‐formed TiO_2_ QDs with rGO, this technique has the advantage of producing an extended heterojunction of TiO_2_ and graphene which increases the effectiveness and multiplication of charge transfer at the interface due to shorter collection path. Variations of the TiO_2_ QD hybridization method to rGO have also been reported elsewhere, such as a combination of layer‐by‐layer (LbL) assembly and surface sol–gel process,^[^ ^92^ ^]^ CVD deposition of TiO_2_ nanoparticles onto the rGO layer,^[^ ^93^ ^]^ and directly spray‐coating the rGO/TiO_2_ solution onto gold interdigitated electrodes.^[^ ^94^ ^]^

*Graphene/CdSe*: Geng et al. fabricated a photoconductor based on the composites of rGO and CdSe QDs via *π*–*π* stacking of aromatic structures between rGO and CdSe QDs capped with pyridine (Py).^[^ ^95^ ^]^ The schematic of the composite formation is shown in Figure 12b(i). The composite was formed by mixing the diluted rGO solution with Py‐capped CdSe QDs in aqueous solution. The application of Py as the capping layer is not only to introduce *π*–*π* stacking between rGO nanosheets and the QDs, but the small Py molecules should also facilitate charge transfer between the two components. The vacuum‐filtered rGO/CdSe QD composite was transferred to plastic by mechanical pressing to fabricate a photoconductor with Au electrodes in 4‐point probes configuration (Figure 12b(ii)). Illuminating the composite film to 473 nm laser irradiation at power of 94.5 mW resulted in the increase of conductance up to ≈10.5% (Figure 12b(iii)). Later on, Lin et al. prepared rGO/CdSe QD nanocomposites by directly adding rGO into the reaction solution during the process of CdSe QD synthesis, thereby the CdSe QD were directly anchored to the rGO nanosheets, without any intermediate molecules that might limit the efficiency of the charge transfer between the two components.^[^ ^96^ ^]^ The separation and charge transfer between the two component was much more efficient compared to simply physically mixing rGO and CdSe QD, which resulted in a dramatically enhanced photoresponse with very fast response time. Yu et al. demonstrated the use of catalyst‐free CVD method to deposit CdSe QDs on rGO without any linkers.^[^ ^97^ ^]^ It was discovered that the response time of the rGO/CdSe QD photodetector could be modulated by the coverage level of CdSe QD on the rGO, where low coverage resulted in highly variable response time depending on the different gas exposures during measurement. When the coverage was high, the response time was three orders of magnitude faster and much less affected by the gas exposures. When the QD coverage was low, the gas molecules could easily adsorb to the rGO layer and might act as trap states, and greatly modulated the photocurrent dynamics, while high coverage of the QDs would lessen the adsorption of gas molecules and at the same time enhances the photoresponse because more light could be harvested by the photoexcitation and charge transfer from CdSe QDs to the rGO layer.

*Graphene/PbS*: Lead sulfide (PbS) is a binary semiconductor from IV–VI family with a narrow bandgap of ≈0.41 eV, making it a promising material for infrared photodetector.^[^ ^364^ ^]^ An NIR rGO/PbS QD composite photodetector was reported by Ghosh et al. fabricated by a simple one‐step synthesis solvothermal method, where the reduction of graphene oxide, synthesis of PbS QDs and decoration of the QDs on rGO was done simultaneously.^[^ ^98^ ^]^ The responsivity was estimated to be 9.3 × 10^−3^ A W^−1^ under NIR illumination (808 nm) at 6.72 mW mm^−1^. Effect of annealing temperature on NIR photodetecting performance of rGO/PbS QD photodetector was investigated by Yousefi et al. and discovered that the responsivity is maximized when the device was annealed at 300 °C in H_2_/Ar gas atmosphere.^[^ ^99^ ^]^ A vertical structure of rGO/PbS QDs field‐effect phototransistor with Au/Ag nanowire bottom source electrode and Au top drain electrode was fabricated by Song et al.^[^ ^100^ ^]^ The vertical structure allowed for short channel length (250 nm) which boosts the photocurrent due to reduced transit time of the photoexcited carriers in the channel. Thanks to this, the photoresponsivity of the device reached 2 × 10^3^ A W^−1^ and specific detectivity of 7 × 10^12^ Jones was obtained under 808 nm laser illumination.

*Graphene/Cu_2_S*: Cu_2_S is a well‐known p‐type semiconductor with a bulk bandgap of 1.2 eV, making it an ideal material for sunlight absorbing materials for solar cell applications.^[^ ^365^ ^]^ Su et al. fabricated an rGO/Cu_2_S QDs hybrid photodetector obtained by one‐pot synthesis, wherein the reduction of GO and the growth of Cu_2_S QDs occur in situ simultaneously (Figure 12c).^[^ ^102^ ^]^

*Graphene/InP*: Similarly, InP with its direct bandgap of 1.35 eV is also a promising material for photodetecting applications when hybridized with rGO, as was demonstrated by Jiang et al.^[^ ^103^ ^]^ Upon illumination with blue light (405 nm, 80 mW cm^−2^), the photocurrent exhibited fast photoresponse, and although the decaying time was rather slow after the light was turned off, repeatable photoresponse was demonstrated over repeated on‐and‐off cycles (Figure 12d).

*Graphene/Bi_2_S_3_*: Bismuth sulfide Bi_2_S_3_ is an important semiconductor of the V–VI group which has a great potential for photodetecting applications, due to its direct bandgap of ≈1.3 eV, high absorption coefficient, nontoxicity, and abundance of its raw materials on earth.^[^ ^366^ ^]^ Heshmatynezhad et al. fabricated rGO/Bi_2_S_3_ QD photodetector synthesized by UV‐assisted sonication method.^[^ ^104^ ^]^ The effect of UV‐sonication irradiation time, calcination temperature and rGO concentration during the synthesis process was investigated on the electrical and photoresponse of the hybrid devices. Increasing the calcination temperature decreased the magnitude of photocurrent, from 9.13 mA cm^−2^ at calcination temperature of 200 °C to only 0.18 mA cm^−2^ at calcination temperature of 300 °C. Furthermore, increasing the calcination temperature increases the photoresponse time of the device and increasing the concentration of rGO seemed to decrease the response time.

*Graphene/WO_3_*: WO_3_ is an n‐type semiconductor material that has attracted significant research interest due to its exciting physical and chemical properties,^[^ ^367^ ^]^ and has potential for UV detection due to its indirect large energy bandgap (3.3 eV).^[^ ^368^ ^]^ Shao et al. developed an UV photodetector based on rGO/WO_3_ nanodiscs (NDs) hybrid material.^[^ ^105^ ^]^ The composite material was synthesized by a three‐step process: 1) GO/Na_2_WO_4_ precursor was synthesized by a homogeneous precipitation, 2) GO/Na_2_WO_4_ precursor was transformed into GO/H_2_WO_4_ composites by acidification, 3) GO/H_2_WO_4_ composites were reduced to rGO/WO_3_ NDs via hydrothermal reduction process. SEM images show that the average diameter and thickness of the WO_3_ NDs are ≈350 and ≈30 nm, respectively. When WO_3_ NDs are in contact with the rGO, the photogenerated electrons are transferred from the conduction band of WO_3_ to the RGO, and rapidly collected by the electrodes due to high mobility of the rGO. The highest photoresponsitivity achieved was 6.4 A W^−1^ at 347 nm, which was 17 times higher that pure WO_3_ device.

**Figure 13:**
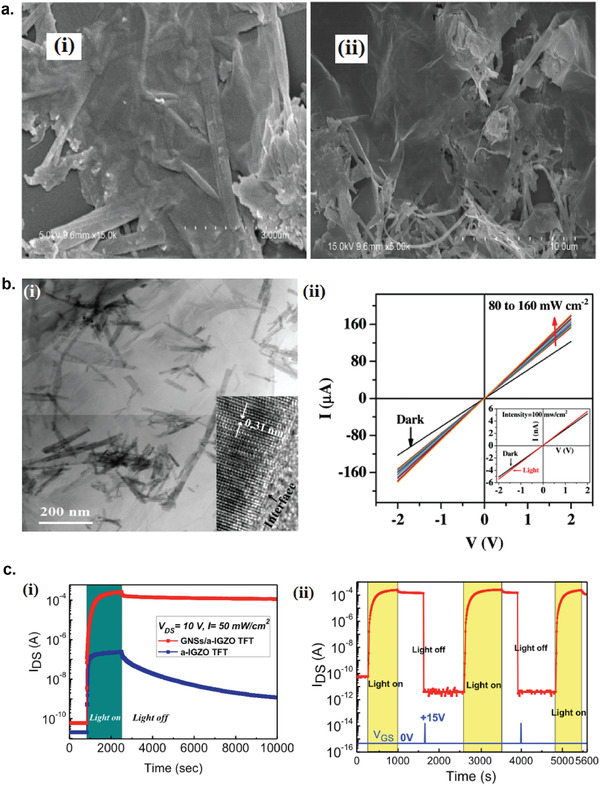
Graphene/semiconductor hybrid photodetectors. a) rGO/HfX_3_(X = Se and S) hybrid photodetectors. SEM images of i) rGO/HfSe_3_ composites and ii) rGO/HfS_3_ composites. Reproduced with permission.^[^ ^106^ ^]^ Copyright 2017, Elsevier Ltd. b) rGO/Cd_1‐_ *_x_*Zn*_x_*S hybrid photodetector. i) TEM image of the rGO/CdZnS composite. Inset: the HRTEM image of the composite. ii) *I*–*V* characteristics of rGO/CdZnS hybrid photodetector. Reproduced with permission.^[^ ^108^ ^]^ Copyright 2017, Springer Nature. c) FLG/IGZO hybrid photodetector. i) Photocurrent transient of a‐IGZO and FLG/a‐IGZO photodetector with zero gate bias and under a light pulse (50 mW cm^−2^). ii) Cycling responses of the optical programming and electrical erasing of the FLg/a‐IGZO photodetector. A short positive gate‐voltage pulse (+15 V, 600 ms) was applied at 1620 and 3910 s to erase the memory effect. Reproduced with permission.^[^ ^137^ ^]^ Copyright 2015, American Chemical Society.

*Graphene/HfX_3_ (X = S, Se)*: HfX_3_ (X = S, Se) are semiconducting materials belonging to the family of trichalcogenides of group IV transition metals, which possess chain‐like and layered‐type structure.^[^ ^369^ ^]^ Compared to other transition metal chalcogenides, the chemical and physical properties of HfX_3_ have rarely been studied except for a few theoretical investigations. Fan et al. fabricated flexible photodetectors based on hybrid rGO/HfX_3_ nanobelts (NBs) on polypropylene film that can respond to visible to near‐infrared radiation (**Figure** **13**a).^[^ ^106^ ^]^ The hybrid photodetectors showed enhanced photoresponsivity compared to bare HfX_3_ devices. The peak photoresponsivities obtained were 0.55 and 0.5 mA W^−1^ for hybrid rGO/HfS_3_ and rGO/HfSe_3_, respectively under 405 nm irradiation, and 7.57 × 10^−3^ and 1.2 × 10^−2^ mA W^−1^ for rGO/HfS_3_ and rGO/HfSe_3_, respectively under 980 nm irradiation.

*Graphene/ Cu_2_SnS_3_*: Cu_2_SnS_3_ (CTS) is an important p‐type semiconductor with a bandgap of 0.93–1.35 eV, and possess many useful properties such as high absorption coefficient (>10^4^ cm^−1^), high hole mobility (80 cm V^−1^ s^−1^), high electrical conductivity (10 Ω^−1^ cm^−1^), and hole concentration (10^18^ cm^−3^).^[^ ^370^ ^]^ Kamalanathan et al. synthesized the composite of rGO and CTS nanorods and microbars by a simple solvothermal route.^[^ ^107^ ^]^ The addition of complexing agent ethylenediaminetetraacetic acid (EDTA) during solvothermal synthesis resulted in CTS nanorod shape, while the absence of the complexing agent resulted in the microbar shape. Therefore, the complexing agent EDTA acted as a controller of the shape and size of the composites. The measured photocurrent of rGO/CTS nanorod composites was 60% higher than rGO/CTS microbar under white light illumination at 100 mW cm^−2^. The higher photocurrent of the nanorod composites might be due to increased surface to volume ratio which increases the photogenerated electron–hole dissociation rate across rGO/CTS interface.

*Graphene/ Cd_1‐x_Zn_x_S*: Cadmium zinc sulfide (Cd_1‐_ *_x_*Zn*_x_*S) tertiary alloy can form a continuous range of solid solutions and allows for tuning of the optical band gap from visible (CdS = 2.42 eV) to ultraviolet (ZnS = 3.7 eV) region. Ibrahim et al. reported the one pot single step solvothermal synthesis of rGO/Cd_0.5_Zn_0.5_S (CdZnS) NR hybrid photodetector, where the reduction of GO and synthesis of CdZnS NRs and their integration to rGO was done simultaneously (Figure 13b(i)).^[^ ^108^ ^]^ The photoresponse of the hybrid device is more than four orders of magnitude higher compared to bare CdZnS photodetector, emphasizing the importance of rGO channel for efficient photogenerated charge transfer and collection (Figure 13b(ii)).

*Graphene/Indium‐Gallium‐Zinc‐Oxide (IGZO)*: Amorphous oxide semiconductors, such as amorphous indium‐gallium‐zinc‐oxide (a‐IGZO), have drawn considerable research attention for next‐generation flat, flexible, and transparent display devices, due to their high electron mobility, optical transparency, chemical stability, and processing versatility.^[^ ^371^ ^]^ Dai et al. developed a phototransistor based on composites of FLG and a‐IGZO that exhibited a giant on/off ratio of 2 × 10^7^ upon UV illumination (350 nm) at 50 mW cm^−2^, which is three orders of magnitude higher than pure a‐IGZO device, and long‐lasting persistent photoconductivity (PPC) that can be retained for years (Figure 13c(i)).^[^ ^137^ ^]^ The extremely long lifetime of photocurrent in FLG/a‐IGZO phototransirtor opens up potential applications by utilizing the precise control over the ON and OFF states, such as optical memory device. It was thought that the long PPC was caused by the greatly reduced recombination probability of the photogenerated electrons and holes, because holes have greater probability of being trapped by large number of defects in the amorphous IGZO, and at a‐IGZO/SiO_2_ interface. These highly trapped holes are highly localized and cannot easily escape from the trapping centers and recombine with the free electrons. For faster photodetecting applications, a positive gate bias must be applied to release the trapped carriers and accelerate the recombination with the free electrons (Figure 13c(ii)).

**Figure 14:**
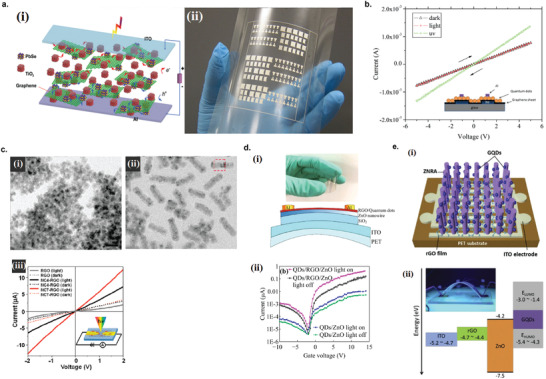
Graphene/multiple semiconductor nanoparticles hybrid photodetectors. a) FLG/PbSe/TiO_2_ hybrid photodetector. i) Schematic representation of multiphase assembly of the FLG/PbSe/TiO_2_ photodetector. ii) Large‐area printed FLG/PbSe/TiO_2_ photodetector patterns on flexible substrate. Reproduced with permission.^[^ ^138^ ^]^ Copyright 2012, WILEY‐VCH. b) Current–voltage characteristic of FLG/CdSe/CdS/ZnS QD hybrid photodetector in dark, under visible illumination, and under UV (365 nm) illumination. Reproduced with permission.^[^ ^139^ ^]^ Copyright 2017, Elsevier B.V. c) rGO/CdSe/ZnS core–shell nanoparticle hybrid photodetector. TEM images of i) [EMIM] (NC4) and ii) [OMIM] (NC7). iii) Current–voltage characteristics of rGO only, rGO/NC4, and rGO/NC7 hybrid photodetectors under AM 1.5 G irradiation (solid lines) and in the dark (dotted lines). Reproduced with permission.^[^ ^109^ ^]^ Copyright 2011, American Chemical Society. d) rGO/CdSe QD/ZnO NW hybrid photodetector. i) Structure of the rGO/CdSe QDs decorated on the surface of ZnO nanowires. ii) Photocurrent variation of two devices with and without rGO under dark and incident light (*V*_ds_ = 5 V, *λ* = 580 nm). Reproduced with permission.^[^ ^110^ ^]^ Copyright 2016, Springer Nature. e) rGO/ZnO NW/GQD hybrid photodetector. i) Schematic diagram of the hybrid photodetector on ITO‐coated PET. ii) Energy band diagram of the hybrid device and the photograph of the fabricated device under test. Reproduced with permission.^[^ ^135^ ^]^ Copyright 2019, Elsevier B.V.

*Graphene/Multiple Semiconductor*: Combining multiphase QDs onto electrochemically‐exfoliated few‐layer graphene (FLG) layer for more broadband photocurrent generation was demonstrated by Manga et al. who fabricated three‐body PbSe/TiO_2_/FLG multiphase hybrid systems that could harvest light from UV to IR regions of the electromagnetic spectrum (**Figure** **14**a).^[^ ^138^ ^]^ Compared to pure PbSe, FLG/PbSe, and FLG/TiO_2_ photodetectors, PbSe/TiO_2_/FLG photodetector showed photoresponse enhancement in both UV and IR regions, which resulted in responsivities of 0.506 A W^−1^ at 350 nm and 0.13 A W^−1^ at 1000 nm. In this architecture, the photocurrent arose from the efficient electron transfer from PbSe QDs to TiO_2_ QDs or FLG. The TiO_2_ QDs serves multiple roles in the device: as electron acceptors, as dielectric filler to limit the dark current in the hybrid device, and also could act as electron donors under UV illumination.^[^ ^138^ ^]^ Al‐Alwani et al. demonstrated a hybrid photodetector composed of monolayer of multiphase CdSe/CdS/ZnS QDs and monolayer of liquid exfoliated few‐layer graphene (FLG) fabricated via Langmuir–Blodgett (LB) technique.^[^ ^139^ ^]^ The LS method has the advantage compared to other liquid deposition methods for more accurate monolayer formation and control of their thickness and structure. With this method, monolayer FLG nanosheets were successfully deposited with 5–8 nm and were covered by ≈10 nm of monolayer CdSe/CdS/ZnS QDs (Figure 14b). The photoresponsivity of the hybrid photodetector with ITO electrodes was 45.77 A W^−1^ under UV illumination at 365 nm.

Song et al. synthesized CdSe/ZnS core/shell nanocrystals (NC) using a series of ionic liquids (IL); phosphonium and imidazolium bis(trifluoromethylsulfonyl)imide ((CF_3_SO_2_)_2_N^−^, [TFSI]) ILs as both a solvent and capping ligand, which enables the control of size, shape and phase (zinc blende (ZB) versus wurtzite (WZ)) of the NCs.^[^ ^109^ ^]^ Specifically, the longer alkyl side chains of the imidazolium cation induced the evolution of the NCs from zinc blende nanodots to wurtzite nanorods. Figure 14c(i) shows the CdSe/ZnS QDs synthesized using 1‐ethyl‐3‐methylimidazolium bis‐(trifluoromethylsulfonyl)imide ([EMIM][TFSI]) average diameters of 7.5 ± 0.5 nm and Figure 14c(ii) shows the CdSe/ZnS nanorods (NR) synthesized using 1‐octyl‐3‐methylimidazolium bis‐(trifluoromethylsulfonyl)imide ([RMIM][TFSI]) with an average diameter of 10 ± 0.5 nm and length of 35 ± 3nm. The average compositions of the CdSe‐rich core and ZnS‐rich shell phases were estimated to be [CdSe]:[ZnS] = 0.75:0.25 and 0.2:0.8. Furthermore, the CdSe/ZnS NCs were linked to the rGO via the noncovalent ionic liquid linkage to fabricate rGO/CdSe/ZnS NCs hybrid photodetector. Figure 14c(iii) shows the *I*–*V* curves of the photodetectors in dark and under AM 1.5G (Xe lamp, 100 mW cm^−2^) irradiation for bare rGO, rGO/CdSe/ZnS QD (NC4‐rGO) and rGO/CdSe/ZnS NR (NC7‐rGO) photodetectors. The generation of photocurrent is the result of efficient IL linkage producing a strong binding interaction between the NCs and RGO, effectively separating the photogenerated electron–hole pairs across the interface. The higher photocurrent of the NR hybrid compared to the QD hybrid might be due to better charge transport efficiency of the rod shape compared to dots.

A novel hybrid phototransistor composed of CdSe QDs and rGO composites decorated on ZnO nanowire (NW) was reported by Tao et al. (Figure 14d(i)).^[^ ^110^ ^]^ The addition of rGO to hybrid structure greatly improved the photoresponsivity of the device compared to that of CdSe QD/ZnO NW structure, increasing the photoresponsivity at 580 nm by approximately two orders of magnitude to 2000 A W^−1^ (Figure 14d(ii)). With incorporation of rGO, the photoexcited electrons can be more efficiently transferred from the conduction band of CdSe QD to rGO due to favorable energy barrier between CdSe interface and rGO. Furthermore, by applying gate voltage, electrons can jump more efficiently from rGO fermi level to defect level, and transfer to ZnO NW. Overall, the more favorable energy configuration of rGO/CdSE QD/ZnO NW hybrid resulted in higher photoresponse compared to CdSe QD/ZnO NW hybrid.

Ko et al. fabricated rGO/ZnO NW/GQD hybrid photodetector on indium tin oxide (ITO)‐coated PET substrate for flexible transparent UV photodetector (Figure 14e(i)).^[^ ^135^ ^]^ Since ITO‐coated PET has a low processing temperature and high surface roughness, which hinders the growth of high‐quality ZnO NRs, the chemically reduced rGO layer was used as a template for low‐temperature growth of vertically‐aligned ZnO NRs and the conductive charge transporting layer. Further decoration with GQDs on the ZnO NRs significantly enhanced the photoresponsivity of the device. Figure 14e(ii) shows the energy band diagram of the different materials in the device. Under UV illumination, electron−hole pairs are created in both ZnO NRs and GQDs. The photogenerated electrons then transfer from GQDs to ZnO NRs, while holes migrate to the surface of ZnO NR surface due to the band bending and combine with negatively charged oxygen ions an induces oxygen desorption (h^+^ + O_2_^−^(ad) → O_2_(g)). The unpaired electrons in the device results in the increase of the photocurrent.

##### Graphene/Carbon‐Based Nanoparticles

**Figure 15:**
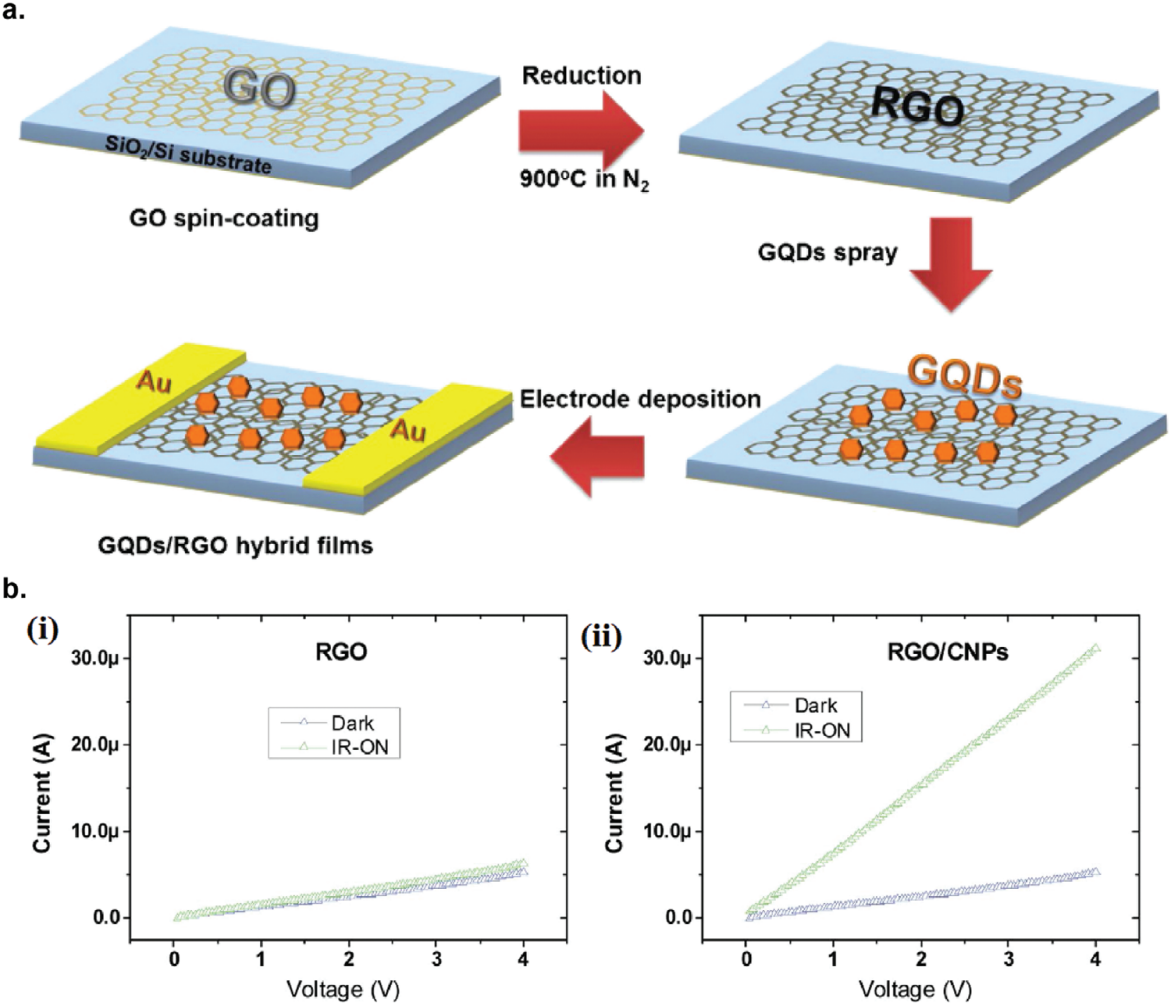
Graphene/carbon‐based nanoparticles hybrid photodetectors. a) Schematic illustration of the fabrication of rGO/GQD hybrid UV photodetector. Reproduced with permission.^[^ ^118^ ^]^ Copyright 2015, Elsevier B.V. b) rGO/carbon nanoparticle (CNP) hybrid photodetector. Current–voltage characteristics of i) pure rGO and ii) rGO/CNP hybrid photodetectors under dark and IR irradiation. Reproduced with permission.^[^ ^120^ ^]^ Copyright 2019, Royal Society of Chemistry.

*Graphene/Graphene Quantum Dot*: Combining GQD with graphene in a hybrid structure opens up a potential pathway for fabricating all graphene‐based material devices with high performance. Tam et al. demonstrated an UV photodetector based on rGO/GQD hybrid film with facile solution process.^[^ ^118^ ^]^ GQDs were synthesized by carbonization of citric acid and were subsequently sprayed onto rGO film spin‐coated on Si/SiO_2_ substrate (**Figure** **15**a). GQDs acted as photoactive material that generated photoexcited carriers by UV light absorption, while rGO film acted as a charge transporting layer due to its superior electrical properties. The efficient charge transfer of photogenerated electrons from GQDs to rGO resulted in high photoresponsivity of 8.7 × 10^2^ A W^−1^ and specific detectivity of 7.7 × 10^13^ Jones at a low operating voltage. Later on, Thanh et al. fabricated a flexible rGO/GQD hybrid photodetector by spray‐coating the hybrid dispersion onto ITO‐coated PET substrate.^[^ ^119^ ^]^ The device showed good transparency and flexibility, and exhibited noticeable ultraviolet photodetection.

*Graphene/Carbon Nanoparticle*: Carbon‐based nanomaterials such as carbon nanoparticles (CNP) are considered as promising green materials and have excellent electrical and optical properties.^[^ ^372^ ^]^ Alam et al. reported the fabrication of rGO/CNP hybrid IR photodetector that could detect human body IR radiation under ambient conditions.^[^ ^120^ ^]^ Figure 15b shows the comparison of *I*–*V* characteristics between bare rGO device and rGO/CNP hybrid device under dark and under IR radiation from a hand kept away at 2 cm from the device at room temperature. The rGO/CNP hybrid photodetector clearly exhibited much higher photoresponse to IR radiation compared to bare rGO device, showing its promising potential as IR sensing applications.

##### Graphene/Perovskite

**Figure 16:**
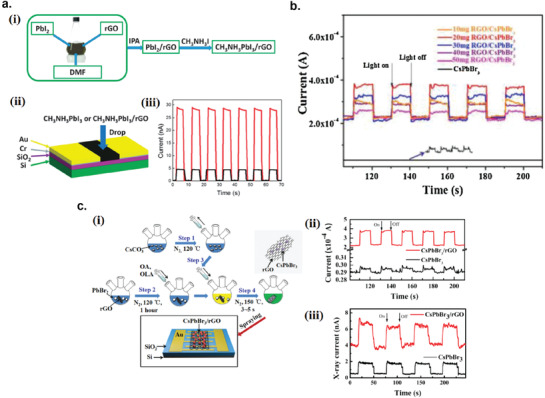
Graphene/perovskite hybrid photodetectors. a) rGO/CH_3_NH_3_PbI_3_ hybrid photodetector. i) Schematic illustration of in situ synthesis of rGO/CH_3_NH_3_PbI_3_ composite solution. ii) Schematic of the fabrication of the hybrid photodetector. iii) Photocurrent transients of the pure CH_3_NH_3_PbI_3_ (black line) and rGO/CH_3_NH_3_PbI_3_ (red line) photodetectors. Reproduced with permission.^[^ ^121^ ^]^ Copyright 2015, Royal Society of Chemistry. b) Photocurrent response of the CsPbBr_3_ nanoparticles and rGO/CsPbBr_3_ hybrid photodetector under different ratio of rGO with and without light irradiation. Reproduced with permission.^[^ ^122^ ^]^ Copyright 2017, Elsevier B.V. c) rGO/CsPbBr_3_ hybrid photodetector for X‐ray photodetection. i) Schematic of fabrication process for rGO/CsPbBr_3_ hybrid photodetector. ii) Photocurrent transients of pure CsPbBr_3_ nanoparticles and the rGO/CsPbBr_3_ hybrid under on/off blue light illumination at 405 nm. iii) Photocurrent transients of pure CsPbBr_3_ nanoparticles and the rGO/CsPbBr_3_ hybrid under on/off X‐ray irradiation. Reproduced with permission.^[^ ^123^ ^]^ Copyright 2018, Elsevier B.V.

Organolead halide perovskites (CH_3_NH_3_PbX_3_, X = Cl, Br, I) has drawn tremendous research interest in recent years due to its interesting optoelectronic properties, such as large light absorption coefficients, wideband absorption, large carrier mobility, and excellent solution processability,^[^ ^373^, ^374^ ^]^ and numerous high‐performing optoelectronical devices such as solar cells and photodetectors have been fabricated based on perovskite thin films.^[^ ^375^, ^376^ ^]^ He et al. fabricated rGO/CH_3_NH_3_PbI_3_ hybrid photodetector via a facile in situ solution method (**Figure** **16**a(i)), which exhibited 6 times higher on/off ratio and faster response speed compared to pure CH_3_NH_3_PbI_3_ photodetector (Figure 16a(ii)).^[^ ^121^ ^]^ The hybrid photodetector exhibited the responsivity of 73.9 mA W^−1^, compared to only 11.1 mA W^−1^ for pure CH_3_NH_3_PbI_3_ photodetector.

CsPbX_3_ (X = Cl, Br, I) perovskite nanoparticles recently has found promising applications in photodetecting devices due to fast separation of photoexcited carriers such as in CsPb(Br/I)_3_ nanorods,^[^ ^377^ ^]^ CsPbBr_3−_ *_x_*I*_x_* nanoparticles^[^ ^122^ ^]^ and CsPbBr_3_ nanosheets.^[^ ^378^ ^]^ Tang et al. fabricated rGO/CsPbBr_3_ hybrid photodetector prepared by a facile hot‐injection method.^[^ ^122^ ^]^ The highest photocurrent was obtained when the amount of rGO mixed with the precursor solution was 20 mg, which leads to most efficient electron–hole separation between CsPbBr_3_ and rGO (Figure 16b).

Detection of X‐ray photons has been indispensable in applications in fields such as security, crystal structure determination, and astronomy.^[^ ^379^, ^380^ ^]^ Recently, it has been demonstrated that soft X‐ray (<10 keV) photons can be absorbed by polycrystalline hybrid organic‐inorganic perovskite CH_3_NH_3_PbX_3_ (X = Cl, Br, I) films.^[^ ^381^ ^]^ However, due to chemical instability of hybrid organic–inorganic perovskite materials, all‐inorganic substitutions based on lead halide perovskites are more desirable. Liu et al. has demonstrated the potential for detecting X‐ray photons using rGO/CsPbBr_3_ hybrid photodetector which was prepared by facile and low‐cost hot‐injection method (Figure 16c(i)).^[^ ^123^ ^]^ In addition to photorespose under blue light at 450 nm (Figure 16c(ii)), the hybrid device also showed a clear photoresponse under exposure to X‐ray source operated with tungsten anode at an acceleration voltage of 140 kV and current of 20 mA (Figure 16c(iii)). As the energy of the X‐ray photon is much higher than the bandgap of CsPbBr_3_, there are differences in photon absorbing, charge separation, and charge collection mechanism compared to the absorption of visible light. Under X‐ray excitation, high‐speed electrons are released through photoelectric ionization process which can induce secondary high‐speed electrons as well as Auger electrons. These electrons can transfer to the rGO layer, leading to electron–hole separation and charge collection. Since the absorption of X‐ray scales as *Z* ^4^/A*E* ^3^, where *Z* is the atomic number and *E* is the X‐ray energy, the high‐Z Pb element in the perovskite plays a vital role in the absorption of X‐ray photons.

##### Graphene/other 2D material

**Figure 17:**
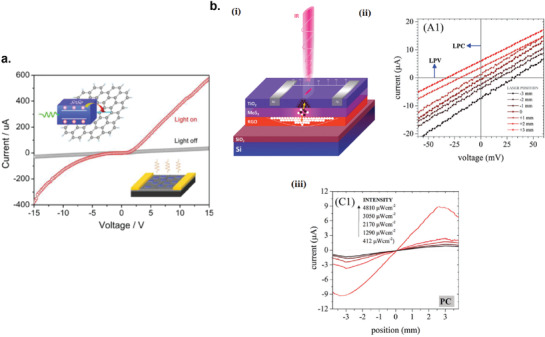
Graphene/other 2D materials hybrid photodetectors. a) Current–voltage characteristic of the rGO/SnSe hybrid photodetector in dark and under white light illumination. Reproduced with permission.^[^ ^124^ ^]^ Copyright 2017, Springer Nature. b) Position‐sensitive detector (PSD) based on an asymmetric TiO_2_/MoS_2_/rGO hybrid photodetector for NIR detection. i) Schematic of the hybrid photodetector. ii) Current–voltage characteristics of the hybrid photodetector under IR illumination at different positions between the two electrodes. iii) The photocurrent of the hybrid photodetector as a function of the position of the IR spot at different IR intensities. Reproduced with permission.^[^ ^125^ ^]^ Copyright 2018, Royal Society of Chemistry.

*Graphene/SnSe*: SnSe is an important p‐type semiconductor that has attracted considerable research interest owing to its narrow bandgap (≈0.90 eV indirect and ≈1.30 eV direct), low toxicity, natural abundance, and chemical stability, and has found numerous applications in solar cells, photodetectors, and near‐infrared optoelectronic devices.^[^ ^382^, ^383^ ^]^ Liu et al. fabricated a hybrid rGO/SnSe photodetector by drop‐casting the rGO/SnSe alcoholic dispersion onto an interdigital gold electrode on SiO_2_ substrate (**Figure** **17**a).^[^ ^124^ ^]^ From the *I*–*V* curve of the device, the on/off ratio of the device reached as large as ≈1110%. As the two components are both 2D materials which have high specific surface area, the interfacial charge transfer in very efficient, improving the overall photosensitivity of the device.

*Graphene/MoS_2_*: Sahatiya et al. demonstrated a large‐area, flexible, paper‐based FLG/MoS_2_ broadband photodetector that could operate in the visible to IR range at room temperature via a low‐cost solution‐processed hydrothermal method.^[^ ^140^ ^]^ The performance of the photodetector under strain was studied after transferring to PDMS substrate. It was discovered that under constant illumination, the photocurrent increased with increasing strain with 79.4% increase in photocurrent enhancement under visible light, and 62.14% increase under IR light, at 2% strain. The mechanism responsible for the enhancement in photocurrent under strain is thought to be the increase in the barrier height for electrons between FLG and MoS_2_, which increases the speed of electron–hole separation due to enhanced interfacial built‐in electric field.

Position‐sensitive detectors (PSD) are optoelectronic devices that are sensitive to the position of the light spot incident on the device surface.^[^ ^384^ ^]^ Thanks to the structural simplicity, high spatial resolution over a large area, and continuous output signal, PSDs are gaining more popularity compared to charge coupled devices. Javadi et al. developed an asymmetric TiO_2_/MoS_2_/rGO sandwich structure for NIR‐sensitive PSD.^[^ ^125^ ^]^ The device configuration is shown in Figure 17b(i). Figure 17b(ii) shows the *I*–*V* curves of the PSD device under localized IR illumination at different positions between the two electrodes, where IR illumination induces a photocurrent at zero bias which changes from ≈−7.1 µA to ≈+6.4 µA by changing the light spot position. The dependence of the photocurrent on the position of the IR illumination spot at zero bias is presented in Figure 17b(iii). Upon illumination, the photogenerated electrons and holes in the MoS_2_ flakes are, respectively, injected to the upper TiO_2_ layer and the bottom rGO layer, effectively separated leading to the long photocurrent lifetime. Since the diffusivity of excess electrons in the TiO_2_ layer is very small, the injected electrons in the TiO_2_ layer leads to a local excess charge in the region of illumination. On the other hand, the injected holes rapidly redistribute themselves uniformly over the rGO layer, due to its high conductivity. This process creates a lateral electric field upon localized illumination, giving rise to photocurrent.

##### Graphene/Organic Material

**Figure 18:**
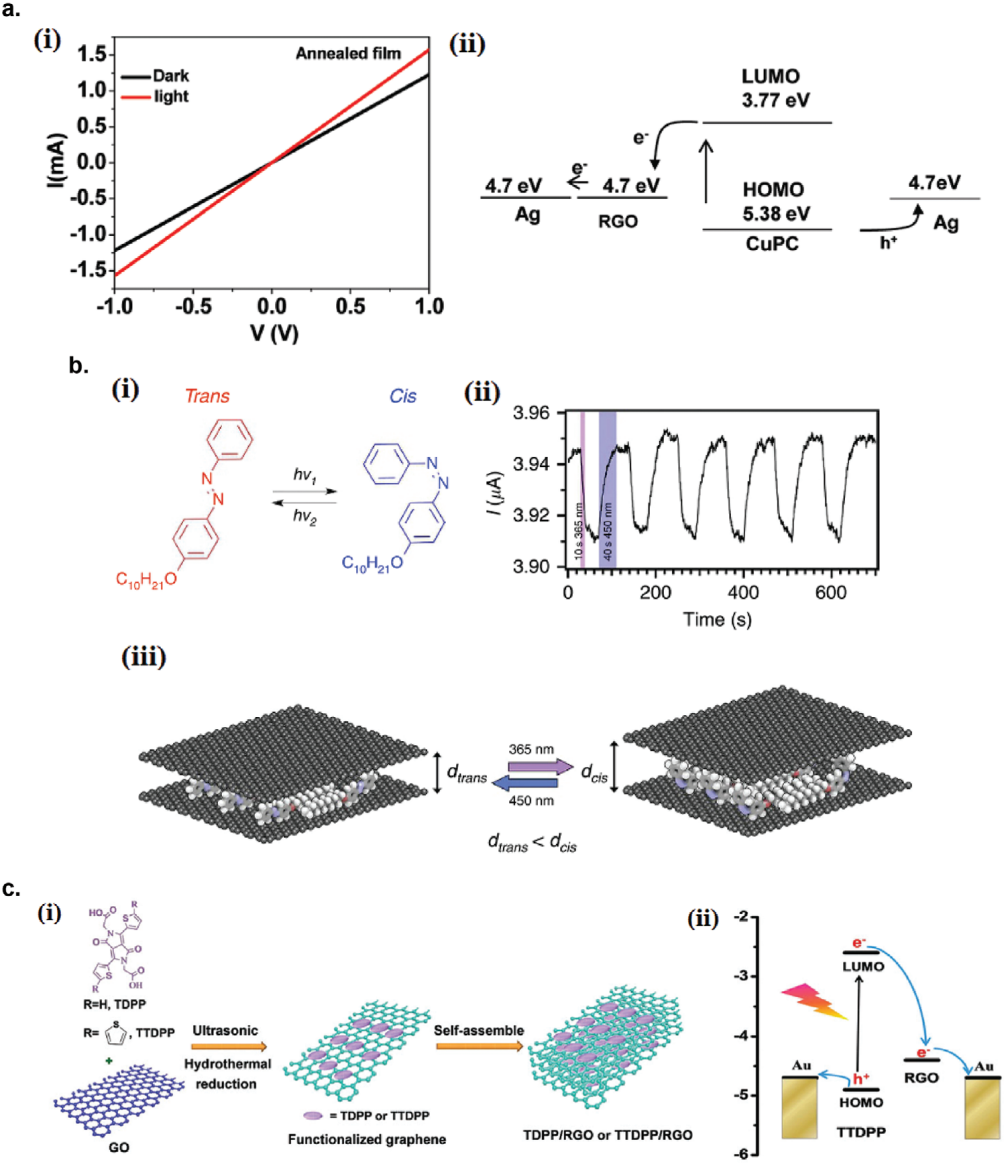
Graphene/molecular organic semiconductor hybrid photodetectors. a) rGO/TSCuPc hybrid photodetector. i) Current–voltage characteristics of the hybrid photodetector in dark and under illumination. ii) Energy level diagram of TSCuPc and rGO. Reproduced with permission.^[^ ^126^ ^]^ Copyright 2010, American Chemical Society. b) FLG/azobenzene hybrid photodetector. i) Chemical structure of 4‐(decyloxy)azobenzene and reversible *trans*–*cis* photoisomerization under UV and visible light. ii) Photocurrent transients of the hybrid device under dynamic alternative UV and visible light irradiation cycles. iii) Schematic of graphene/azobenzene hybrid undergoing UV and visible irradiation cycles. Reproduced with permission.^[^ ^141^ ^]^ Copyright 2016, Springer Nature. c) rGO/diketopyrrolopyrrole derivatives hybrid photodetectors. i) Schematic illustration of preparation of the rGO/TDPP and rGO/TTDPP hybrids. ii) Schematic diagram of the charge‐transfer process in rGO/TTDPP hybrids. Reproduced with permission.^[^ ^127^ ^]^ Copyright 2017, Royal Society of Chemistry.

*Graphene/Molecular Organic Semiconductor*: Molecular semiconductors such as phthalocyanines,^[^ ^385^ ^]^ porphyrines, and perylenes with well‐defined electronic structures^[^ ^386^ ^]^ are known as intrinsic wide bandgap semiconductors (above 1.4 eV) with low intrinsic number of charge carrier in the dark. However, upon doping chemically, electrochemically, or photochemically, extrinsic charge carriers are created. Composites containing rGO and molecular organic semiconductors are expected to have high conductivity and unique optoelectrical properties, making them interesting materials for optoelectronic applications. Chunder et al. fabricated composites of rGO and tetrasulfonate salt of copper phthalocyanine (TSCuPc) via the reduction of GO in the presence of water‐soluble TSCuPc.^[^ ^126^ ^]^ TSCuPc acted as electron donor and rGO acted as electron acceptor. Compared with bare rGO, the rGO/TSCuPc hybrid film has lower conductivity (dark current) but much higher photoresponsivity due to the coexistence of donor/acceptor materials and good interfacial charge transfer of photogenerated carriers. **Figure** **18**a(i) shows the *I*–*V* curve of the hybrid photodetector with rGO:TSCuPc ratio of 1:1.25 under dark and light illumination (xenon lamp, 100 mW cm^−2^). Figure 18a(ii) shows the band diagram of the RGO/TSCuPc device. When photogenerated excitons are dissociated, the holes are collected at the drain electrode from the HOMO level of TSCuPc (5.38 eV) while the electrons are collected from the LUMO level of TSCuPc (3.77 eV) to source electrode through RGO (4.7 eV).

Photochromic molecules like azobenzene‐based molecules, when covalently linked^[^ ^387^ ^]^ or physisorbed to reduced graphene oxide,^[^ ^388^ ^]^ can be used to reversibly modulate the graphene's electronic properties. Furthermore, an alkoxy‐substituted azobenzene, such as 4‐(decyloxy) azobenzene, undergoes a *trans*–*cis* photochemical isomerization which is a large conformational change upon illumination (Figure 18b(i)). When intercalated between graphene layers, this unique property can be exploited to modulate the graphene electronic properties under light illumination due to reversible interlayer distance modulation in the sub‐Å scale caused by photoisomerization. Döbbelin et al. fabricated FLG/azobenzene hybrid photodetector by drop‐casting the suspension of FLG/azobenzene onto pre‐patterned interdigitated gold electrodes.^[^ ^141^ ^]^ The suspension was obtained by ultrasonicating graphene powder directly in the presence of azobenzene in NMP under UV light (365 nm) illumination to induce *trans*–*cis* isomerization of azobenzene molecules, which greatly improved the exfoliation yield due to bulkier nature of the *cis* form compared to the *trans* form. Figure 18b(ii) shows the current modulation of the device under alternate cycles of UV (365 nm) and visible light (450 nm) illumination. Under UV illumination, the azobenzene molecules undergo a conformational change from the less bulky and linear *trans* isomer to the bulkier *cis* isomer. In effect, it increases the interlayer distance of the FLG layers, thereby reducing the charge transport probability via hopping between the layers, resulting in lower conductivity of the hybrid device Figure 18b(iii). Upon illumination with visible light, the *cis*‐to‐*trans* photoisomerization occurs, restoring the conductivity.

1,4‐Diketopyrrolo[3,4‐*c*]pyrrole (DPP) compounds are emerging materials for optoelectronic applications thanks to their large extinction coefficient in the visible‐light region.^[^ ^389^ ^]^ Lin et al. fabricated rGO photodetectors functionalized with diketopyrrolopyrrole derivatives (TDPP or TTDPP) via a solution‐processable method (Figure 18c(i)).^[^ ^127^ ^]^ The hybrid rGO/TDPP and rGO/TTDPP exhibited photoresponsivity of 5.02 and 34.2 A W^−1^, respectively, under white light illumination of 2.34 mW cm^−2^. The excellent performance of the hybrid rGO/TTDPP device is originated from the broad absorption and high extinction spectrum of TTDPP molecules contacting directly with the rGO, efficient charge separation which prevents recombination, and the excellent electronic conductivity of rGO, as schematically shown in energy band diagram in Figure 18c(ii).

**Figure 19:**
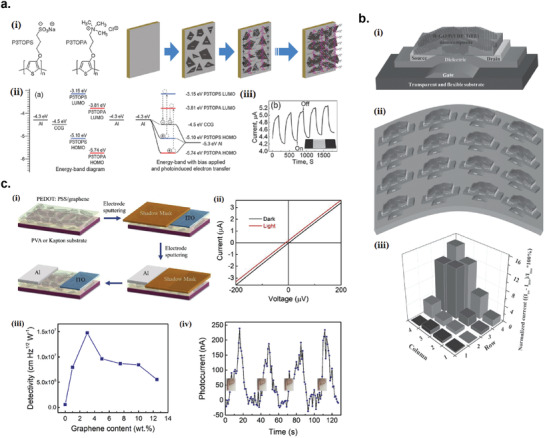
Graphene/polymer hybrid photodetectors. a) rGO/water‐soluble polythiophenes hybrid photodetectors. i) Molecular structures of P3TOPS and P3TOPA and schematic illustration of preparation of rGO–P3TOPS–P3TOPA films by the LBL method. ii) Energy‐band diagram for Al, rGO, P3TOPS, and P3TOPA. iii) Photocurrent transient of the hybrid device under periodical on/off illumination. Reproduced with permission.^[^ ^128^ ^]^ Copyright 2013, Royal Society of Chemistry. b) rGO/ P(VDF‐TrFE) hybrid IR photodetector. i) Structure of the transparent and flexible IR‐responsive rGO/P(VDF‐TrFE) hybrid photodetector. ii) Schematic structure of the device array. iii) The distribution of the normalized current (*I*_DS_ − *I*_DS0_) / *I*_DS0_ × 100% obtained from each device in the array when a finger is moved close to the quadrant of the device array. Reproduced with permission.^[^ ^129^ ^]^ Copyright 2015, WILEY‐VCH. c) FLG/PEDOT:PSS hybrid mid‐IR photodetector. i) Fabrication processes of the rGO/PEDOT:PSS hybrid photodetector. ii) *I*–*V* characteristics of the photodetector measured in the dark and under blackbody illumination. iii) The detectivity as a function of the increasing graphene content within the PEDOT:PSS/graphene composite. iv) Repeated detection of a human fingertip radiation placed above the photodetector surface without contact. Reproduced with permission.^[^ ^142^ ^]^ Copyright 2019, Elsevier Ltd.

*Graphene/Polymer*: Sun et al. synthesized composite films of rGO and water‐soluble polythiophenes (P3TOPS and P3TOPA) by a layer‐by‐layer (LBL) method using the suspension of poly(2‐(3‐thienyloxy)propanesulfonate) (P3TOPS, negatively charged) stabilized rGO sheets and the solution of poly(2‐(3‐thienyloxy)propyl‐ trimethylammonium) (P3TOPA, positively charged) (**Figure** **19**a(i)).^[^ ^128^ ^]^ The polythiophenes and the rGO act as transportation paths for holes and electrons, respectively, forming continuous dual phases. Figure 19a(ii) shows the energy band diagram for the composite device with Al as the source–drain electrodes. The energy offsets between the Fermi level of rGO and the LUMO levels of P3TOPS and P3TOPA leads to the photoinduced electron transfer from the polythiophenes to rGO, leaving holes in the conjugated polymer phase. Figure 19a(iii) shows the current evolution of the device under intermittent on‐and‐off white light illumination.

Trung et al. developed a transparent and flexible IR detector composed of the composites of rGO and poly(vinylidenefl uoride‐*co*‐trifl uoroethylene) (P(VDF‐TrFE)) with a field‐effect transistor (FET) structure (Figure 19b(i)).^[^ ^129^ ^]^ The hydrophobic nature of the copolymer P(VDF‐TrFE) matrix is useful for minimizing the environmental effects caused by polar solvents, moisture, and/ or water vapor on the photodetecting capabilities in ambient conditions and also to enhance the IR sensitivity of the composites. A transparent and flexible array of 16 FETs was fabricated on a flexible and transparent polyethersulfone (PES) substrate by a simple spin‐coating and can respond to IR radiation from a human body (Figure 19b(ii)). Figure 19b(iii) shows the current response of each of the device when a finger was moved near to a quadrant of the device array. In addition to IR absorption by rGO in the composite device, the absorption IR can be enhanced by the polymer matrix, P(VDF‐TrFE)

Despite being an excellent light absorbing material, graphene is not an efficient phothermoelectric material because of the high thermal conductivity. When graphene is mixed with organic polymer, the composite thermal conductivity can be significantly suppressed, thus improving the figure of merit for thermoelectric materials (ZT value). Zhang et al. fabricated a self‐powered, flexible, and semi‐transparent composites of FLG and poly(3,4‐ethylenedioxythiophene): poly(4‐styrenesulfonate) (PEDOT:PSS) mid‐infrared photodetector on polyvinyl alcohol substrate.^[^ ^142^ ^]^ The asymmetric device configuration with Al and ITO as opposite electrode has enabled the detection of broadband mid‐infrared radiation via the mechanism of photothermoelectric (PTE) effect (Figure 19c(i)). Figure 19c(ii) shows the *I*–*V* curves of the device under dark and blackbody irradiation where the zero‐bias photoresponse is due to the asymmetric configuration‐induced photothermoelectric effect. The detectivity peaked at 3 wt% graphene loading at 1.3 × 10^7^ Jones, 22 times larger than pure PEDOT:PSS and 2.5 times larger than pure graphene Figure 19c(iii). The composite device with 3 wt% graphene loading exhibited notable photocurrent when a fingertip was placed ≈2 mm away from the detector (Figure 19c(iv)).

### 6.2 Black Phosphorus Photodetectors

Black phosphorus (BP) is a 2D semiconductor material that has a thickness‐dependent bandgap, transitioning from ≈0.3 eV in bulk to ≈2 eV in monolayer.^[^ ^390^ ^]^ It also has a very high carrier mobility, up to 50 000 cm^2^ V^−1^ s^−1^ in bulk at 30 K.^[^ ^390^ ^]^ These properties suggest that BP has a great potential for optoelectronic applications, such as light emitting diodes, solar cells, and photodetectors.^[^ ^272^, ^391^ ^]^ However, care must be taken for liquid exfoliation of BP due to its instability in the presence of water and oxygen. Therefore, BP exfoliation must be conducted in solvents free of water and oxygen, such as *N*‐methyl‐pyrrolidone (NMP) and *N*‐cyclohexyl‐2‐pyrrolidone (CHP).^[^ ^391^ ^]^

**Figure 20:**
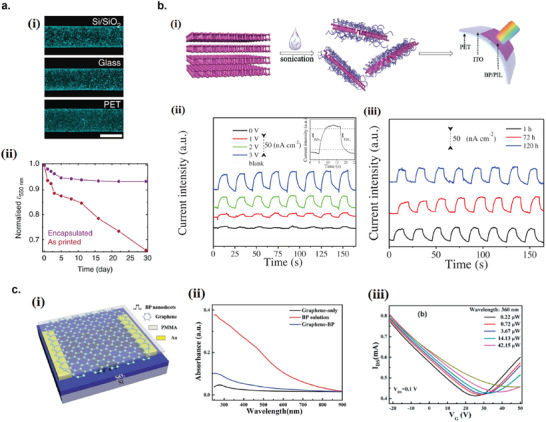
Black phosphorus‐based photodetectors. a) Inkjet printing of liquid‐exfoliated BP. i) Dark field optical micrographs of the optimised printed tracks of BP inks on Si/SiO_2_, glass and PET. Scale bar: 100 µm. ii) Change in optical extinction (at 550 nm) for encapsulated and unencapsulated printed BP across 30 days under ambient conditions. Reproduced with permission.^[^ ^272^ ^]^ Copyright 2017, Springer Nature. b) Conductive polymer ionic liquid (PIL) passivated BP photodetector. i) Schematic illustration of the exfoliation of BP assisted by diluted PIL solution and subsequent photodetector fabrication on a flexible substrate. ii) Photocurrent transients of BP/P([VPIm]TFSI)‐based photodetector under different source–drain bias. Inset graph is the enlarged view of the photocurrent response for rise and decay times. iii) Stability test of the photodetector (1, 72, and 120 h). Reproduced with permission.^[^ ^145^ ^]^ Copyright 2018, WILEY‐VCH. c) BP/graphene hybrid photodetector. i) Schematic diagram of the BP/graphene photodetector. ii) UV–vis absorbance spectra of graphene (with PMMA), BP solution, and the BP/graphene hybrid film (with PMMA). iii) Transfer characteristics of the BP/graphene phototransistor under illumination of 360 nm light with different intensities. Reproduced with permission.^[^ ^146^ ^]^ Copyright 2020, Royal Society of Chemistry.

Hu et al. demonstrated first time the inkjet printing of liquid‐exfoliated BP and invented an ink formula that is suitable for high‐quality inkjet printing of BP.^[^ ^272^ ^]^ The ink composed of liquid‐exfoliated BP nanosheets in a binary solvent of isopropanol (IPA) and 2‐butanol, without any binder. This ink formulation allows stable jetting, minimizes coffee ring effect by inducing recirculating Marangoni flow in the printed droplet and facilitates better wetting even on untreated substrates. The low boiling points of the alcohol mixture enables rapid ink drying that greatly reduces the time taken for BP to interact with the ambient environment, thus reduces the time window for oxidation. The combination of high printing consistency (<2% variation) and high spatial uniformity (<3.4% variation) of the ink formulation makes it suitable for fabrication of stable BP devices (**Figure** **20**a(i)). Pristine BP nanosheets are extremely sensitive to ambient environment and quickly degrades upon exposure to air within few hours.^[^ ^392^ ^]^ To protect the printed BP from the ambient environment, encapsulation with parylene‐C is required. Figure 20a(ii) demonstrate optical extinction of the printed BP samples at 550 nm over 30 days when exposed to ambient conditions. The extinction of the encapsulated BP shows a small decrease (≈5%), whereas the unencapsulated BP the extinction decreases rapidly.

Although encapsulation of BP by insulating capping layer such as polymer can be successful in blocking air and moisture to prevent BP oxidation, it can form charge transport barriers and cause significant decrease in conductivity.^[^ ^393^ ^]^ An alternative strategy to produce stable and conductive BP is to employ conductive passivation coating such as conductive polymers and ionic liquids (IL). Hu et al. fabricated a BP photodetector on a flexible substrate with surface passivation by conductive polymer ionic liquid (PIL) that resulted in excellent stability of the BP layer under ambient conditions, with negligible deterioration of conductivity up to 100 days.^[^ ^145^ ^]^ The fabrication procedure consists of PIL‐assisted exfoliation of BP, surface modification, and fabrication of BP photodetector on a flexible substrate (Figure 20b(i)). The highly charged molecular chains of PILs spontaneously wrap onto the BP nanosheets, providing a surface coverage to protect them from air/humidity attacks. The flexible photodetector was fabricated from the drop‐cast of BP nanosheets exfoliated and encapsulated with P([VPIm]TFSI) on top of ITO/PET substrate. The photocurrent response of BP/P([VPIm]TFSI)‐based flexible photodetector device under light intensity of 20 mW cm^−2^ is shown in Figure 20b(ii), and its stability of performance up to 120 days is shown if Figure 20b(iii).

#### 6.2.1 Black Phosphorus Hybrid Photodetectors

##### Black Phosphorus/Graphene

Zhou et al. fabricated liquid‐exfoliated BP nanosheet decorated CVD‐grown single‐layer graphene phototransistor (Figure 20c(i)).^[^ ^146^ ^]^ Liquid‐exfoliated BP with diverse nanosheet thicknesses and sizes was integrated with graphene by PMMA‐assisted wet transfer, which demonstrated a broadband photoresponse from 360 to 785 nm, due to BP's significant thickness‐dependent bandgap that varies from 0.3 to 2 eV from bulk to monolayer.^[^ ^394^ ^]^ Hybridization of graphene with BP nanosheets enhances the universal absorbance of the device, compared to graphene alone, due to broadband absorption of BP nanosheets (Figure 20c(ii)). In this configuration, the BP nanosheets are responsible for the light harvesting, while the graphene layer is responsible for the fast transit of photogenerated carriers to the electrodes. The photogating effect is mainly responsible for the enhanced photocurrent, due to hole transfer from BP to graphene at the junction during illumination, as can be seen from the shift of the Dirac point of graphene as a function of light intensity (Figure 20c(iii)).^[^ ^146^ ^]^

##### Black Phosphorus/WS_2_

Jia et al. fabricated liquid‐exfoliated BP nanosheets decorated single‐layer WS_2_ phototransistor by drop casting the BP solution on top of CVD‐grown WS_2_ phototransistor.^[^ ^147^ ^]^ Upon the decoration of BP nanosheets, the responsivity and the spectral range of the BP/WS_2_ phototransistor was enhanced. The drop‐casted BP nanosheets on top of WS_2_ formed a p/n heterojunction where BP is p‐type and WS_2_ is n‐type. Upon illumination, due to the built‐in bias at the heterojunction, holes are transferred to BP, while electrons are transferred to the WS_2_ layer. The positive charge in the BP from accumulated holes exerts positive photogating to the WS_2_ channel, increasing the photoconductivity.^[^ ^147^ ^]^

### 6.3 Antimonene

Antimonene is an emerging semiconducting 2D material that has a similar structure to that of BP which has many attractive properties, but its explorations into optoelectronic applications are still rare. Studies have shown that antimonene possesses many unique properties suitable for optoelectronic applications, such as air stability, high carrier mobility, and widely tunable thickness‐dependent bandgap ranging from 0 to 2.28 eV.^[^ ^395^, ^396^ ^]^ Due to its short layer distance and strong binding energy, large‐scale preparation of high‐quality 2D antimonene has been challenging.

#### 6.3.1 Antimonene/CdS

**Figure 21:**
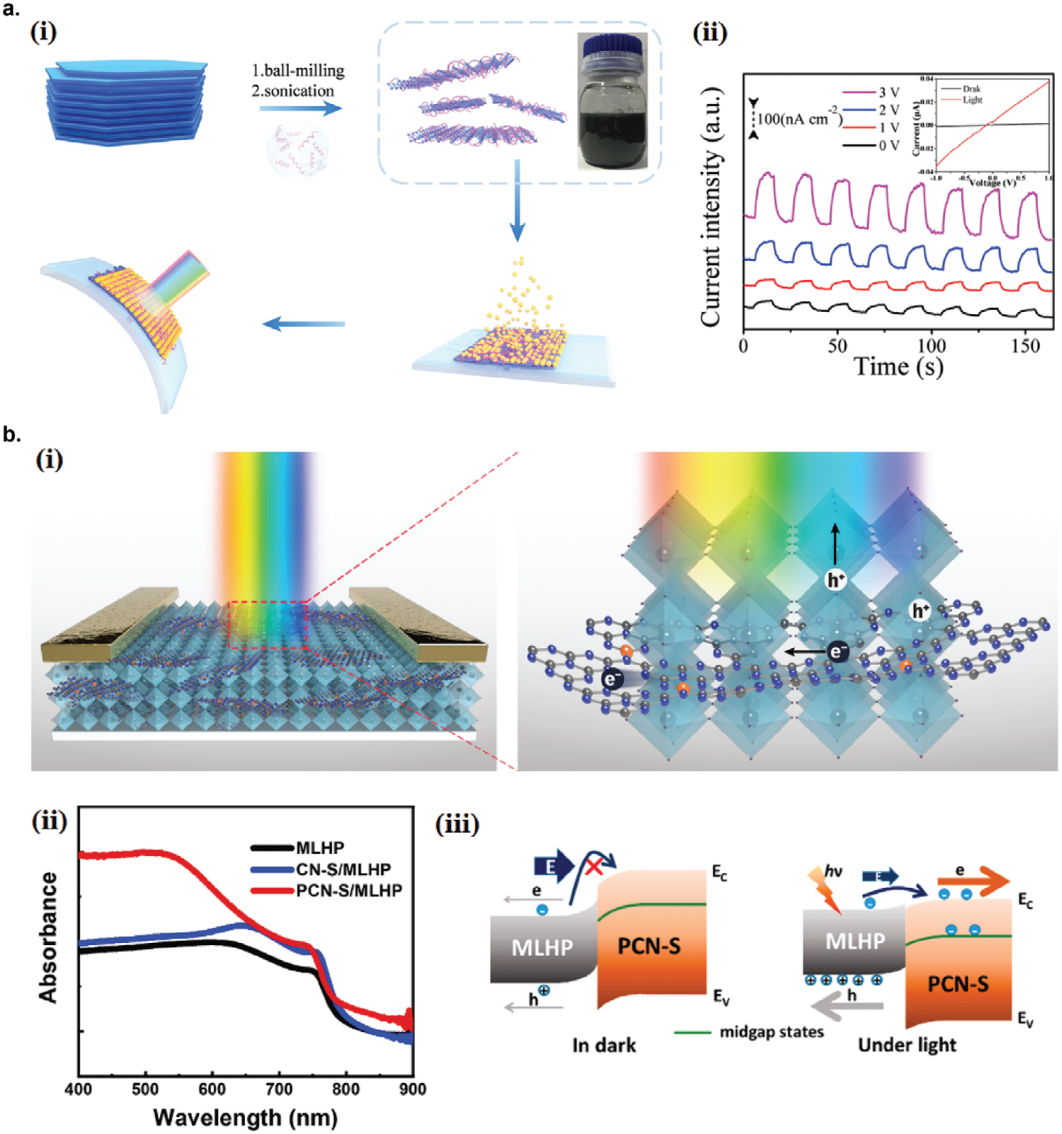
Antimonene and graphitic carbon nitride‐based photodetectors. a) Antimonene/CdS QD hybrid photodetector. i) Schematic illustration of the exfoliation of antimonene assisted by diluted PIL solution. The few‐layer antimonene nanosheets were then used as the active layer by centrifugal cast and hybridized with CdS QDs by a subsequent sequential chemical bath deposition (S‐CBD) method to fabricate the flexible photodetector. ii) Photocurrent transients of Sb/P([VPIm]PF_6_)/CdS QD hybrid photodetector under different source–drain biases. Inset: *I*–*V* characteristics in dark and under illumination. Reproduced with permission.^[^ ^148^ ^]^ Copyright 2020, Royal Society of Chemistry. b) g‐C_3_N_4_/ MLHP hybrid photodetector. i) Schematic representation of the hybrid photodetector. The octahedral stacks represent an MLHP film. ii) UV−vis absorption spectra of MLHP and the hybrids. iii) Band diagram showing the interface between MLHP and PCN‐S in dark and illuminated conditions. Reproduced with permission.^[^ ^149^ ^]^ Copyright 2019, American Chemical Society.

Xiao et al. reported an efficient liquid exfoliation method of antimonene based on diluted polymer ionic liquid (PIL) assisted exfoliation in organic solvent.^[^ ^148^ ^]^ 20% yield of micrometer sized antimonene nanosheets were obtained, which at the time was the highest yield ever reported. **Figure** **21**a(i) shows the preparation of the PIL‐modified antimonene nanosheets, followed by subsequent hybridization with CdS QD to fabricate a flexible photodetector on PET substrate. The exfoliation yield was improved by applying a pregrinding step prior to the liquid‐exfoliation. The concentrated solution of PIL‐modified antimonene nanosheets Sb/P([VPIm]PF6) was centrifugal‐casted on ITO/PET substrate with ITO electrode gap of 100 µm. The CdS QD was hybridized on the top to enhance the response to visible light. The device exhibited the responsivity of 10 µA W^−1^ at 3 V bias, under white light illumination of 20 mW cm^−2^ (Figure 21a(ii)).

### 6.4 Graphitic Carbon Nitride

Graphitic carbon nitride (g‐C_3_N_4_) is a 2D organic semiconductor that has emerged as an attractive optoelectronic material due to its abundance, stability, and nontoxicity.^[^ ^397^ ^]^ Furthermore, g‐C_3_N_4_ has advantages of easy fabrication, metal‐free composition, and low cost compared to other 2D materials like MoS_2_ and WS_2_. Liu et al. fabricated a hybrid photodetector composed of P‐doped g‐C_3_N_4_ nanosheets (PCN‐S) and methylammonium lead trihalide perovskite (MLHP) (Figure 21b(i)).^[^ ^149^ ^]^ Doping g‐C_3_N_4_ with another atom has the advantages of tuning the band gap and tailoring the electronic structure, widening the light absorption range.^[^ ^398^ ^]^ In the experiment, bulk g‐C_3_N_4_ (CN‐B) and PCN‐B were synthesized by a facile thermal polymerization process, using low‐cost precursors (melamine and hydroxyethylidene diphosphonic acid), and subsequently subjected to liquid‐phase exfoliation to nanosheets (PCN‐S) in DMF. Hybridizing PCN‐B and MLHP increases light absorption compared to CN‐B/MLHP and MLHP alone, as shown in Figure 21b(ii). Moreover, the dark current of the PCN‐S/MLHP hybrid photodetector is reduced to 10^−11^ compared to 10^−9^ A for MLHP‐only device, which resulted in higher on/off ratio. A great barrier for electron transport from MLHP to PCN‐S in the dark resulted in low dark current, while under illumination, free electrons and holes generated inside the MLHP reduces the built‐in field between PCN‐S and MLHP, increasing the charge transfer rate at the interface and thus enhancing the photocurrent (Figure 21b(iii)).

### 6.5 Transition Metal Dichalcogenides Photodetectors

Although study on liquid‐exfoliated transition metal dichalcogenides (TMD) for photodetector application is still in its infancy compared to rGO and GQD‐based photodetectors, TMD photodetectors are gaining increased popularity recently as the number of literatures on TMD photodetectors are growing significantly either reported as single materials^[^ ^150^, ^151^, ^152^, ^153^, ^154^, ^155^, ^156^, ^157^, ^158^, ^399^, ^400^, ^401^, ^402^, ^403^, ^404^, ^405^, ^406^, ^407^, ^408^ ^]^ or forming composites with polymers or other nanomaterials.^[^ ^68^, ^161^, ^162^, ^409^, ^410^, ^411^ ^]^ One of the reasons of TMD popularity is that TMDs possess many interesting properties that other 2D materials do not have. These include large variations of electronic properties among different TMDs depending on the combination and structure of metal and chalcogen atoms, which can display either insulating, semiconducting or metallic,^[^ ^412^ ^]^ and layer‐number dependent band structure and bandgap.^[^ ^413^, ^414^, ^415^ ^]^

**Figure 22:**
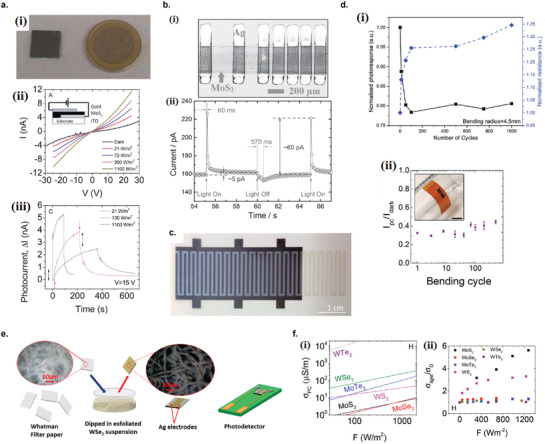
TMD‐based photodetectors. a) MoS_2_ photodetector fabricated by Langmuir–Blodgett method. i) Photograph of the MoS_2_ film prepared after 9 dipping cycles. ii) *I*–*V* curves of the MoS_2_ film in the dark and under various light intensities. iii) Photocurrent transients (after dark current subtraction) under three different light intensities. Reproduced with permission.^[^ ^150^ ^]^ Copyright 2013, Royal Society of Chemistry. b) MoS_2_ photodetector fabricated by inkjet printing. i) Optical micrograph of the inkjet printed MoS_2_ photodetector. The vertical strips are inkjet‐printed silver electrodes, and the horizontal line (≈350 µm wide) is the printed MoS_2_ channel. ii) Photocurrent transient of the device under white light. Reproduced with permission.^[^ ^151^ ^]^ Copyright 2014, WILEY‐VCH. c) Photograph of the inkjet printed MoS_2_ photodetector with inkjet printed interdigitated graphene electrodes. Reproduced with permission.^[^ ^152^ ^]^ Copyright 2014, Royal Society of Chemistry. d) MoS_2_ photodetectors on flexible substrates. i) Changes in the resistance and responsivity of MoS_2_ photodetector on PET substrate upon mechanical flexing. ii) Changes in the photocurrent during bending test over 500 cycles of MoS_2_ photodetector on polyimide substrate. i) Reproduced with permission.^[^ ^154^ ^]^ Copyright 2019, Royal Society of Chemistry. ii) Reproduced with permission.^[^ ^155^ ^]^ Copyright 2019, American Chemical Society. e) Schematic diagram of fabrication of paper‐based photodetector functionalized by WSe_2_ nanosheets. Reproduced with permission.^[^ ^163^ ^]^ Copyright 2019, American Chemical Society. f) Comparative study of a wide range of solution‐processed TMD thin‐film networks. i) Photoconductivity comparisons of a wide range of TMD photodetectors under varying light intensities. ii) Ratio of conductivities under illumination to dark conductivity, plotted versus varying light intensities. Reproduced with permission.^[^ ^156^ ^]^ Copyright 2015, Royal Society of Chemistry.

The first reported liquid‐exfoliated TMD photodetector was an MoS_2_‐based device, reported by Cunningham et al.^[^ ^150^ ^]^ After the MoS_2_ starting powder was exfoliated in isopropanol by ultrasonic tip, the dispersion was transferred to an indium tin oxide (ITO)‐covered glass slide by the Langmuir–Blodgett method (**Figure** **22**a(i)). The final device was completed by evaporating a gold electrode on top of the MoS_2_ layer to give a sandwiched Au/MoS_2_/ITO structure (Figure 22a(ii)). The photoresponse consisted of the fast and slow components (Figure 22a(iii)). The origin of the fast component was not clear, but probably due to bimolecular recombination process.^[^ ^150^ ^]^ On the other hand, the slow component was thought to be originated from the trap states.^[^ ^150^ ^]^ The responsivity was calculated to be 10^−4^ A W^−1^ under the broadband light with low intensity. The photoconductivity *σ*_ph_ scaled roughly with light intensity *F* as *σ*_ph_ ∝ *F^*γ*^* with *γ* = 0.5, is likely associated with the presence of traps. The second reported solution‐processed MoS_2_ photodetector was reported by Li et al.^[^ ^151^ ^]^ MoS_2_ nanosheet ink was synthesized by exfoliation in DMF by bath sonication, then solvent‐exchanged to terpineol through distillation to obtain higher concentration and better printability. The MoS_2_ ink in terpineol were inkjet‐printed onto Si/SiO_2_ substrate with inkjet‐printed silver electrodes, forming a thin film transistor configuration (Figure 22b(i)). The photoresponse of the device showed clear series of peaks and valleys which is due to the presence of traps (Figure 22b(ii)). The photoresponsivity was estimated to be 36 ± 7 µA W^−1^.^[^ ^151^ ^]^ This low responsivity was probably due to the presence of ethyl cellulose residue in the ink formulation, which acted as photocurrent charge carrier traps in the device. Finn et al. demonstrated the inkjet printing of solvent‐exfoliated conductive graphene and MoS_2_ traces with processing temperature no higher than 70 °C to produce all 2D materials printed photodetectors on a semi‐transparent coated PET substrate (Figure 22c).^[^ ^152^ ^]^ This was the first report of the fabrication of printed photodetectors with all components printed from 2D materials ink. The conductance showed tenfold increase compared to the dark conductance during laser illumination (532 nm) with intensity of 640 mW cm^−2^.

McManus et al. first reported the fabrication of all‐printed 2D materials vertical heterostructure photodetector with graphene/WS_2_/graphene vertical structure.^[^ ^153^ ^]^ The redispersion at the interface was prevented by the addition of polysaccharide xanthan gum as binder into the ink formulation that prevents short‐circuit between the graphene electrodes. Another advantage of xanthan gum is that it produces inks with non‐Newtonian viscosity, which helps form uniform printed lines due to its shear‐thinning properties, because the viscosity substantially increases after the droplet is deposited. Xanthan gum is also biocompatible compared to other binders. Park et al. first reported a near‐infrared photodetection to wavelengths up to 1550 nm of chemically exfoliated MoS_2_ thin‐film photodetector.^[^ ^158^ ^]^ The MoS_2_ film was exfoliated using organolithium compounds and contained a mixture of 2H and 1T phases simultaneously. Although the author did not specifically investigate the origin of the extended photoresponse wavelength range to well below the MoS_2_ bandgap, it was thought to be caused by defects formed by Li ion impurities during chemical exfoliation and affected the intrinsic bandgap of MoS_2_, thus extended the spectral range up to 1550 nm.^[^ ^158^ ^]^

The versatility of liquid‐exfoliated TMD enables the high‐concentrated inks to be deposited on variety of flexible and soft substrates, for flexible electronic device applications. Lobo et al.^[^ ^154^ ^]^ and McManus et al.^[^ ^153^ ^]^ fabricated TMD photodetectors on PET substrate, and demonstrated robustness of the device's electrical and photoelectrical performances even after 1000 bending cycles (Figure 22d(i)). Similarly, Seo et al.^[^ ^155^ ^]^ fabricated MoS_2_ photodetectors on polyimide substrate and retained their photosensitivities after 500 bending cycles (Figure 22d(ii)). This proves that the electrical interconnections between nanosheets were not disturbed even after the application of large strains, making them suitable for flexible optoelectronic applications. Besides the flexible polymer substrates, paper‐based optoelectronic devices is also starting to emerge due to its various advantages including low cost, excellent mechanical flexibility, wide availability, recyclability, and biodegradability. Pataniya et al. demonstrated a dip‐coated WSe_2_ photodetector on Whatman filter paper as the substrate (Figure 22e),^[^ ^163^ ^]^ and showed the responsivity of 17.78 mA W^−1^ under 5 V bias, which is comparable to other solution‐processed TMD photodetectors on rigid substrates.^[^ ^150^, ^158^, ^399^, ^408^ ^]^ These demonstrations open up opportunities for large‐scale production of cost‐effective flexible and wearable optoelectronic devices based on nanostructured materials.

The first comparative study of photoconductivity of wide range of solution‐processed TMD thin‐film networks (MoS_2_, MoSe_2_, MoTe2, WS_2_, WSe_2_, and WTe_2_) were first reported by Cunningham et al.^[^ ^156^ ^]^ Significant difference in dark and photoconductivity was observed between materials. The magnitude of photoconductivity scaled as WTe_2_ > WSe_2_ > MoTe_2_ > WS_2_ > MoSe_2_ ≈ MoS_2_ (Figure 22f(i)). The W‐based materials seemed to have higher photoconductivity than Mo‐based materials and bandgap dependence was observed due to different chalcogenide atoms (MTe_2_ > MSe_2_ >MS_2_). However, because the dark conductivity *σ*_D_ also increases as the bandgap decreases according to *σ*_D_ ∝ exp(−*E*_g_ / 1.5*kT*), the ratio of the light conductivity to dark conductivity *σ*_light_/*σ*_D_ are very low for TMDs other than MoS_2_ and WS_2_ (Figure 22f(ii)). Because of this, WSe_2_, WTe_2_ and MoTe_2_ might be more suitable for solar cell material due to lower bandgap and higher conductivity. Similar results were obtained by MacManus et al. for MoS_2_, WS_2_, MoSe_2_, and MoTe_2_ photodetectors printed onto a paper substrate, where MoTe_2_ photodetector showed the highest responsivity, but the lowest photosensitivity due to large dark current.^[^ ^157^ ^]^ Unfortunately, MoTe_2_ photodetector was found to be unstable in air, with responsivity rapidly drops after one day, probably due to oxidation or the formation of conductive octahedra 1T′ phase in addition to the 2H phase.^[^ ^157^ ^]^

#### 6.5.1 TMD Hybrid Photodetectors

##### MoS_2_/PbS

**Figure 23:**
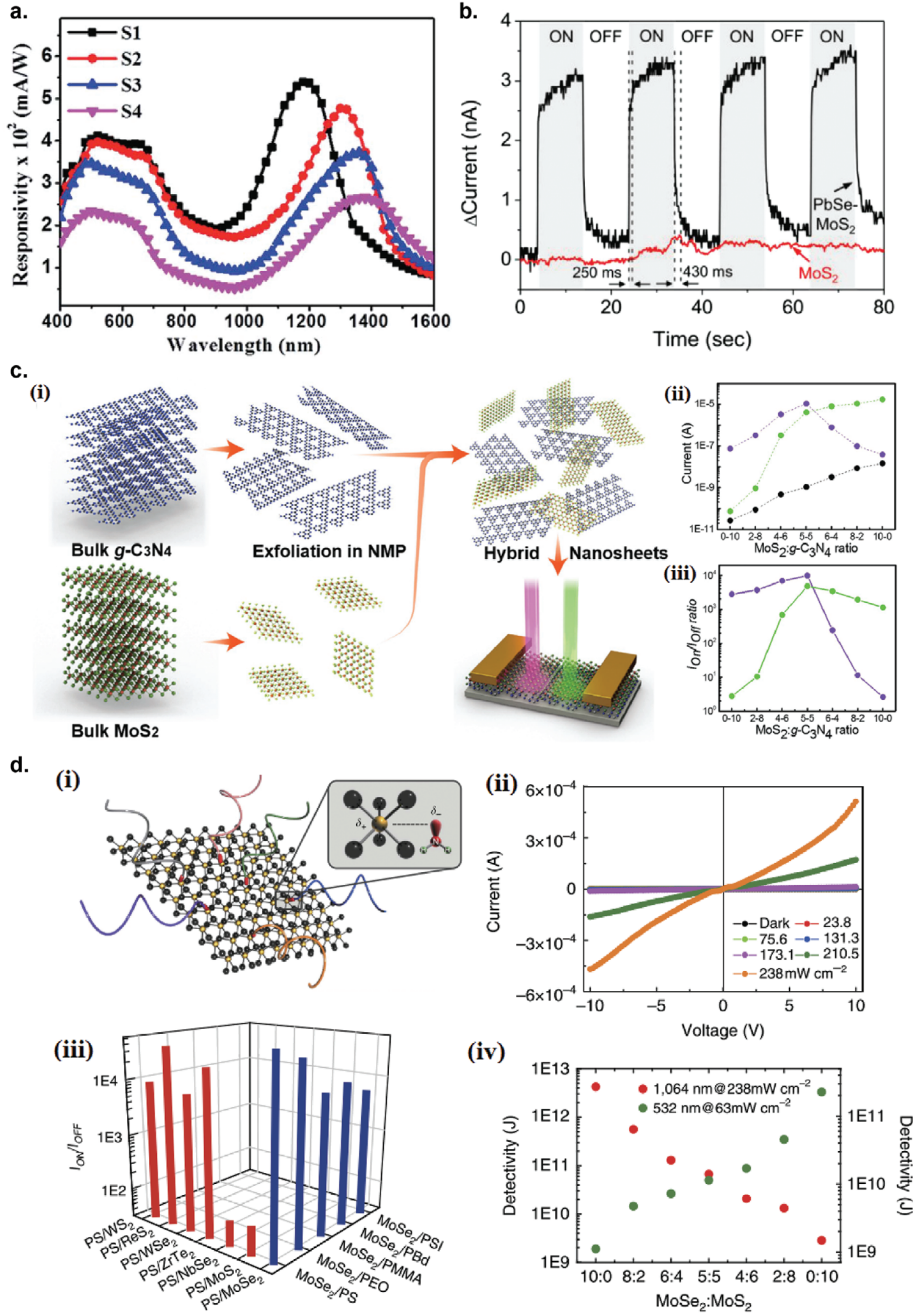
TMD hybrid photodetectors. a) Spectral responsivity of MoS_2_/PbS QD hybrid photodetector under an applied bias of 2 V and 100 mW illumination. S1–S4 refers to the hybrid heterostructure synthesized from the following lead acetate concentration: 1, 5, 10, and 25 mg in 50 mL of aqueous DMF solution. Reproduced with permission.^[^ ^159^ ^]^ Copyright 2019, Royal Society of Chemistry. b) Photocurrent transients of MoS_2_/PbSe QD (black line) and MoS_2_ photodetectors. Reproduced with permission.^[^ ^160^ ^]^ Copyright 2014, WILEY‐VCH. c) MoS_2_/g‐C_3_N_4_ hybrid photodetector on paper substrate. i) Schematic representation of preparation of MoS_2_ and g‐C_3_N_4_ hybrid dispersions and schematic illustration of the hybrid photodetector. ii) Dark (black) and photocurrent (532 nm‐green; 365 nm‐purple), and iii) ratios of the photocurrent to the dark current of the hybrid photodetector as a function of the composition of the MoS_2_ and g‐C_3_N_4_. Reproduced with permission.^[^ ^161^ ^]^ Copyright 2017, WILEY‐VCH. d) TMD/polymer composite photodetectors. i) Schematic representation of hexagonal layers of transition metal atoms (M) sandwiched between two layers of chalcogenides (X) and the interaction between the lone electron pairs of nitrogen atoms of the amine‐terminated polymers and the electron‐accepting metal atoms of the TMD. ii) *I*–*V* characteristics of the composite MoSe_2_ photodetector in the dark and under different light intensities of NIR light at a wavelength of 1064 nm. iii) Ratios of the photocurrent to the dark current of the MoSe_2_ composites with different amine‐terminated polymers and the current ratios of various other TMD composites with PS‐NH_2_. iv) Detectivity as functions of the composition of the MoSe_2_ and MoS_2_ mixtures embedded in PS‐NH_2_ at wavelengths of 532 and 1064 nm. Reproduced with permission.^[^ ^162^ ^]^ Copyright 2015, Springer Nature.

Mukherjee et al. fabricated hybrid MoS_2_/PbS QD photodetector via a one pot, stabilizer‐free solvothermal growth process, and achieved superior optoelectrical response extending to the short‐wavelength infrared region along with a visible response.^[^ ^159^ ^]^ The optical absorption in the NIR region depends on the size of the PbS QDs, controlled by the concentration of lead acetate precursor during the synthesis. The photoresponsivities of the devices with various PbS QD sizes obtained by different lead acetate precursor concentrations (1, 5, 10, and 25 mg in 50 mL aqueous DMF solution) is shown in **Figure** **23**a. The phoresponsivity peak in the NIR regime is ascribed to the optical transitions of the PbS QDs, which exhibits a red shift with increasing PbS QD size due to confinement effect. The hybrid device fabricated with the lowest QDs exhibited the highest responsivity, while the responsivity in both visible and IR regions decreased with increasing the size of PbS QDs. A combined effect of the attenuated illumination, increased surface roughness scattering of carriers and reduced surface area contributes to the reduced responsivity for larger PbS QD size.

##### MoS_2_/PbSe

Schornbaum et al. fabricated hybrid MoS_2_/PbSe QD photodetector via a simple one‐pot synthesis which resulted in a direct epitaxial growth of PbSe QDs on MoS_2_, without the need for ligand exchange steps.^[^ ^160^ ^]^ Epitaxial growth is beneficial as it results in a direct contact interface between PbSe QDs and MoS_2_, without any linker molecule in between, improving charge transfer between the PbSe QDs to the MoS_2_. The hybrid device is solution‐processable at low temperature and stable in air for long time. It exhibited the photosensitivity at near‐infrared region and also shows excellent mechanical stability on a PET substrate upon repeated bending. Figure 23b shows the photoresponse of a pristine MoS_2_ film and a MoS_2_/PbSe QD hybrid film under >1200 nm (1.6 mW) illumination. At wavelengths above 1200 nm, only PbSe QDs are excited, therefore no photoresponse was observed for pure MoS_2_ device.

##### TMD/Other 2D Materials

*MoS_2_/g‐C_3_N_4_*: 2D graphitic carbon nitride (g‐C_3_N_4_) is a promising UV‐ and visible‐light‐active material in the area of solar energy conversion and environmental applications.^[^ ^416^ ^]^ They possess a wide band gap (≈2.7 eV), good stability in ambient conditions, and have low dark current, which make them useful for UV photodetection. On top of that, g‐C_3_N_4_ is also a promising candidate for hybridizing with MoS_2_ because of the crystal lattice matching and the ultrafast charge transfer between MoS_2_ and g‐C_3_N_4_ at the interface.^[^ ^417^ ^]^ Velusamy et al. fabricated mechanically flexible MoS_2_/g‐C_3_N_4_ hybrid photodetector on paper substrate via a simple solution processing, which showed broadband photodetection for both UV and visible spectra (Figure 23c(i)).^[^ ^161^ ^]^ The dark current of MoS_2_/g‐C_3_N_4_ hybrid linearly decreased with increasing in the composition of g‐C_3_N_4_ in the hybrid films due to the resistive nature of g‐C_3_N_4_ (Figure 23c(ii)). Since pure MoS_2_ only has photoresponse to visible region and pure g‐C_3_N_4_ operates only on UV region, hybridizing the two materials not only improves the photodetector performance of the MoS_2_ in the visible region but also extends its photoresponse to the UV region (Figure 23c(iii)).

##### TMD/polymer

Velusamy et al. synthesized various flexible TMD/polymer composite films obtained by liquid exfoliation of numerous TMD nanosheets such as MoS_2_, WS_2_, MoSe_2_, WSe_2_, ReS_2_, ZrTe_2_, and NbSe_2_ in the presence of amine‐terminated polymers.^[^ ^162^ ^]^ The TMDs and polymers are linked via the Lewis‐like acid–base interaction between the transition metal and primary amine (Figure 23d(i)). The TMD/polymer composite photodetectors were fabricated by pouring the mixed suspension onto a filter paper. Under NIR laser illumination with a wavelength of 1064 nm, the composite films generated photocurrent due to photoexcited carriers in the exfoliated TMDs, with maximum on/off ratio of ≈10^5^ was achieved (Figure 23d(ii)). The amine‐terminated polymer matrix may promote photogenerated electron–hole dissociation under illumination by the built up of thermal energy in the insulating polymer around TMDs, and also by enhanced local electric field at the interface of the polymer and TMDs due to the potential barrier at the interface. Various combinations of TMD/polymer composites can be readily developed to obtain NIR‐sensitive photodetectors with various sensitivities (Figure 23d(iii)). Moreover, different absorption peak wavelengths for different TMD materials can be exploited to for band‐selective photodetection by simple mixing of two or more different TMDs. A composite mixture of MoS_2_ and MoSe_2_ functionalized with amine‐terminated polystyrene (PS‐NH_2_) matrix was fabricated which can detect a broad optical spectrum range in both visible and NIR regions. The photosensitivity of the MoS_2_/MoSe_2_/PS‐NH_2_ blended film at NIR and visible regions can be tuned by changing the MoS_2_:MoSe_2_ compositions (Figure 23d(iv)).

### 6.6 Layered III–VI Semiconductors

Layered III−VI semiconductors such as indium(II) selenide (InSe), indium(III) selenide (In_2_Se_2_), and gallium telluride (GaTe) have drawn significant research interest and considered as leading successors to transition metal dichalcogenides (TMDCs) for high‐performance optoelectronics due to their superior electronic and optical properties.^[^ ^418^ ^]^

#### 6.6.1 GaTe

**Figure 24:**
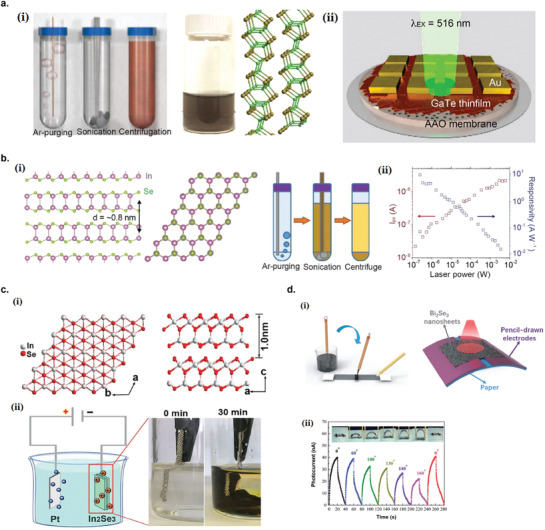
Photodetectors based on layered III–VI semiconductors and topological insulators. a) GaTe photodetector. i) Schematic of the GaTe nanosheet dispersion preparation steps that minimize exposure to ambient air during processing and the atomic structure of layered GaTe (Ga: green, Te: brown). ii) Schematic of an array of percolating GaTe nanosheet thin film photodetectors. Reproduced with permission.^[^ ^164^ ^]^ Copyright 2018, American Chemical Society. b) InSe photodetector. i) Schematic of the layered InSe crystal structure and the schematic of the preparation process for InSe dispersions using deoxygenated ethanol/water mixture. ii) Power dependence of photocurrent *I*_pc_ (red) and responsivity (blue) of InSe photodetector. Reproduced with permission.^[^ ^165^ ^]^ Copyright 2018, WILEY‐VCH. c) In_2_Se_3_ photodetector. i) The atomic structure of layered In_2_Se_3_ crystal. ii) Schematic illustration of the electrochemical exfoliation of bulk In_2_Se_3_. Reproduced with permission.^[^ ^167^ ^]^ Copyright 2020, WILEY‐VCH. d) Bi_2_Se_3_ photodetector fabricated via pencil drawing method. i) Schematic diagram of the device fabrication process using a pencil and Chinese brush on paper. ii) Photocurrent transients of the fexible photodetector under different bending angles. Reproduced with permission.^[^ ^168^ ^]^ Copyright 2020, Royal Society of Chemistry.

Bulk GaTe has a direct bandgap of ≈1.7 eV and shows p‐type semiconducting characteristics, but unlike TMDs like MoS_2_ which shows a transition from indirect bandgap in the bulk to direct bandgap in the monolayer limit, GaTe has a direct bandgap for all layer numbers, providing advantages in optoelectronic applications.^[^ ^419^ ^]^ Kang et al. fabricated GaTe photodetector obtained by liquid‐exfoliation in surfactant‐free, low‐boiling‐point, water‐ethanol cosolvent mixtures (**Figure** **24**a(i,ii)).^[^ ^164^ ^]^ Chemical degradation of GaTe nanosheets were avoided by utilizing a deoxygenated solvents and employing a sealed tip ultrasonication setup. The device exhibited photoresponsivity of 0.2 A W^−1^ under 0.8 W cm^−2^ (516 nm) illumination.

#### 6.6.2 InSe

Layered indium(II) selenide (InSe) has been considered as a leading successor to transition metal dichalcogenides (TMDs) for high‐performance optoelectronic applications owing to its exceptional electronic properties.^[^ ^420^ ^]^ Bulk InSe crystals has a 1.3 eV direct bandgap but when the thickness is decreased to the monolayer limit, the bandgap increases and exhibits a direct‐to‐indirect transition, as opposed to the indirect‐to‐direct transition of TMDs.^[^ ^421^ ^]^ Furthermore, InSe has a higher room‐temperature carrier mobility (exceeding 10^3^ cm^2^ V^−1^ s^−1^) compared to TMDs.^[^ ^420^ ^]^ Kang et al. fabricated liquid‐exfoliated InSe photodetector prepared by liquid exfoliation method in surfactant‐free, deoxygenated cosolvent ethanol‐water cosolvent mixtures without additives to minimize processing residues and performed in a sealed tip ultrasonication setup to avoid chemical degradation (Figure 24b(i)).^[^ ^165^ ^]^ The InSe thin film fabricated via the cosolvents exhibited four orders of magnitude higher conductivity compared to InSe exfoliated in the sodium dodecylsulfate (SDS)–water or NMP, owing to the minimized residual impurities in the film. The illumination power dependence of the photocurrent and the photoresponsivity of the device is shown in Figure 24b(ii). The maximum photoresponsivity reached 10 A W^−1^, confirming the high quality of the film and illustrates that the surfactant‐free, low boiling point, deoxygenated cosolvent approach yields minimal residues that could compromise the conductivity. Recently, Curreli et al. exfoliated bulk InSe in isopropanol and fabricated the InSe‐based percolating photodetector by spray‐coating.^[^ ^166^ ^]^ The device exhibited high photoresponsivity in a broad spectral range (450–900 nm) with maximum achieved at 274 A W^−1^ (at 455 nm), the highest obtained for InSe percolating thin film device.

#### 6.6.3 In_2_Se_3_

In_2_Se_3_ is a layered semiconducting material with thickness‐dependent bandgap (from 1.3 eV in bulk to 2.8 eV for monolayer),^[^ ^422^ ^]^ and displays high absorption coefficients in a broad range of wavelengths (from ultraviolet to short‐wavelength infrared (≈1800 nm)).^[^ ^423^ ^]^ The van der Waals interlayer distance of In_2_Se_3_ is 0.98 nm, much larger than many other layered compounds (Figure 24c(i)), making it amenable to intercalation with guest molecules or ions, especially driven by electric flow in solutions. Shi et al. demonstrated an ultrafast and scalable method to exfoliate In_2_Se_3_ into defect‐free nanosheets via cathodic intercalation in organic electrolytes using tetrahexylammonium (THA^+^) ions as intercalants (Figure 24c(ii)).^[^ ^167^ ^]^ It resulted in a high exfoliation yield of 83%, large sheets with size up to 26 µm and mostly uniform nanosheet thickness of 4 nm, corresponding to three layers. A large‐area photodetector was fabricated based on filtrated In_2_Se_3_ thin films which demonstrated high responsivity (≈1 mA W^−1^) and superfast rise (41 ms) and decay time (39 ms).

### 6.7 Topological Insulators

Topological insulators have been considered recently as a novel class of 2D materials, which exhibit unusual quantum states with conductive states on the surface and insulating states at the interior.^[^ ^424^ ^]^ They have excellent optoelectronic properties with a layer‐dependent band‐gap and polarization‐ sensitive photocurrent, making them promising materials for the new generation of optoelectronic devices.^[^ ^425^ ^]^

#### 6.7.1 Bi_2_Se_3_

Bismuth selenide (Bi_2_Se_3_) has drawn enormous research interest owing to its variety of exciting optoelectronic properties and have a bandgap of 0.3 eV (tunable via layer thickness) as well as high carrier mobility, making it suitable for IR photodetecting applications.^[^ ^426^ ^]^ Liu et al. fabricated a flexible infrared photodetector using liquid‐exfoliated Bi_2_Se_3_ nanosheets via a low‐cost and facile pencil‐drawing method (Figure 24d(i)).^[^ ^168^ ^]^ The device exhibited both significant photocurrent and high responsivity to infrared light (1064 nm) even under bending stress (Figure 24d(ii)).

### 6.8 MXenes

A family of 2D early transition metal carbides/nitrides, MXenes, has drawn tremendous research interest and been extensively studied for a wide range of applications including supercapacitors,^[^ ^427^ ^]^ sensors,^[^ ^428^ ^]^ field‐effect transistors,^[^ ^429^ ^]^ and flexible electrodes.^[^ ^430^, ^431^ ^]^ They are normally produced by a selective etching of an A‐group element (by aqueous hydrofluoric acid (HF),^[^ ^193^ ^]^ fluoride salts with hydrochloric acid,^[^ ^45^ ^]^ or bifluoride‐based etchants, such as NH_4_HF_2_^63^) from layered ternary MAX phases (general formula of M*_n_*_+1_AX*_n_*) where M is an early transition metal, A is an A‐group element (groups 13 and 14), and X is C or N, yielding an aqueous dispersion of individual MXene flakes terminated by fluorine‐ and oxygen‐containing functional groups. These functional groups can modify the work function of MXenes depending on the terminating atoms: OH‐terminated MXenes have ultralow work functions (1.6–2.8 eV), while O‐terminated MXenes have high work functions (5.75–6.25 eV), opening up many interesting applications in electronics.^[^ ^432^ ^]^

#### 6.8.1 Ti_3_C_2_T*_x_*

**Figure 25:**
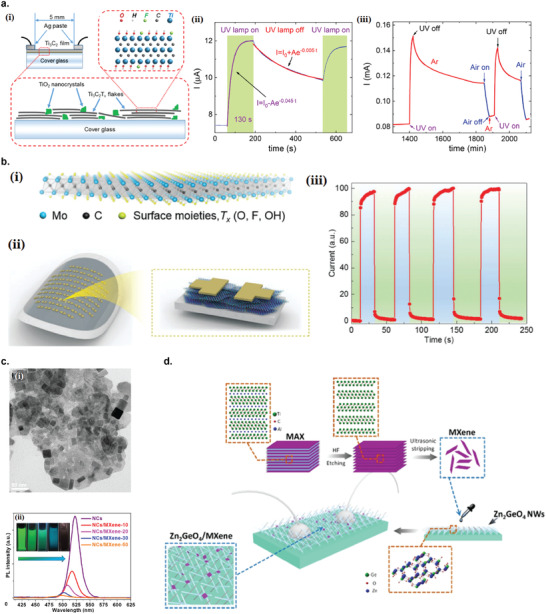
Photodetectors based on MXenes. a) Ti_3_C_2_T*_x_*/TiO_2_ composites photodetector for UV photodetection. i) Schematics of Ti_3_C_2_T*_x_* thin film photodetector where TiO_2_ nanoparticles (green) are present between and on the surface of the MXene flakes. ii) Photocurrent transients of Ti_3_C_2_T*_x_* thin films with and without UV irradiation in Ar gas. iii) Photoresponse of Ti_3_C_2_T*_x_* thin film with and without UV irradiation and exposure to ambient air for 30 min. Reproduced with permission.^[^ ^169^ ^]^ Copyright 2018, American Chemical Society. b) Mo_2_CT*_x_* photodetector. i) Schematic illustration of a single‐layer Mo_2_CT*_x_* nanosheet. ii) Schematic illustration of an array Mo_2_CT*_x_*‐based flexible photodetectors. iii) Photoresponse behavior of Mo_2_CT*_x_* thin film photodetector under alternating on/off cycles at a wavelength of 660 nm with a light intensity of 0.22 W cm^−2^. Reproduced with permission.^[^ ^172^ ^]^ Copyright 2019, WILEY‐VCH. c) Ti_3_C_2_T*_x_*/CsPbBr_3_ hybrid photodetector. i) TEM image of Ti_3_C_2_T*_x_*/CsPbBr_3_ nanocomposite. ii) The photoluminescence spectra of CsPbBr_3_ NCs and CsPbBr_3_/MXene‐*n* nanocomposites. Reproduced with permission.^[^ ^170^ ^]^ Copyright 2019, American Chemical Society. d) Schematic illustration of synthesis of crossed Zn_2_GeO_4_ NWs/ Ti_3_C_2_T*_x_* hybrid structure. Reproduced with permission.^[^ ^171^ ^]^ Copyright 2020, American Chemical Society.

Single layer MXenes in dispersions are not stable toward oxidation in the presence of oxygen.^[^ ^433^ ^]^ For example, oxidation of Ti_3_C_2_ MXene results in the formation and complete conversion into TiO_2_ nanocrystals.^[^ ^434^ ^]^ This oxidation process can be harnessed for spontaneous formation of MXene‐TiO_2_ nanoparticles composites for optoelectronic applications. For that purpose, Chertopalov et al. fabricated Ti_3_C_2_T*_x_*/TiO_2_ composites photodetector that responds to UV irradiation (**Figure** **25**a(i)).^[^ ^169^ ^]^ The titania nanoparticles were mainly located near the edges and on top of MXene flakes. The TiO_2_ nanoparticles act as photosensitive materials that respond to UV irradiation, and the excited photoelectrons transfers to the MXene layers which act as metallic conductors. Photoconductivity measurement in inert atmosphere (argon) exhibited a slow decay of photocurrent (Figure 25a(ii)), which can be used for photosensors with memory effect. However, switching to ambient air during photocurrent relaxation period resulted in a rapid drop of current (Figure 25a(iii)), showing that photoresponse dynamics of Ti_3_C_2_T*_x_*/TiO_2_ composites strongly depends on the environment, which can also be used for gas sensing applications. The origin of reduced current when exposed to ambient air might be the creation of electron traps when molecules such as O_2_ or H_2_O which contain atoms with high electronegativity are adsorbed on the film surface.

#### 6.8.2 Mo_2_CT*_x_*

Velusamy et al. fabricated molybdenum carbide MXene (Mo_2_CT*_x_*) photodetector which exhibited broad photoresponse in the visible range (400–800 nm) with high responsivity (up to 9 A W^−1^) and detectivity (5 × 10^11^ Jones) at 660 nm (Figure 25b (i,ii)).^[^ ^172^ ^]^ The photoresponse is predominantly originated by hot carrier generation assisted by the existence and the distribution of a variety of surface plasmon modes on Mo_2_CT*_x_* nanosheets. This obviates the need for integration with other metallic plasmonic structures, such has been previously demonstrated for several other 2D materials. Figure 25b(iii) shows the photocurrent dynamics of the photodetector under alternating on/off cycles at a wavelength of 660 nm and intensity of 0.22 W cm^−2^, demonstrating good reliability and reproducibility of the photodetector performance. Hong et al. fabricated a photothermoelectric detector based on Mo_2_CT*_x_* ion channel that can convert external light‐induced temperature changes into an open‐circuit voltage due to trans‐nanochannel diffusion potential created by the temperature gradient.^[^ ^435^ ^]^ The photothermoelectric response reached up to 1 mV K^−1^ under one sun illumination.

#### 6.8.3 MXene Hybrid Photodetectors

##### Ti_3_C_2_T*_x_*/CsPbBr_3_ Perovskite

Pan et al. combined Ti_3_C_2_T*_x_* with CsPbBr_3_ perovskite nanocrystals (NCs) forming functional nanocomposites via the in situ growth of the perovskite NCs on 2DTi_3_C_2_T*_x_* nanosheets (Figure 25c(i)).^[^ ^170^ ^]^ It improves the charge separation and transfer following efficient exciton generation in the perovskite NCs, indicated by significant photoluminescence (PL) quenching of the nanocomposites compared to pure CsPbBr_3_ NCs (Figure 25c(ii)), owing to the relative energy level alignment between the CsPbBr_3_ NCs and Ti_3_C_2_T*_x_* nanosheets. The CsPbBr_3_/Ti_3_C_2_T*_x_* nanocomposites demonstrated photocurrent generation in response to visible light and X‐ray illumination.

##### Ti_3_C_2_T*_x_*/ Zn_2_GeO_4_

Zn_2_GeO_4_ is a wide band gap semiconductor (≈4.69 eV) which is useful for deep ultraviolet (DUV) detection.^[^ ^436^ ^]^ Guo et al. combined Ti_3_C_2_T*_x_* MXene with crossed Zn_2_GeO_4_ NWs grown on insulator substrates by a catalyst‐free chemical vapor deposition (CVD) method (Figure 25d).^[^ ^171^ ^]^ The optoelectronic performances of Ti_3_C_2_T*_x_*/Zn_2_GeO_4_ hybrid nanostructures for detecting DUV light was largely improved, exhibiting responsivity of 20.43 mA W^−1^ and external quantum efficiency (EQE) of 9.9% under DUV light illumination at 254 nm. The synergistic effect between DUV detection ability of Zn_2_GeO_4_ and metallic characteristic of Ti_3_C_2_T*_x_* MXene results in a fast electron transport that leads to a larger photocurrent and a fast photoresponse.

## 7 Conclusion and Perspective

In this review, photodetectors based on liquid exfoliation of 2D materials have been given an overview at the latest stage of developments. Various methods of liquid exfoliation of 2D materials have been exploited to obtain dispersions of functional 2D nanosheets that can be directly deposited into devices on any substrates including flexible ones such as plastics and papers, via various techniques such as drop casting, dip coating, inkjet printing, and spray coating. In contrast to traditional fabrication routes such as mechanical exfoliation and vapor‐assisted deposition methods, liquid exfoliation methods are highly scalable and versatile, greatly reducing the cost to produce large quantity of 2D nanosheets in a relatively short time, and are suitable for next generation applications in wearable flexible electronics. Large number of liquid‐exfoliated 2D materials has been successfully applied as photoactive materials in photodetectors, such as graphene, black phosphorus, antimonene, graphitic carbon nitride, TMDs, layered III–VI semiconductors, topological insulators, and MXenes. This list of 2D materials is far from exhaustive, since there are other layered 2D materials that have been successfully exfoliated by liquid exfoliation methods but have yet been reported for applications in photodetectors, such as metal halides,^[^ ^437^, ^438^ ^]^ metal oxides,^[^ ^439^, ^440^ ^]^ and layered double hydroxides.^[^ ^441^, ^442^ ^]^ Therefore, there is still plenty of opportunities for growth and improvements for these diverse groups of materials to be exploited in optoelectronic applications.

**Figure 26:**
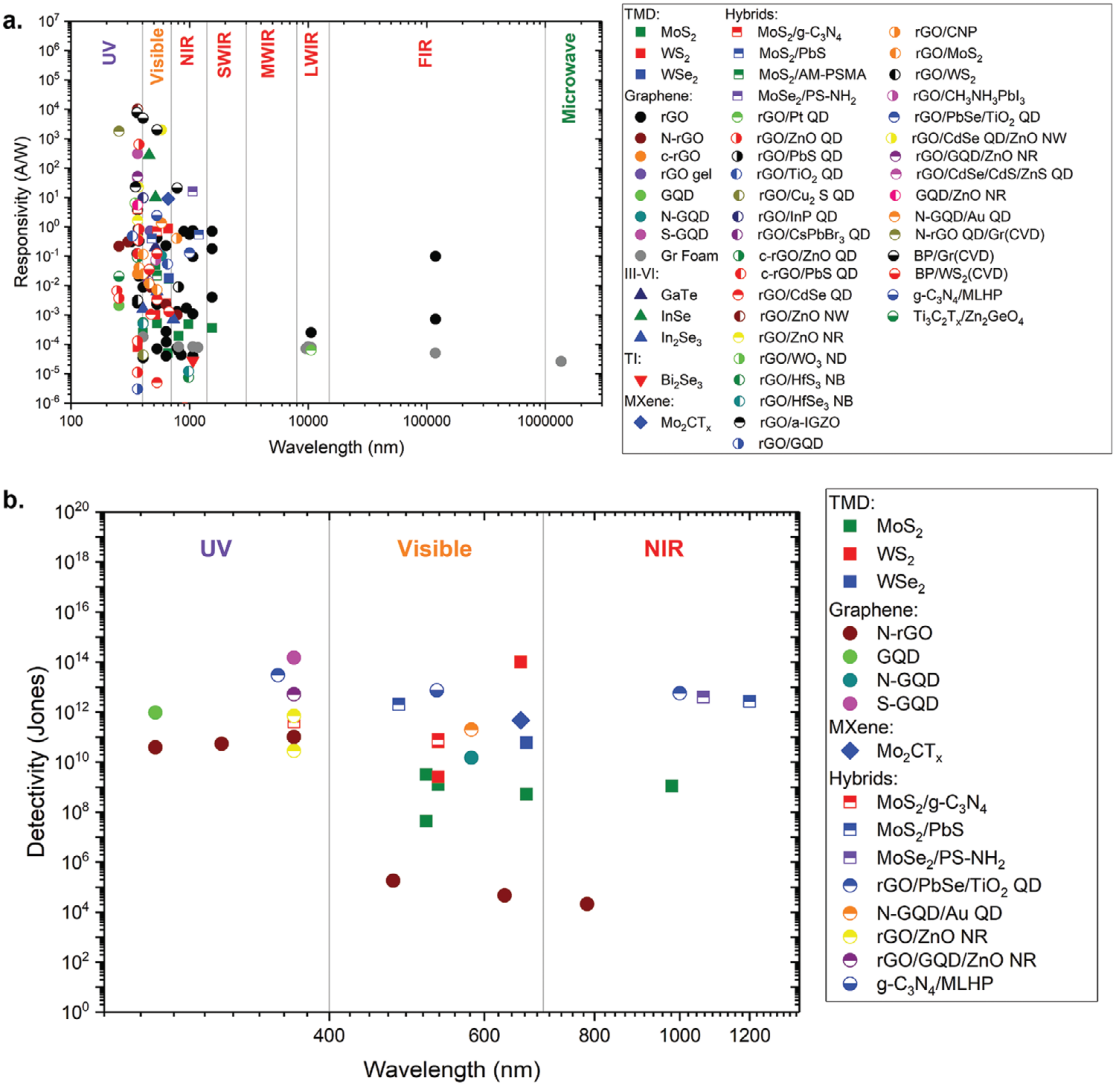
Comparisons of a) photoresponsivities and b) detectivities of the liquid‐exfoliated 2D materials photodetectors summarized in this article. QD, quantum dot; NW, nanowire; NR, nanorod; NB, nanobar; ND, nanodisc.

**Figure** **26** shows the comparison plots of the: a) photorenponsivities; and b) detectivities of the liquid‐exfoliated 2D materials photodetectors summarized in this article as a function of wavelength. The wavelengths have been divided into ultraviolet (UV), visible, near infrared (NIR), short‐wave infrared (SWIR), middle‐wavelength infrared (MWIR), long‐wavelength infrared, far infrared (FIR), and microwave. Note that these plots should not be treated as complete, since only the reported values in the literatures at a specific wavelength are shown. Some of the literatures did not report such values. We did not include the reported photoresponsivities and detectivities under broadband illumination such as white light, since such quantities are defined at a specific wavelength. It can be seen that different range of electromagnetic spectra can be best detected by different 2D materials. For example, in UV range, photodetectors made from rGO/ZnO nanowire (NW) hybrid,^[^ ^443^ ^]^ BP/CVD graphene hybrid,^[^ ^146^ ^]^ and N‐doped rGO QD/CVD graphene hybrid^[^ ^131^ ^]^ resulted in the highest responsivities (>10^3^ A W^−1^), while S‐doped GQD photodetector resulted in the highest detectivity (1.5 × 10^14^ Jones).^[^ ^132^ ^]^ In visible range, BP/CVD graphene hybrid^[^ ^146^ ^]^ and rGO/CdSe QD/ZnO NW hybrid^[^ ^110^ ^]^ resulted in the highest responsivities (>10^3^ A W^−1^), while WS_2_ photodetectors resulted in the highest detectivity (1 × 10^14^ Jones).^[^ ^402^ ^]^ These results seem very promising since these values are already comparable to photodetectors made from high‐quality, single crystalline nanomaterials.^[^ ^299^ ^]^ Therefore, research efforts dedicated to the optoelectronic applications of liquid‐exfoliated 2D materials have begun to show great potentials to compete with the more traditional and more costly single‐crystalline photodetectors.

Despite the promising outlook, there remain some issues that need to be addressed before liquid‐exfoliation of 2D materials can be fully deployed for commercial optoelectronic devices. From the liquid exfoliation point of view, the exfoliated nanosheets tend to have broad range of thickness and lateral size distributions. Even after subjecting to size selection techniques such as band sedimentation and cascade centrifugation, its it still difficult to obtain monodisperse nanosheet distributions with uniform thickness or lateral size with uniformity above 90%.^[^ ^232^, ^236^, ^237^ ^]^ Since many 2D materials exhibit thickness dependent band structure, this will result in nonuniform bandgap distributions across the device volume, which might act as additional scattering centers that lower device performance. Another important issue is the low‐exfoliation yield of liquid exfoliation methods. Liquid exfoliation typically converts typically few percent of the starting bulk 2D powder, thus requires many hours (or days) until the desired amount of exfoliated nanosheets are obtained. There have been a number of pretreatment methods that increase the exfoliation likelihood of the bulk 2D powder such as grinding^[^ ^37^ ^]^ and soaking pretreatment,^[^ ^278^ ^]^ which is a promising step toward reducing the required exfoliation time.

For photodetector applications, one important issue of the liquid‐exfoliated 2D nanosheets is the variability of the response times reported in the literature, and no satisfactory explanations that have been proposed so far.^[^ ^150^, ^155^, ^354^ ^]^ For optical communications applications, photodetectors with very high response speed are required, therefore being able to control the response time is crucial for the next generation of photodetectors to be compatible with the conventional devices. Another important issue which is typical of percolating network of 2D nanosheets is the additional inter‐nanosheet junction resistance that reduces device mobility and can act as carrier trapping centers, thus deteriorating the optoelectronic performances compared to single‐crystalline devices. One way to address this is to introduce conductive filler materials such as conductive polymers between the nanosheets, thus reducing the junction resistance. The amount of the conductive filler should be controlled below the percolating threshold such that shorting does not occur between the opposing electrodes that will increase the dark current. Also, on average, the as deposited percolating network of 2D materials typically have porosity around ≈0.5.^[^ ^250^, ^444^, ^445^ ^]^ This high level of porosity might decrease the efficiency of inter‐nanosheet charge transfer, since only small amount of contact points are available for efficient charge transfer. Efforts to reduce the porosity and increase the compactness of the 2D nanosheet network such as applying pressure will increase the contact area between the nanosheets which reduce the junction resistance and might increase the mobility.

Another issue with most exfoliated 2D materials for applications in image sensors is their broadband nature of the photoresponse. For standard camera sensors, arrays of optical filters are integrated on top of photoactive layer to attain color selectivity. Not only this adds complications into the device architecture and fabrication, they also have other undesirable effects such as reduction in spatial resolution and inconsistency in color detection under different levels of illumination. It is less of an issue for reproducing standard color photography, but for more sophisticated applications such as machine vision and object recognition, using a highly‐color selective materials is especially attractive. A new approach, which is termed charge collection narrowing (CCN), has been shown to deliver color selective spectral response despite using a broadband absorber material. This method is based on the idea that for a thick photoactive material, only photons with lower extinction coefficient are able to generate electron–hole pairs at the bottom part of the material, and by collecting the photogenerated carriers at the bottom, spectrally selective photoresponse can be attained. This approach has been successfully applied for solution‐processable organic, perovskite, and quantum‐dot photodetectors, which resulted in narrow spectral response below 100 nm.^[^ ^446^, ^447^, ^448^, ^449^ ^]^ Therefore, it is expected that the application of this approach will also be useful for liquid‐exfoliated 2D material photodetectors, especially when considering the growing number of available 2D materials, which will potentially open a new era for imaging technology.

Reports of photodetection devices based on liquid‐exfoliated 2D materials has progressed considerably in the past decade, as a result of advances in materials science and device engineering, and also due to growing number of new 2D materials being discovered or synthesized. Combination of low‐cost and facile liquid exfoliation methods and large variation of electronic and optical properties brought about by 2D materials and their high tunability via thickness and size control will certainly bring a promising and exciting future for the next‐generation photodetectors.

## Conflict of Interest

The authors declare no conflict of interest.

## References

[1] R. Hui , Introduction to Fiber‐Optic Communications, Academic Press, Cambridge, MA 2019.

[2] B. Nabet , Photodetectors: Materials, Devices and Applications, Woodhead Publishing, Sawston, CA 2015.

[3] E. SöDerberg , P. Modh , J. S. Gustavsson , A. Larsson , Z. Z. Zhang , J. Berggren , M. Hammar , Electron. Lett. 2006, 42, 978.

[4] Z. Huang , J. E. Carey , M. Liu , X. Guo , E. Mazur , J. C. Campbell , Appl. Phys. Lett. 2006, 89, 033506.

[5] M. Ilegems , B. Schwartz , L. A. Koszi , R. C. Miller , Appl. Phys. Lett. 1978, 33, 629.

[6] C. Li , W. Huang , L. Gao , H. Wang , L. Hu , T. Chen , H. Zhang , Nanoscale 2020, 12, 2201. 31942887 10.1039/c9nr07799e

[7] F. P. García De Arquer , A. Armin , P. Meredith , E. H. Sargent , Nat. Rev. Mater. 2017, 2, 16100.

[8] D. I. Son , H. Y. Yang , T. W. Kim , W. I. Park , Composites, Part B 2014, 69, 154.

[9] H. Tetsuka , A. Nagoya , S.‐I. Tamura , Nanoscale 2016, 8, 19677. 27858051 10.1039/c6nr07707b

[10] B. A. Albiss , M.‐A. Al‐Akhras , I. Obaidat , Int. J. Environ. Anal. Chem. 2015, 95, 339.

[11] M. Kataria , K. Yadav , G. Haider , Y. M. Liao , Y.‐R. Liou , S.‐Y. Cai , H.‐I. Lin , Y. H. Chen , C. R. Paul Inbaraj , K. P. Bera , H. M. Lee , Y.‐T. Chen , W.‐H. Wang , Y. F. Chen , ACS Photonics 2018, 5, 2336.

[12] Z.‐Y. Peng , J.‐L. Xu , J.‐Y. Zhang , X. Gao , S.‐D. Wang , Adv. Mater. Interfaces 2018, 5, 1800505.

[13] M. Wang , F. Cao , L. Meng , W. Tian , L. Li , Adv. Mater. Interfaces 2019, 6, 1801526.

[14] D. Vankhade , T. K. Chaudhuri , AIP Conf. Proc. 2018, 1942, 060001.

[15] R. S. Veerla , P. Sahatiya , S. Badhulika , J. Mater. Chem. C 2017, 5, 10231.

[16] J. Gao , S. C. Nguyen , N. D. Bronstein , A. P. Alivisatos , ACS Photonics 2016, 3, 1217.

[17] W. Zhou , Y. Shang , F. P. García , D. Arquer , K. Xu , R. Wang , S. Luo , X. Xiao , X. Zhou , R. Huang , E. H. Sargent , Z. Ning , Nat. Electron. 2020, 3, 251.

[18] Y. Shen , X. Yan , Z. Bai , X. Zheng , Y. Sun , Y. Liu , P. Lin , X. Chen , Y. Zhang , RSC Adv. 2015, 5, 5976.

[19] D. Liu , H.‐J. Li , J. Gao , S. Zhao , Y. Zhu , P. Wang , D. Wang , A. Chen , X. Wang , J. Yang , Nanoscale Res. Lett. 2018, 13, 261. 30167797 10.1186/s11671-018-2672-5 PMC6117230

[20] L. Yang , D. K. Nandakumar , L. Miao , L. Suresh , D. Zhang , T. Xiong , J. V. Vaghasiya , K. C. Kwon , S. Ching Tan , Joule 2020, 4, 176.

[21] A. Littig , H. Lehmann , C. Klinke , T. Kipp , A. Mews , ACS Appl. Mater. Interfaces 2015, 7, 12184. 25989915 10.1021/acsami.5b02547

[22] A. Afal , S. Coskun , H. Emrah Unalan , Appl. Phys. Lett. 2013, 102, 043503.

[23] S. Zhang , Y. Ma , L. Suresh , A. Hao , M. Bick , S. C. Tan , J. Chen , ACS Nano 2020, 14, 9282. 32790347 10.1021/acsnano.0c03268

[24] E. Lhuillier , J.‐F. Dayen , D. O. Thomas , A. Robin , B. Doudin , B. Dubertret , Nano Lett. 2015, 15, 1736. 25650627 10.1021/nl504414g

[25] A. Robin , E. Lhuillier , B. Dubertret , MRS Adv. 2016, 1, 2187.

[26] Q. Zhang , Q. Liang , D. K. Nandakumar , S. K. Ravi , H. Qu , L. Suresh , X. Zhang , Y. Zhang , L. Yang , A. T. S. Wee , S. C. Tan , Energy Environ. Sci. 2020, 13, 2404.

[27] J. Wang , B. Liu , Sci. Technol. Adv. Mater. 2019, 20, 992. 31692852 10.1080/14686996.2019.1669220 PMC6818124

[28] F. I. Alzakia , B. Tang , S. J. Pennycook , S. C. Tan , Mater. Horiz. 2020, 7, 3325.

[29] L. Yang , L. Loh , D. K. Nandakumar , W. Lu , M. Gao , X. L. C. Wee , K. Zeng , M. Bosman , S. C. Tan , Adv. Mater. 2020, 32, 2000971. 10.1002/adma.202000971 32363694

[30] D. Yang , D. Ma , Adv. Opt. Mater. 2019, 7, 1800522.

[31] J. Huang , J. Lee , J. Vollbrecht , V. V. Brus , A. L. Dixon , D. X. Cao , Z. Zhu , Z. Du , H. Wang , K. Cho , G. C. Bazan , T.‐Q. Nguyen , Adv. Mater. 2020, 32, 1906027. 10.1002/adma.201906027 31714629

[32] J. Yang , X. Zhang , H. Qu , Z. G. Yu , Y. Zhang , T. J. Eey , Y.‐W. Zhang , S. C. Tan , Adv. Mater. 2020, 32, 2002936. 10.1002/adma.202002936 32743963

[33] L. Zheng , T. Zhu , W. Xu , L. Liu , J. Zheng , X. Gong , F. Wudl , J. Mater. Chem. C 2018, 6, 3634.

[34] X. Liu , H. Wang , T. Yang , W. Zhang , X. Gong , ACS Appl. Mater. Interfaces 2012, 4, 3701. 22698063 10.1021/am300787m

[35] N. Paul , L. Suresh , J. V. Vaghasiya , L. Yang , Y. Zhang , D. K. Nandakumar , M. R. Jones , S. C. Tan , Biosens. Bioelectron. 2020, 165, 112423. 32729541 10.1016/j.bios.2020.112423

[36] A. C. Ferrari , F. Bonaccorso , V. Fal'ko , K. S. Novoselov , S. Roche , P. Bøggild , S. Borini , F. H. L. Koppens , V. Palermo , N. Pugno , J. A. Garrido , R. Sordan , A. Bianco , L. Ballerini , M. Prato , E. Lidorikis , J. Kivioja , C. Marinelli , T. Ryhänen , A. Morpurgo , J. N. Coleman , V. Nicolosi , L. Colombo , A. Fert , M. Garcia‐Hernandez , A. Bachtold , G. F. Schneider , F. Guinea , C. Dekker , M. Barbone , et al., Nanoscale 2015, 7, 4598. 25707682 10.1039/c4nr01600a

[37] Y. Yao , L. Tolentino , Z. Yang , X. Song , W. Zhang , Y. Chen , C.‐P. Wong , Adv. Funct. Mater. 2013, 23, 3577.

[38] G. Cunningham , M. Lotya , C. S. Cucinotta , S. Sanvito , S. D. Bergin , R. Menzel , M. S. P. Shaffer , J. N. Coleman , ACS Nano 2012, 6, 3468. 22394330 10.1021/nn300503e

[39] S. C. Dhanabalan , J. S. Ponraj , Z. Guo , S. Li , Q. Bao , H. Zhang , Adv. Sci. 2017, 4, 1600305. 10.1002/advs.201600305 PMC5473329 28638779

[40] Z. Guo , H. Zhang , S. Lu , Z. Wang , S. Tang , J. Shao , Z. Sun , H. Xie , H. Wang , X.‐F. Yu , P. K. Chu , Adv. Funct. Mater. 2015, 25, 6996.

[41] C. Gibaja , D. Rodriguez‐San‐Miguel , P. Ares , J. Gómez‐Herrero , M. Varela , R. Gillen , J. Maultzsch , F. Hauke , A. Hirsch , G. Abellán , F. Zamora , Angew. Chem., Int. Ed. 2016, 55, 14345. 10.1002/anie.201605298 PMC5113666 27529687

[42] J. Ji , X. Song , J. Liu , Z. Yan , C. Huo , S. Zhang , M. Su , L. Liao , W. Wang , Z. Ni , Y. Hao , H. Zeng , Nat. Commun. 2016, 7, 13352. 27845327 10.1038/ncomms13352 PMC5116078

[43] L. Ren , X. Qi , Y. Liu , G. Hao , Z. Huang , X. Zou , L. Yang , J. Li , J. Zhong , J. Mater. Chem. 2012, 22, 4921.

[44] B. Se , B. Te , A. Ambrosi , J. Luxa , M. Pumera , ACS Nano 2016, 10, 11442. 27936571 10.1021/acsnano.6b07096

[45] A. Feng , Y. Yu , Y. Wang , F. Jiang , Y. Yu , L. e Mi , L. Song , Mater. Des. 2017, 114, 161.

[46] J.‐C. Lei , X. Zhang , Z. Zhou , Front. Phys. 2015, 10, 276.

[47] Z. H. Ni , T. Yu , Y. H. Lu , Y. Y. Wang , Y. P. Feng , Z. X. Shen , ACS Nano 2008, 2, 2301. 19206396 10.1021/nn800459e

[48] Q. Yue , S. Chang , J. Kang , X. Zhang , Z. Shao , S. Qin , J. Li , J. Phys.: Condens. Matter 2012, 24, 335501. 22813480 10.1088/0953-8984/24/33/335501

[49] K. I. Bolotin , K. J. Sikes , Z. Jiang , M. Klima , G. Fudenberg , J. Hone , P. Kim , H. L. Stormer , Solid State Commun. 2008, 146, 351.

[50] W. Bao , X. Cai , D. Kim , K. Sridhara , M. S. Fuhrer , Appl. Phys. Lett. 2013, 102, 042104.

[51] H. S. Lee , S.‐W. Min , Y.‐G. Chang , M. K. Park , T. Nam , H. Kim , J. H. Kim , S. Ryu , S. Im , Nano Lett. 2012, 12, 3695. 22681413 10.1021/nl301485q

[52] X. Han , H. M. Stewart , S. A. Shevlin , C. R. A. Catlow , Z. X. Guo , Nano Lett. 2014, 14, 4607. 24992160 10.1021/nl501658d

[53] J. Lu , J. Yang , A. Carvalho , H. Liu , Y. Lu , C. H. Sow , Acc. Chem. Res. 2016, 49, 1806. 27589013 10.1021/acs.accounts.6b00266

[54] X. Cao , Z. Yin , H. Zhang , Energy Environ. Sci. 2014, 7, 1850.

[55] X. Huang , Z. Zeng , H. Zhang , Chem. Soc. Rev. 2013, 42, 1934. 23344899 10.1039/c2cs35387c

[56] R. C. Sinclair , J. L. Suter , P. V. Coveney , Phys. Chem. Chem. Phys. 2019, 21, 5716. 30801077 10.1039/c8cp07796g

[57] H. Li , J. Wu , Z. Yin , H. Zhang , Acc. Chem. Res. 2014, 47, 1067. 24697842 10.1021/ar4002312

[58] V. Nicolosi , M. Chhowalla , M. G. Kanatzidis , M. S. Strano , J. N. Coleman , Science 2013, 340, 1226419.

[59] L. Niu , J. N. Coleman , H. Zhang , H. Shin , M. Chhowalla , Z. Zheng , Small 2016, 12, 272. 26663877 10.1002/smll.201502207

[60] J. Chen , B. Yao , C. Li , G. Shi , Carbon 2013, 64, 225.

[61] H. Yu , B. Zhang , C. Bulin , R. Li , R. Xing , Sci. Rep. 2016, 6, 36143. 27808164 10.1038/srep36143 PMC5093679

[62] X. Fan , P. Xu , D. Zhou , Y. Sun , Y. C. Li , M. A. T. Nguyen , M. Terrones , T. E. Mallouk , Nano Lett. 2015, 15, 5956. 26288218 10.1021/acs.nanolett.5b02091

[63] M. Alhabeb , K. Maleski , B. Anasori , P. Lelyukh , L. Clark , S. Sin , Y. Gogotsi , Chem. Mater. 2017, 29, 7633.

[64] J. N. Coleman , M. Lotya , A. O'neill , S. D. Bergin , P. J. King , U. Khan , K. Young , A. Gaucher , S. De , R. J. Smith , I. V. Shvets , S. K. Arora , G. Stanton , H. ‐ Y. Kim , K. Lee , G. T. Kim , G. S. Duesberg , T. Hallam , J. J. Boland , J. J. Wang , J. F. Donegan , J. C. Grunlan , G. Moriarty , A. Shmeliov , R. J. Nicholls , J. M. Perkins , E. M. Grieveson , K. Theuwissen , D. W. Mccomb , P. D. Nellist , V. Nicolosi , Science 2011, 331, 568. 21292974 10.1126/science.1194975

[65] D. Jariwala , T. J. Marks , M. C. Hersam , Nat. Mater. 2017, 16, 170. 27479211 10.1038/nmat4703

[66] X.‐X. Yu , H. Yin , H.‐X. Li , H. Zhao , C. Li , M.‐Q. Zhu , J. Mater. Chem. C 2018, 6, 630.

[67] X. Geng , Y. Yu , X. Zhou , C. Wang , K. Xu , Y. Zhang , C. Wu , L. Wang , Y. Jiang , Q. Yang , Nano Res. 2016, 9, 2641.

[68] I. Hwang , J. S. Kim , S. H. Cho , B. Jeong , C. Park , ACS Appl. Mater. Interfaces 2018, 10, 34543. 30205685 10.1021/acsami.8b07279

[69] Y. Zhang , F. Zhang , Y. Xu , W. Huang , L. Wu , Y. Zhang , X. Zhang , H. Zhang , Adv. Funct. Mater. 2019, 29, 1906610.

[70] J. Yang , X. Hu , X. Kong , P. Jia , D. Ji , D. Quan , L. Wang , Q. Wen , D. Lu , J. Wu , L. Jiang , W. Guo , Nat. Commun. 2019, 10, 1171. 30862778 10.1038/s41467-019-09178-x PMC6414642

[71] X. Lv , Y. Huang , Z. Liu , J. Tian , Y. Wang , Y. Ma , J. Liang , S. Fu , X. Wan , Y. Chen , Small 2009, 5, 1682. 19360726 10.1002/smll.200900044

[72] S. Ghosh , B. K. Sarker , A. Chunder , L. Zhai , S. I. Khondaker , Appl. Phys. Lett. 2010, 96, 163109.

[73] M. A. S. Mohammad Haniff , N. H. Zainal Ariffin , S. M. Hafiz , P. C. Ooi , M. I. Syono , A. M. Hashim , ACS Appl. Mater. Interfaces 2019, 11, 4625. 30618229 10.1021/acsami.8b19043

[74] H. Tian , Y. Cao , J. Sun , J. He , RSC Adv. 2017, 7, 46536.

[75] Y. Cao , H. Yang , Y. Zhao , Y. Zhang , T. Ren , B. Jin , J. He , J.‐L. Sun , ACS Photonics 2017, 4, 2797.

[76] R. Feng , Y. Zhang , L. Hu , J. Chen , M. Zaheer , Z.‐J. Qiu , P. Tian , C. Cong , Q. Nie , W. Jin , R. Liu , Appl. Phys. Express 2018, 11, 015101.

[77] Z. Gao , Z. Jin , Q. Ji , Y. Tang , J. Kong , L. Zhang , Y. Li , Carbon 2018, 128, 117.

[78] I. K. Moon , B. Ki , S. Yoon , J. Choi , J. Oh , Sci. Rep. 2016, 6, 33525. 27634110 10.1038/srep33525 PMC5025851

[79] H. Chang , Z. Sun , M. Saito , Q. Yuan , H. Zhang , J. Li , Z. Wang , T. Fujita , F. Ding , Z. Zheng , F. Yan , H. Wu , M. Chen , Y. Ikuhara , ACS Nano 2013, 7, 6310. 23782028 10.1021/nn4023679

[80] H. Chang , Z. Sun , Q. Yuan , F. Ding , X. Tao , F. Yan , Z. Zheng , Adv. Mater. 2010, 22, 4872. 20827686 10.1002/adma.201002229

[81] B. Chitara , S. B. Krupanidhi , C. N. R. Rao , Appl. Phys. Lett. 2011, 99, 113114.

[82] H. Liang , AIP Adv. 2014, 4, 107131.

[83] P. Sahatiya , S. K. Puttapati , V. V. S. S. Srikanth , S. Badhulika , Flexible Printed Electron. 2016, 1, 025006.

[84] Y. Cao , Y. Zhao , Y. Wang , Y. Zhang , J. Wen , Z. Zhao , L. Zhu , Carbon 2019, 144, 193.

[85] X. He , T. Tang , F. Liu , N. Tang , X. Li , Y. Du , Carbon 2015, 94, 1037.

[86] A. Radoi , A. Iordanescu , A. Cismaru , M. Dragoman , D. Dragoman , Nanotechnology 2010, 21, 455202. 20947946 10.1088/0957-4484/21/45/455202

[87] A. Kumar , S. Husale , A. K. Srivastava , P. K. Dutta , A. Dhar , Nanoscale 2014, 6, 8192. 24926960 10.1039/c4nr00916a

[88] J. Zhu , L. Zhang , W. Wu , Y. Cao , J. He , Int. J. Nanosci. 2014, 13, 1460008.

[89] J.‐Y. Liu , X.‐X. Yu , G.‐H. Zhang , Y.‐K. Wu , K. Zhang , N. Pan , X.‐P. Wang , Chin. J. Chem. Phys. 2013, 26, 225.

[90] S. Liu , B. Li , H. Kan , H. Liu , B. Xie , X. Zhu , Y. Hu , S. Jiang , J. Mater. Sci.: Mater. Electron. 2017, 28, 9403.

[91] K. K. Manga , S. Wang , M. Jaiswal , Q. Bao , K. P. Loh , Adv. Mater. 2010, 22, 5265. 20976829 10.1002/adma.201002939

[92] J. Zhu , Y. Cao , J. He , J. Colloid Interface Sci. 2014, 420, 119. 24559709 10.1016/j.jcis.2014.01.015

[93] P. Afzali , Y. Abdi , E. Arzi , J. Nanopart. Res. 2014, 16, 2659.

[94] C. Zhou , X. Wang , X. Kuang , S. Xu , J. Micromech. Microeng. 2016, 26, 075003.

[95] X. Geng , L. Niu , Z. Xing , R. Song , G. Liu , M. Sun , G. Cheng , H. Zhong , Z. Liu , Z. Zhang , L. Sun , H. Xu , L. Lu , L. Liu , Adv. Mater. 2010, 22, 638. 20217764 10.1002/adma.200902871

[96] Y. Lin , K. Zhang , W. Chen , Y. Liu , Z. Geng , J. Zeng , N. Pan , L. Yan , X. Wang , J. G. Hou , ACS Nano 2010, 4, 3033. 20499858 10.1021/nn100134j

[97] K. Yu , G. Lu , S. Mao , K. Chen , H. Kim , Z. Wen , J. Chen , ACS Appl. Mater. Interfaces 2011, 3, 2703. 21650220 10.1021/am200494v

[98] S. Ghosh , T. Pal , D. Joung , S. I. Khondaker , Appl. Phys. A: Mater. Sci. Process. 2012, 107, 995.

[99] R. Yousefi , M. R. Mahmoudian , A. Sa΄Aedi , M. Cheraghizade , F. Jamali‐Sheini , M. Azarang , Ceram. Int. 2016, 42, 15209.

[100] X. Song , Y. Zhang , H. Zhang , Y. Yu , M. Cao , Y. Che , H. Dai , J. Yang , X. Ding , J. Yao , Nanotechnology 2017, 28, 145201. 28184032 10.1088/1361-6528/aa5faf

[101] Z. Zhan , L. Zheng , Y. Pan , G. Sun , L. Li , J. Mater. Chem. 2012, 22, 2589.

[102] Y. Su , X. Lu , M. Xie , H. Geng , H. Wei , Z. Yang , Y. Zhang , Nanoscale 2013, 5, 8889. 23907643 10.1039/c3nr02992a

[103] G. Jiang , Y. Su , M. Li , J. Hu , B. Zhao , Z. Yang , H. Wei , RSC Adv. 2016, 6, 97861.

[104] L. Heshmatynezhad , F. Jamali‐Sheini , A. Monshi , Mater. Res. Express 2019, 6, 086332.

[105] D. Shao , M. Yu , J. Lian , S. Sawyer , Nanotechnology 2013, 24, 295701. 23799662 10.1088/0957-4484/24/29/295701

[106] L. Fan , Y. Tao , X. Wu , Z. Wu , J. Wu , Mater. Res. Bull. 2017, 93, 21.

[107] M. Kamalanathan , S. Karuppusamy , R. Sivakumar , R. Gopalakrishnan , J. Mater. Sci. 2015, 50, 8029.

[108] S. Ibrahim , K. Chakraborty , T. Pal , S. Ghosh , J. Mater. Eng. Perform. 2018, 27, 2629.

[109] Y. M. Song , M. Yoon , S. Y. Jang , D. M. Jang , Y. J. Cho , C. H. Kim , J. Park , E. H. Cha , J. Phys. Chem. C 2011, 115, 15311.

[110] Z. Tao , Y.‐A. Huang , X. Liu , J. Chen , W. Lei , X. Wang , L. Pan , J. Pan , Q. Huang , Z. Zhang , Nano‐Micro Lett. 2016, 8, 247. 10.1007/s40820-016-0083-7 PMC6223684 30460284

[111] R. Roy , N. S. Das , D. Sen , S. Saha , K. K. Chattopadhyay , Appl. Phys. A: Mater. Sci. Process. 2018, 124, 45.

[112] R. F. Dezfuly , R. Yousefi , F. Jamali‐Sheini , Ceram. Int. 2016, 42, 7455.

[113] A. Kharatzadeh , F. Jamali‐Sheini , R. Yousefi , Mater. Des. 2016, 107, 47.

[114] X. Liu , H. Du , X. W. Sun , RSC Adv. 2014, 4, 5136.

[115] H. Chang , Z. Sun , K. Y.‐F. Ho , X. Tao , F. Yan , W.‐M. Kwok , Z. Zheng , Nanoscale 2011, 3, 258. 20976323 10.1039/c0nr00588f

[116] Q. Lu , X. Pan , W. Wang , Y. Zhou , Z. Ye , Appl. Phys. A: Mater. Sci. Process. 2018, 124, 733.

[117] D. Shao , M. Yu , H. Sun , T. Hu , J. Lian , S. Sawyer , Nanoscale 2013, 5, 3664. 23552899 10.1039/c3nr00369h

[118] T. V. Tam , S. H. Hur , J. S. Chung , W. M. Choi , Sens. Actuators, A 2015, 233, 368.

[119] D. T. Thanh , K. B. Ko , Z. Khurelbaatar , C.‐J. Choi , C.‐H. Hong , T. V. Cuong , Mater. Res. Bull. 2017, 91, 49.

[120] M. S. Alam , M. A. Boby , F. A. Chowdhury , H. Albrithen , M. A. Hossain , RSC Adv. 2019, 9, 18996. 10.1039/c9ra01894h PMC9064949 35516900

[121] M. He , Y. Chen , H. Liu , J. Wang , X. Fang , Z. Liang , Chem. Commun. 2015, 51, 9659. 10.1039/c5cc02282g 25977949

[122] X. Tang , Z. Zu , Z. Zang , Z. Hu , W. Hu , Z. Yao , W. Chen , S. Li , S. Han , M. Zhou , Sens. Actuators, B 2017, 245, 435.

[123] X. Liu , T. Xu , Y. Li , Z. Zang , X. Peng , H. Wei , W. Zha , F. Wang , Sol. Energy Mater. Sol. Cells 2018, 187, 249.

[124] J. Liu , Q. Huang , K. Zhang , Y. Xu , M. Guo , Y. Qian , Z. Huang , F. Lai , L. Lin , Nanoscale Res. Lett. 2017, 12, 259. 28395477 10.1186/s11671-017-2021-0 PMC5383919

[125] M. Javadi , M. Gholami , Y. Abdi , J. Mater. Chem. C 2018, 6, 8444.

[126] A. Chunder , T. Pal , S. I. Khondaker , L. Zhai , J. Phys. Chem. C 2010, 114, 15129.

[127] H. Lin , Z. Xu , L. Zhang , X. Yang , Q. Ju , L. Xue , J. Zhou , S. Zhuo , Y. Wu , New J. Chem. 2017, 41, 4302.

[128] J. Sun , L. Xiao , D. Meng , J. Geng , Y. Huang , Chem. Commun. 2013, 49, 5538. 10.1039/c3cc40563j 23586076

[129] T. Q. Trung , S. Ramasundaram , N.‐E. Lee , Adv. Funct. Mater. 2015, 25, 1745.

[130] Q. Zhang , J. Jie , S. Diao , Z. Shao , Q. Zhang , L. Wang , W. Deng , W. Hu , H. Xia , X. Yuan , S.‐T. Lee , ACS Nano 2015, 9, 1561. 25625624 10.1021/acsnano.5b00437

[131] E. Zhang , T. Sun , B. Ge , W. Zhang , X. Gao , H. Jiang , Z. Li , G. Liu , J. Shen , Mater. Express 2018, 8, 105.

[132] S. Gao , L. Tang , J. Xiang , R. Ji , S. K. Lai , S. Yuan , S. P. Lau , New J. Chem. 2017, 41, 10447.

[133] R. Das , H. Sugimoto , M. Fujii , P. K. Giri , ACS Appl. Mater. Interfaces 2020, 12, 4755. 31914727 10.1021/acsami.9b19067

[134] K. Rahimi , A. Yazdani , M. Ahmadirad , Mater. Des. 2018, 140, 222.

[135] K. B. Ko , B. D. Ryu , M. Han , C.‐H. Hong , D. A. Dinh , T. V. Cuong , Appl. Surf. Sci. 2019, 481, 524.

[136] H. Chang , J. Cheng , X. Liu , J. Gao , M. Li , J. Li , X. Tao , F. Ding , Z. Zheng , Chem. ‐ Eur. J. 2011, 17, 8896. 21714019 10.1002/chem.201100699

[137] M.‐K. Dai , Y.‐R. Liou , J.‐T. Lian , T.‐Y. Lin , Y.‐F. Chen , ACS Photonics 2015, 2, 1057.

[138] K. K. Manga , J. Wang , M. Lin , J. Zhang , M. Nesladek , V. Nalla , W. Ji , K. P. Loh , Adv. Mater. 2012, 24, 1697. 22378495 10.1002/adma.201104399

[139] A. J. K. Al‐Alwani , A. S. Chumakov , O. A. Shinkarenko , I. A. Gorbachev , M. V. Pozharov , S. Venig , E. G. Glukhovskoy , Appl. Surf. Sci. 2017, 424, 222.

[140] P. Sahatiya , S. Badhulika , Nanotechnology 2017, 28, 455204. 29039356 10.1088/1361-6528/aa8587

[141] M. Döbbelin , A. Ciesielski , S. Haar , S. Osella , M. Bruna , A. Minoia , L. Grisanti , T. Mosciatti , F. Richard , E. A. Prasetyanto , L. De Cola , V. Palermo , R. Mazzaro , V. Morandi , R. Lazzaroni , A. C. Ferrari , D. Beljonne , P. Samorì , Nat. Commun. 2016, 7, 11090. 27052205 10.1038/ncomms11090 PMC4829665

[142] M. Zhang , J. T. W. Yeow , Carbon 2020, 156, 339.

[143] Y. Xie , M. Han , R. Wang , H. Zobeiri , X. Deng , P. Zhang , X. Wang , ACS Nano 2019, 13, 5385. 30998848 10.1021/acsnano.9b00031

[144] Y. Li , Y. Zhang , Y. Yu , Z. Chen , Q. Li , T. Li , J. Li , H. Zhao , Q. Sheng , F. Yan , Z. Ge , Y. Ren , Y. Chen , J. Yao , Photonics Res. 2020, 8, 368.

[145] C.‐X. Hu , Q. Xiao , Y.‐Y. Ren , M. Zhao , G.‐H. Dun , H.‐R. Wu , X.‐Y. Li , Q.‐Q. Yang , B. Sun , Y. Peng , F. Yan , Q. Wang , H.‐L. Zhang , Adv. Funct. Mater. 2018, 28, 1805311.

[146] G. Zhou , Z. Li , Y. Ge , H. Zhang , Z. Sun , Nanoscale Adv. 2020, 2, 1059. 10.1039/c9na00528e PMC9416809 36133069

[147] Z. Jia , J. Xiang , C. Mu , F. Wen , R. Yang , C. Hao , Z. Liu , J. Mater. Sci. 2017, 52, 11506.

[148] Q. Xiao , C.‐X. Hu , H.‐R. Wu , Y.‐Y. Ren , X.‐Y. Li , Q.‐Q. Yang , G.‐H. Dun , Z.‐P. Huang , Y. Peng , F. Yan , Q. Wang , H.‐L. Zhang , Nanoscale Horiz. 2020, 5, 124.

[149] Z. Liu , Y. Zhu , J. K. El‐Demellawi , D. B. Velusamy , A. M. El‐Zohry , O. M. Bakr , O. F. Mohammed , H. N. Alshareef , ACS Energy Lett. 2019, 4, 2315.

[150] G. Cunningham , U. Khan , C. Backes , D. Hanlon , D. Mccloskey , J. F. Donegan , J. N. Coleman , J. Mater. Chem. C 2013, 1, 6899.

[151] J. Li , M. M. Naiini , S. Vaziri , M. C. Lemme , M. Östling , Adv. Funct. Mater. 2014, 24, 6524.

[152] D. J. Finn , M. Lotya , G. Cunningham , R. J. Smith , D. Mccloskey , J. F. Donegan , J. N. Coleman , J. Mater. Chem. C 2014, 2, 925.

[153] D. Mcmanus , S. Vranic , F. Withers , V. Sanchez‐Romaguera , M. Macucci , H. Yang , R. Sorrentino , K. Parvez , S.‐K. Son , G. Iannaccone , K. Kostarelos , G. Fiori , C. Casiraghi , Nat. Nanotechnol. 2017, 12, 343. 28135260 10.1038/nnano.2016.281

[154] K. Lobo , S. Trivedi , H. S. S. R. Matte , Nanoscale 2019, 11, 10746. 31120460 10.1039/c9nr02019e

[155] J.‐W. T. Seo , J. Zhu , V. K. Sangwan , E. B. Secor , S. G. Wallace , M. C. Hersam , ACS Appl. Mater. Interfaces 2019, 11, 5675. 30693759 10.1021/acsami.8b19817

[156] G. Cunningham , D. Hanlon , N. Mcevoy , G. S. Duesberg , J. N. Coleman , Nanoscale 2015, 7, 198. 25408303 10.1039/c4nr04951a

[157] D. Mcmanus , A. Dal Santo , P. B. Selvasundaram , R. Krupke , A. Libassi , C. Casiraghi , Flexible Printed Electron. 2018, 3, 034005.

[158] M. J. Park , K. Park , H. Ko , Appl. Surf. Sci. 2018, 448, 64.

[159] S. Mukherjee , S. Jana , T. K. Sinha , S. Das , S. K. Ray , Nanoscale Adv. 2019, 1, 3279. 10.1039/c9na00302a PMC9419818 36133580

[160] J. Schornbaum , B. Winter , S. P. Schießl , F. Gannott , G. Katsukis , D. M. Guldi , E. Spiecker , J. Zaumseil , Adv. Funct. Mater. 2014, 24, 5798.

[161] D. B. Velusamy , M. d. A. Haque , M. R. Parida , F. Zhang , T. Wu , O. F. Mohammed , H. N. Alshareef , Adv. Funct. Mater. 2017, 27, 1605554.

[162] D. B. Velusamy , R. H. Kim , S. Cha , J. Huh , R. Khazaeinezhad , S. H. Kassani , G. Song , S. M. Cho , S. H. Cho , I. Hwang , J. Lee , K. Oh , H. Choi , C. Park , Nat. Commun. 2015, 6, 8063. 26333531 10.1038/ncomms9063 PMC4569699

[163] P. Pataniya , C. K. Zankat , M. Tannarana , C. K. Sumesh , S. Narayan , G. K. Solanki , K. D. Patel , V. M. Pathak , P. K. Jha , ACS Appl. Nano Mater. 2019, 2, 2758.

[164] J. Kang , V. K. Sangwan , H.‐S. Lee , X. Liu , M. C. Hersam , ACS Photonics 2018, 5, 3996.

[165] J. Kang , S. A. Wells , V. K. Sangwan , D. Lam , X. Liu , J. Luxa , Z. Sofer , M. C. Hersam , Adv. Mater. 2018, 30, 1802990. 10.1002/adma.201802990 30095182

[166] N. Curreli , M. Serri , D. Spirito , E. Lago , E. Petroni , B. Martín‐García , A. Politano , B. Gürbulak , S. Duman , R. Krahne , V. Pellegrini , F. Bonaccorso , Adv. Funct. Mater. 2020, 30, 1908427.

[167] H. Shi , M. Li , A. Shaygan Nia , M. Wang , S. Park , Z. Zhang , M. R. Lohe , S. Yang , X. Feng , Adv. Mater. 2020, 32, 1907244. 10.1002/adma.201907244 31944431

[168] S. Liu , Z. Huang , H. Qiao , R. Hu , Q. Ma , K. Huang , H. Li , X. Qi , Nanoscale Adv. 2020, 2, 906. 10.1039/c9na00745h PMC9418427 36133254

[169] S. Chertopalov , V. N. Mochalin , ACS Nano 2018, 12, 6109. 29883092 10.1021/acsnano.8b02379

[170] A. Pan , X. Ma , S. Huang , Y. Wu , M. Jia , Y. Shi , Y. Liu , P. Wangyang , L. He , Y. Liu , J. Phys. Chem. Lett. 2019, 10, 6590. 31596093 10.1021/acs.jpclett.9b02605

[171] S. Guo , S. Kang , S. Feng , W. Lu , J. Phys. Chem. C 2020, 124, 4764.

[172] D. B. Velusamy , J. K. El‐Demellawi , A. M. El‐Zohry , A. Giugni , S. Lopatin , M. N. Hedhili , A. E. Mansour , E. D. Fabrizio , O. F. Mohammed , H. N. Alshareef , Adv. Mater. 2019, 31, 1807658. 10.1002/adma.201807658 31222823

[173] K. Lee , H.‐Y. Kim , M. Lotya , J. N. Coleman , G.‐T. Kim , G. S. Duesberg , Adv. Mater. 2011, 23, 4178. 21823176 10.1002/adma.201101013

[174] R. J. Smith , P. J. King , M. Lotya , C. Wirtz , U. Khan , S. De , A. O'neill , G. S. Duesberg , J. C. Grunlan , G. Moriarty , J. Chen , J. Wang , A. I. Minett , V. Nicolosi , J. N. Coleman , Adv. Mater. 2011, 23, 3944. 21796689 10.1002/adma.201102584

[175] P. May , U. Khan , J. M. Hughes , J. N. Coleman , J. Phys. Chem. C 2012, 116, 11393.

[176] Y. Hernandez , V. Nicolosi , M. Lotya , F. M. Blighe , Z. Sun , S. De , I. T. Mcgovern , B. Holland , M. Byrne , Y. K. Gun'ko , J. J. Boland , P. Niraj , G. Duesberg , S. Krishnamurthy , R. Goodhue , J. Hutchison , V. Scardaci , A. C. Ferrari , J. N. Coleman , Nat. Nanotechnol. 2008, 3, 563. 18772919 10.1038/nnano.2008.215

[177] Y. Zhang , Y. Xu , J. Zhu , L. Li , X. Du , X. Sun , Carbon 2018, 127, 392.

[178] C.‐Y. Su , A.‐Y. Lu , Y. Xu , F.‐R. Chen , A. N. Khlobystov , L.‐J. Li , ACS Nano 2011, 5, 2332. 21309565 10.1021/nn200025p

[179] K. Parvez , R. Li , S. R. Puniredd , Y. Hernandez , F. Hinkel , S. Wang , X. Feng , K. Müllen , ACS Nano 2013, 7, 3598. 23531157 10.1021/nn400576v

[180] Z. Zeng , Z. Yin , X. Huang , H. Li , Q. He , G. Lu , F. Boey , H. Zhang , Angew. Chem., Int. Ed. 2011, 50, 11093. 10.1002/anie.201106004 22021163

[181] K. R. Paton , E. Varrla , C. Backes , R. J. Smith , U. Khan , A. O'neill , C. Boland , M. Lotya , O. M. Istrate , P. King , T. Higgins , S. Barwich , P. May , P. Puczkarski , I. Ahmed , M. Moebius , H. Pettersson , E. Long , J. Coelho , S. E. O'brien , E. K. Mcguire , B. M. Sanchez , G. S. Duesberg , N. Mcevoy , T. J. Pennycook , C. Downing , A. Crossley , V. Nicolosi , J. N. Coleman , Nat. Mater. 2014, 13, 624. 24747780 10.1038/nmat3944

[182] S. Biccai , S. Barwich , D. Boland , A. Harvey , D. Hanlon , N. Mcevoy , J. N. Coleman , 2D Mater. 2019, 6, 015008.

[183] H. Yuan , X. Liu , L. Ma , P. Gong , Z. Yang , H. Wang , J. Wang , S. Yang , RSC Adv. 2016, 6, 82763.

[184] F. Xu , B. Ge , J. Chen , A. Nathan , L. L. Xin , H. Ma , H. Min , C. Zhu , W. Xia , Z. Li , S. Li , K. Yu , L. Wu , Y. Cui , L. Sun , Y. Zhu , 2D Mater. 2016, 3, 025005.

[185] Y. Jung , Y. Zhou , J. J. Cha , Inorg. Chem. Front. 2016, 3, 452.

[186] A. M. Abdelkader , A. J. Cooper , R. A. W. Dryfe , I. A. Kinloch , Nanoscale 2015, 7, 6944. 25703415 10.1039/c4nr06942k

[187] J. N. Coleman , Adv. Funct. Mater. 2009, 19, 3680.

[188] H. Tao , Y. Zhang , Y. Gao , Z. Sun , C. Yan , J. Texter , Phys. Chem. Chem. Phys. 2017, 19, 921. 27976772 10.1039/c6cp06813h

[189] H. A. Becerril , J. Mao , Z. Liu , R. M. Stoltenberg , Z. Bao , Y. Chen , ACS Nano 2008, 2, 463. 19206571 10.1021/nn700375n

[190] Z. Liu , Q. Liu , Y. i Huang , Y. Ma , S. Yin , X. Zhang , W. Sun , Y. Chen , Adv. Mater. 2008, 20, 3924.

[191] W. S. Hummers , R. E. Offeman , J. Am. Chem. Soc. 1958, 80, 1339.

[192] M. A. Lukowski , A. S. Daniel , F. Meng , A. Forticaux , L. Li , S. Jin , J. Am. Chem. Soc. 2013, 135, 10274. 23790049 10.1021/ja404523s

[193] M. Naguib , M. Kurtoglu , V. Presser , J. Lu , J. Niu , M. Heon , L. Hultman , Y. Gogotsi , M. W. Barsoum , Adv. Mater. 2011, 23, 4248. 21861270 10.1002/adma.201102306

[194] M. Lotya , P. J. King , U. Khan , S. De , J. N. Coleman , ACS Nano 2010, 4, 3155. 20455583 10.1021/nn1005304

[195] Y. Arao , M. Kubouchi , Carbon 2015, 95, 802.

[196] T. J. Mason , J. P. Lorimer , Applied Sonochemistry: Uses of Power Ultrasound in Chemistry and Processing, Wiley‐VCH Verlag, Weinheim, Germany 2002.

[197] C. E. Hamilton , J. R. Lomeda , Z. Sun , J. M. Tour , A. R. Barron , Nano Lett. 2009, 9, 3460. 19645460 10.1021/nl9016623

[198] A. B. Bourlinos , V. Georgakilas , R. Zboril , T. A. Steriotis , A. K. Stubos , Small 2009, 5, 1841. 19408256 10.1002/smll.200900242

[199] U. Khan , A. O'neill , M. Lotya , S. De , J. N. Coleman , Small 2010, 6, 864. 20209652 10.1002/smll.200902066

[200] U. Khan , H. Porwal , A. O. Neill , P. May , J. N. Coleman , ACS Nano 2011, 27, 9077. 10.1021/la201797h 21675749

[201] M. Lotya , Y. Hernandez , P. J. King , R. J. Smith , V. Nicolosi , L. S. Karlsson , F. M. Blighe , S. De , Z. Wang , I. T. Mcgovern , G. S. Duesberg , J. N. Coleman , J. Am. Chem. Soc. 2009, 131, 3611. 19227978 10.1021/ja807449u

[202] L. Guardia , M. J. Fernández‐Merino , J. I. Paredes , P. Solís‐Fernández , S. Villar‐Rodil , A. Martínez‐Alonso , J. M. D. Tascón , Carbon 2011, 49, 1653.

[203] J. Geng , B.‐S. Kong , S. B. Yang , H.‐T. Jung , Chem. Commun. 2010, 46, 5091. 10.1039/c001609h 20549007

[204] X. Zhou , T. Wu , K. Ding , B. Hu , M. Hou , B. Han , Chem. Commun. 2010, 46, 386. 10.1039/b914763b 20066300

[205] X. Wang , P. F. Fulvio , G. A. Baker , G. M. Veith , R. R. Unocic , S. M. Mahurin , M. Chi , S. Dai , Chem. Commun. 2010, 46, 4487. 10.1039/c0cc00799d 20485780

[206] M. B. Dines , Mater. Res. Bull. 1975, 10, 287.

[207] B. K. Miremadi , S. R. Morrison , J. Appl. Phys. 1988, 63, 4970.

[208] N. I. Kovtyukhova , Y. Wang , A. Berkdemir , R. Cruz‐Silva , M. Terrones , V. H. Crespi , T. E. Mallouk , Nat. Chem. 2014, 6, 957. 25343599 10.1038/nchem.2054

[209] O. Mashtalir , M. Naguib , V. N. Mochalin , Y. Dall'agnese , M. Heon , M. W. Barsoum , Y. Gogotsi , Nat. Commun. 2013, 4, 1716. 23591883 10.1038/ncomms2664

[210] T. Hasan , F. Torrisi , Z. Sun , D. Popa , V. Nicolosi , G. Privitera , F. Bonaccorso , A. C. Ferrari , Phys. Status Solidi B 2010, 247, 2953.

[211] R. Hao , W. Qian , L. Zhang , Y. Hou , Chem. Commun. 2008, 6576. 10.1039/b816971c 19057784

[212] R. J. Smith , M. Lotya , J. N. Coleman , New J. Phys. 2010, 12, 125008.

[213] D. Nuvoli , L. Valentini , V. Alzari , S. Scognamillo , S. B. Bon , M. Piccinini , J. Illescas , A. Mariani , J. Mater. Chem. 2011, 21, 3428.

[214] H.‐L. Tsai , J. Heising , J. L. Schindler , C. R. Kannewurf , M. G. Kanatzidis , Chem. Mater. 1997, 9, 879.

[215] R. A. Gordon , D. Yang , E. D. Crozier , D. T. Jiang , R. F. Frindt , Phys. Rev. B: Condens. Matter Mater. Phys. 2002, 65, 1254071.

[216] K. Zhou , Y. Shi , S. Jiang , L. Song , Y. Hu , Z. Gui , Mater. Res. Bull. 2013, 48, 2985.

[217] N. I. Kovtyukhova , Y. Wang , R. Lv , M. Terrones , V. H. Crespi , T. E. Mallouk , J. Am. Chem. Soc. 2013, 135, 8372. 23663202 10.1021/ja403197h

[218] P. Joensen , R. F. Frindt , S. R. Morrison , Mater. Res. Bull. 1986, 21, 457.

[219] M. G. Kanatzidis , R. Bissessur , D. C. Degroot , J. L. Schindler , C. R. Kannewurf , Chem. Mater. 1993, 5, 595.

[220] M. S. Dresselhaus , G. Dresselhaus , Adv. Phys. 2002, 51, 1.

[221] M. Naguib , V. N. Mochalin , M. W. Barsoum , Y. Gogotsi , Adv. Mater. 2014, 26, 992. 24357390 10.1002/adma.201304138

[222] M. Naguib , O. Mashtalir , J. Carle , V. Presser , J. Lu , L. Hultman , Y. Gogotsi , M. W. Barsoum , ACS Nano 2012, 6, 1322. 22279971 10.1021/nn204153h

[223] J. P. Mensing , T. Kerdcharoen , C. Sriprachuabwong , A. Wisitsoraat , D. Phokharatkul , T. Lomas , A. Tuantranont , J. Mater. Chem. 2012, 22, 17094.

[224] M. Zhou , J. Tang , Q. Cheng , G. Xu , P. Cui , L.‐C. Qin , Chem. Phys. Lett. 2013, 572, 61.

[225] K. Parvez , Z.‐S. Wu , R. Li , X. Liu , R. Graf , X. Feng , K. Müllen , J. Am. Chem. Soc. 2014, 136, 6083. 24684678 10.1021/ja5017156

[226] N. Liu , P. Kim , J. H. Kim , J. H. Ye , S. Kim , C. J. Lee , ACS Nano 2014, 8, 6902. 24937086 10.1021/nn5016242

[227] Y. Yang , F. Lu , Z. Zhou , W. Song , Q. Chen , X. Ji , Electrochim. Acta 2013, 113, 9.

[228] E. Varrla , C. Backes , K. R. Paton , A. Harvey , Z. Gholamvand , J. Mccauley , J. N. Coleman , Chem. Mater. 2015, 27, 1129.

[229] E. Varrla , K. R. Paton , C. Backes , A. Harvey , R. J. Smith , J. Mccauley , J. N. Coleman , Nanoscale 2014, 6, 11810. 25164103 10.1039/c4nr03560g

[230] J. Kang , V. K. Sangwan , J. D. Wood , M. C. Hersam , Acc. Chem. Res. 2017, 50, 943. 28240855 10.1021/acs.accounts.6b00643

[231] U. Khan , A. O'neill , H. Porwal , P. May , K. Nawaz , J. N. Coleman , Carbon 2012, 50, 470.

[232] C. Backes , R. J. Smith , N. Mcevoy , N. C. Berner , D. Mccloskey , H. C. Nerl , A. O'neill , P. J. King , T. Higgins , D. Hanlon , N. Scheuschner , J. Maultzsch , L. Houben , G. S. Duesberg , J. F. Donegan , V. Nicolosi , J. N. Coleman , Nat. Commun. 2014, 5, 4576. 25099520 10.1038/ncomms5576

[233] F. Bonaccorso , A. Bartolotta , J. N. Coleman , C. Backes , Adv. Mater. 2016, 28, 6136. 27273554 10.1002/adma.201506410

[234] F. Bonaccorso , A. Lombardo , T. Hasan , Z. Sun , L. Colombo , A. C. Ferrari , Mater. Today 2012, 15, 564.

[235] F. I. Alzakia , W. Sun , S. J. Pennycook , S. C. Tan , ACS Appl. Mater. Interfaces 2020, 12, 3096. 31847515 10.1021/acsami.9b14510

[236] C. Backes , B. M. Szydłowska , A. Harvey , S. Yuan , V. Vega‐Mayoral , B. R. Davies , P.‐L. Zhao , D. Hanlon , E. J. G. Santos , M. I. Katsnelson , W. J. Blau , C. Gadermaier , J. N. Coleman , ACS Nano 2016, 10, 1589. 26728793 10.1021/acsnano.5b07228

[237] C. Backes , K. R. Paton , D. Hanlon , S. Yuan , M. I. Katsnelson , J. Houston , R. J. Smith , D. Mccloskey , J. F. Donegan , J. N. Coleman , Nanoscale 2016, 8, 4311. 26838813 10.1039/c5nr08047a

[238] A. Griffin , A. Harvey , B. Cunningham , D. Scullion , T. Tian , C.‐J. Shih , M. Gruening , J. F. Donegan , E. J. G. Santos , C. Backes , J. N. Coleman , Chem. Mater. 2018, 30, 1998.

[239] A. A. Green , M. C. Hersam , Nano Lett. 2009, 9, 4031. 19780528 10.1021/nl902200b

[240] J. Zhu , J. Kang , J. Kang , D. Jariwala , J. D. Wood , J.‐W. T. Seo , K.‐S. Chen , T. J. Marks , M. C. Hersam , Nano Lett. 2015, 15, 7029. 26348822 10.1021/acs.nanolett.5b03075

[241] J. Kang , J.‐W. T. Seo , D. Alducin , A. Ponce , M. J. Yacaman , M. C. Hersam , Nat. Commun. 2014, 5, 5478. 25391315 10.1038/ncomms6478

[242] J. Kang , V. K. Sangwan , J. D. Wood , X. Liu , I. Balla , D. Lam , M. C. Hersam , Nano Lett. 2016, 16, 7216. 27700101 10.1021/acs.nanolett.6b03584

[243] G. Hu , J. Kang , L. W. T. Ng , X. Zhu , R. C. T. Howe , C. G. Jones , M. C. Hersam , T. Hasan , Chem. Soc. Rev. 2018, 47, 3265. 29667676 10.1039/c8cs00084k

[244] J. Li , M. C. Lemme , M. Östling , ChemPhysChem 2014, 15, 3427. 25169938 10.1002/cphc.201402103

[245] W. Huang , C. Xing , Y. Wang , Z. Li , L. Wu , D. Ma , X. Dai , Y. Xiang , J. Li , D. Fan , H. Zhang , Nanoscale 2018, 10, 2404. 29334393 10.1039/c7nr09046c

[246] S. K. Pandey , E. Ramya , J. K. S. Gangwar , P. Kaur , S. Kumar , D. N. Rao , S. M. Rao , AIP Conf. Proc. 2015, 1675, 020040.

[247] H. B. Sun , J. Yang , Y. Z. Zhou , N. Zhao , D. Li , Mater. Technol. 2014, 29, 14.

[248] E. Kymakis , E. Stratakis , M. M. Stylianakis , E. Koudoumas , C. Fotakis , Thin Solid Films 2011, 520, 1238.

[249] C.‐W. Liu , C. Wang , C.‐W. Liao , J. Golder , M.‐C. Tsai , H.‐T. Young , C.‐T. Chen , C.‐I. Wu , AIP Adv. 2018, 8, 045006.

[250] A. G. Kelly , T. Hallam , C. Backes , A. Harvey , A. S. Esmaeily , I. Godwin , J. Coelho , V. Nicolosi , J. Lauth , A. Kulkarni , S. Kinge , L. D. A. Siebbeles , G. S. Duesberg , J. N. Coleman , Science 2017, 356, 69. 28386010 10.1126/science.aal4062

[251] T. M. Higgins , S. Finn , M. Matthiesen , S. Grieger , K. Synnatschke , M. Brohmann , M. Rother , C. Backes , J. Zaumseil , Adv. Funct. Mater. 2019, 29, 1804387.

[252] S. Lim , B. Cho , J. Bae , A. R. Kim , K. H. Lee , S. H. Kim , M. G. Hahm , J. Nam , Nanotechnology 2016, 27, 435501. 27658490 10.1088/0957-4484/27/43/435501

[253] C. Zhao , L. Xing , J. Xiang , L. Cui , J. Jiao , H. Sai , Z. Li , F. Li , Particuology 2014, 17, 66.

[254] H. Liu , W. Song , Y. Yu , Q. Jiang , F. Pang , T. Wang , Photonic Sens. 2019, 9, 239.

[255] R. F. Hossain , I. G. Deaguero , T. Boland , A. B. Kaul , npj 2D Mater. Appl. 2017, 1, 28.

[256] H. Ahmad , H. Rashid , J. Mod. Opt. 2019, 66, 1836.

[257] A. V. Raval , I. A. Shaikh , V. M. Jain , N. M. Shastri , P. B. Patel , L. K. Saini , D. V. Shah , J. Nano‐ Electron. Phys. 2020, 12, 02010.

[258] E. Pineda , M. E. Nicho , P. K. Nair , H. Hu , Sol. Energy 2012, 86, 1017.

[259] X. Gao , G. Bian , J. Zhu , J. Mater. Chem. C 2019, 7, 12835.

[260] H. Yang , A. Giri , S. Moon , S. Shin , J.‐M. Myoung , U. Jeong , Chem. Mater. 2017, 29, 5772.

[261] P. Salles , D. Pinto , K. Hantanasirisakul , K. Maleski , C. E. Shuck , Y. Gogotsi , Adv. Funct. Mater. 2019, 29, 1809223.

[262] T. Radhika , U. M. Uzma Sulthana , K. G. Vasanthakumari , AIP Conf. Proc. 2020, 2263, 17436.

[263] D. Son , S. I. Chae , M. Kim , M. K. Choi , J. Yang , K. Park , V. S. Kale , J. H. Koo , C. Choi , M. Lee , J. H. Kim , T. Hyeon , D.‐H. Kim , Adv. Mater. 2016, 28, 9326. 27571382 10.1002/adma.201602391

[264] Z. Lin , Y. Huang , X. Duan , Nat. Electron. 2019, 2, 378.

[265] R. Nishikubo , A. Saeki , J. Phys. Chem. Lett. 2018, 9, 5392. 30183306 10.1021/acs.jpclett.8b02218

[266] C. Hollar , Z. Lin , M. Kongara , T. Varghese , C. Karthik , J. Schimpf , J. Eixenberger , P. H. Davis , Y. Wu , X. Duan , Y. Zhang , D. Estrada , Adv. Mater. Technol. 2020, 5, 2000600. 33738334 10.1002/admt.202000600 PMC7968868

[267] J. Kim , Y. D. Kim , D. G. Nam , J. Nanosci. Nanotechnol. 2013, 13, 3387. 23858864 10.1166/jnn.2013.7281

[268] R. Gusmão , Z. Sofer , P. Marvan , M. Pumera , Nanoscale 2019, 11, 9888. 31086894 10.1039/c9nr01876j

[269] P. Bondavalli , D. Pribat , P. Legagneux , M.‐B. Martin , L. Hamidouche , L. Qassym , G. Feugnet , A.‐F. Trompeta , C. A. Charitidis , J. Phys.: Mater. 2019, 2, 032002.

[270] M. Singh , H. M. Haverinen , P. Dhagat , G. E. Jabbour , Adv. Mater. 2010, 22, 673. 20217769 10.1002/adma.200901141

[271] F. Torrisi , T. Hasan , W. Wu , Z. Sun , A. Lombardo , T. S. Kulmala , G.‐W. Hsieh , S. Jung , F. Bonaccorso , P. J. Paul , D. Chu , A. C. Ferrari , ACS Nano 2012, 6, 2992. 22449258 10.1021/nn2044609

[272] G. Hu , T. Albrow‐Owen , X. Jin , A. Ali , Y. Hu , R. C. T. Howe , K. Shehzad , Z. Yang , X. Zhu , R. I. Woodward , T.‐C. Wu , H. Jussila , J.‐B. Wu , P. Peng , P.‐H. Tan , Z. Sun , E. J. R. Kelleher , M. Zhang , Y. Xu , T. Hasan , Nat. Commun. 2017, 8, 278. 28819184 10.1038/s41467-017-00358-1 PMC5561124

[273] P. He , J. R. Brent , H. Ding , J. Yang , D. J. Lewis , P. O'brien , B. Derby , Nanoscale 2018, 10, 5599. 29565064 10.1039/C7NR08115D

[274] J.‐U. Park , M. Hardy , S. J. Kang , K. Barton , K. Adair , D. K. Mukhopadhyay , C. Y. Lee , M. S. Strano , A. G. Alleyne , J. G. Georgiadis , P. M. Ferreira , J. A. Rogers , Nat. Mater. 2007, 6, 782. 17676047 10.1038/nmat1974

[275] M. S. Onses , E. Sutanto , P. M. Ferreira , A. G. Alleyne , J. A. Rogers , Small 2015, 11, 4237. 26122917 10.1002/smll.201500593

[276] Y. Han , J. Dong , J. Micro Nano‐Manuf. 2018, 6, 040802.

[277] K. Zhao , D. Wang , K. Li , C. Jiang , Y. Wei , J. Qian , L. Feng , Z. Du , Z. Xu , J. Liang , J. Electrochem. Soc. 2020, 167, 107508.

[278] F. I. Alzakia , W. Jonhson , J. Ding , S. C. Tan , ACS Appl. Mater. Interfaces 2020, 12, 28840. 32469199 10.1021/acsami.0c06279

[279] J. Kim , S. Kwon , D.‐H. Cho , B. Kang , H. Kwon , Y. Kim , S. O. Park , G. Y. Jung , E. Shin , W.‐G. Kim , H. Lee , G. H. Ryu , M. Choi , T. H. Kim , J. Oh , S. Park , S. K. Kwak , S. W. Yoon , D. Byun , Z. Lee , C. Lee , Nat. Commun. 2015, 6, 8294. 26369895 10.1038/ncomms9294 PMC4579837

[280] Y. Cao , J. Zhu , J. Xu , J. He , J.‐L. Sun , Y. Wang , Z. Zhao , Small 2014, 10, 2345. 24610715 10.1002/smll.201303339

[281] J. Yang , N. Huo , Y. Li , X.‐W. Jiang , T. Li , R. Li , F. Lu , C. Fan , B. Li , K. Nørgaard , B. W. Laursen , Z. Wei , J. Li , S.‐S. Li , Adv. Electron. Mater. 2015, 1, 1500267.

[282] H. Liu , Q. i Sun , J. Xing , Z. Zheng , Z. Zhang , Z. Lü , K. Zhao , ACS Appl. Mater. Interfaces 2015, 7, 6645. 25768384 10.1021/am509084r

[283] A. Radoi , M. Dragoman , D. Dragoman , Phys. E 2017, 85, 164.

[284] P. Xiao , J. Mao , K. Ding , W. Luo , W. Hu , X. Zhang , X. Zhang , J. Jie , Adv. Mater. 2018, 30, 1801729. 10.1002/adma.201801729 29923241

[285] B. Wang , Z. Huang , P. Tang , S. Luo , Y. Liu , J. Li , X. Qi , Nanotechnology 2020, 31, 115201. 31747652 10.1088/1361-6528/ab5970

[286] B. Wang , W. Zhu , P. Tang , X. Qi , Fullerenes, Nanotubes, Carbon Nanostruct. 2019, 27, 928.

[287] Z. Li , H. Qiao , Z. Guo , X. Ren , Z. Huang , X. Qi , S. C. Dhanabalan , J. S. Ponraj , D. u. Zhang , J. Li , J. Zhao , J. Zhong , H. Zhang , Adv. Funct. Mater. 2018, 28, 1705237.

[288] L. Ge , Q. Liu , N. Hao , W. Kun , J. Mater. Chem. B 2019, 7, 7283. 31720680 10.1039/c9tb01644a

[289] J. Wang , Z. Liu , Trends Anal. Chem. 2020, 133, 116089.

[290] F. H. L. Koppens , T. Mueller , P. h. Avouris , A. C. Ferrari , M. S. Vitiello , M. Polini , Nat. Nanotechnol. 2014, 9, 780. 25286273 10.1038/nnano.2014.215

[291] T. S. Kasirga , Thermal Conductivity Measurements in Atomically Thin Materials and Devices, Springer, Berlin, Germany 2020.

[292] R. M. Hill , Phys. Status Solidi 1976, 34, 601.

[293] F. Jabbarzadeh , M. Siahsar , M. Dolatyari , G. Rostami , A. Rostami , Appl. Phys. B: Lasers Opt. 2015, 120, 637.

[294] V. A. Kondrashov , N. S. Struchkov , R. Y. Rozanov , V. K. Nevolin , D. S. Kopylova , A. G. Nasibulin , Nanotechnology 2018, 29, 035301. 29182523 10.1088/1361-6528/aa9de1

[295] W. Dickerson , N. Hemsworth , P. Gaskell , E. Ledwosinska , T. Szkopek , Appl. Phys. Lett. 2015, 107, 243103.

[296] X. Lu , L. Sun , P. Jiang , X. Bao , Adv. Mater. 2019, 31, 1902044. 10.1002/adma.201902044 31483546

[297] N. W. Ashcroft , N. D. Mermin , Solid State Physics, Cengage Learning, Boston, MA 1976.

[298] M. Buscema , J. O. Island , D. J. Groenendijk , S. I. Blanter , G. A. Steele , H. S. J. Van Der Zant , A. Castellanos‐Gomez , Chem. Soc. Rev. 2015, 44, 3691. 25909688 10.1039/c5cs00106d

[299] H. Fang , W. Hu , Adv. Sci. 2017, 4, 1700323. 10.1002/advs.201700323 PMC5737233 29270342

[300] Q. Guo , A. Pospischil , M. Bhuiyan , H. Jiang , H. Tian , D. Farmer , B. Deng , C. Li , S.‐J. Han , H. Wang , Q. Xia , T.‐P. Ma , T. Mueller , F. Xia , Nano Lett. 2016, 16, 4648. 27332146 10.1021/acs.nanolett.6b01977

[301] G. Konstantatos , M. Badioli , L. Gaudreau , J. Osmond , M. Bernechea , F. P. G. De Arquer , F. Gatti , F. H. L. Koppens , Nat. Nanotechnol. 2012, 7, 363. 22562036 10.1038/nnano.2012.60

[302] K. Roy , M. Padmanabhan , S. Goswami , T. P. Sai , G. Ramalingam , S. Raghavan , A. Ghosh , Nat. Nanotechnol. 2013, 8, 826. 24141541 10.1038/nnano.2013.206

[303] L. J. Willis , J. A. Fairfield , T. Dadosh , M. D. Fischbein , M. Drndic , Nano Lett. 2009, 9, 4191. 19827798 10.1021/nl9024209

[304] U. N. Noumbé , C. Gréboval , C. Livache , T. Brule , B. Doudin , A. Ouerghi , E. Lhuillier , J.‐F. Dayen , Adv. Funct. Mater. 2019, 29, 1.

[305] A. Dorodnyy , Y. Salamin , P. Ma , J. Vukajlovic Plestina , N. Lassaline , D. Mikulik , P. Romero‐Gomez , A. Fontcuberta , I. Morral , J. Leuthold , IEEE J. Sel. Top. Quantum Electron. 2018, 24, 1.

[306] H. Liu , A. Pourret , P. Guyot‐Sionnest , ACS Nano 2010, 4, 5211. 20718437 10.1021/nn101376u

[307] A. J. Houtepen , D. Kockmann , D. Vanmaekelbergh , Nano Lett. 2008, 8, 3516. 18788827 10.1021/nl8020347

[308] S. Ghatak , A. Ghosh , Appl. Phys. Lett. 2013, 103, 122103.

[309] F. Mahvash , E. Paradis , D. Drouin , T. Szkopek , M. Siaj , Nano Lett. 2015, 15, 2263. 25730309 10.1021/nl504197c

[310] P. Mark , W. Helfrich , J. Appl. Phys. 1962, 33, 205.

[311] D. Joung , A. Chunder , L. Zhai , S. I. Khondaker , Appl. Phys. Lett. 2010, 97, 093105.

[312] V. Kumar , S. C. Jain , A. K. Kapoor , J. Poortmans , R. Mertens , J. Appl. Phys. 2003, 94, 1283.

[313] S. M. Sze , K. K. Ng , Y. Li , Physics of Semiconductor Devices Wiley, Weinheim, Germany 2007.

[314] Y. Liu , P. Stradins , S.‐H. Wei , Sci. Adv. 2016, 2, e1600069. 27152360 10.1126/sciadv.1600069 PMC4846439

[315] Y. Liu , Y. Huang , X. Duan , Nature 2019, 567, 323. 30894723 10.1038/s41586-019-1013-x

[316] K. S. Novoselov , Z. Jiang , Y. Zhang , S. V. Morozov , H. L. Stormer , U. Zeitler , J. C. Maan , G. S. Boebinger , P. Kim , A. K. Geim , Science 2007, 315, 1379. 17303717 10.1126/science.1137201

[317] Y. Zhang , Y.‐W. Tan , H. L. Stormer , P. Kim , Nature 2005, 438, 201. 16281031 10.1038/nature04235

[318] A. A. Balandin , S. Ghosh , W. Bao , I. Calizo , D. Teweldebrhan , F. Miao , C. N. Lau , Nano Lett. 2008, 8, 902. 18284217 10.1021/nl0731872

[319] G. ‐ H. Lee , R. C. Cooper , S. J. An , S. Lee , A. Van Der Zande , N. Petrone , A. G. Hammerberg , C. Lee , B. Crawford , W. Oliver , J. W. Kysar , J. Hone , Science 2013, 340, 1073. 23723231 10.1126/science.1235126

[320] J.‐C. Charlier , X. Gonze , J.‐P. Michenaud , Phys. Rev. B 1991, 43, 4579. 10.1103/physrevb.43.4579 9997825

[321] A. G. Ricciardulli , S. Yang , G.‐J. A. H. Wetzelaer , X. Feng , P. W. M. Blom , Adv. Funct. Mater. 2018, 28, 1706010.

[322] H. Lee , M. Kim , I. Kim , H. Lee , Adv. Mater. 2016, 28, 4541. 26823085 10.1002/adma.201505559

[323] C. O. Kim , S. W. Hwang , S. Kim , D. H. Shin , S. S. Kang , J. M. Kim , C. W. Jang , J. H. Kim , K. W. Lee , S.‐H. Choi , E. Hwang , Sci. Rep. 2014, 4, 5603. 24998800 10.1038/srep05603 PMC4083259

[324] Y. Cao , J. Zhu , J. Xu , J. He , Carbon 2014, 77, 1111.

[325] N. T. Shelke , B. R. Karche , Appl. Surf. Sci. 2017, 418, 374.

[326] H. Yang , Y. Cao , J. He , Y. Zhang , B. Jin , J.‐L. Sun , Y. Wang , Z. Zhao , Carbon 2017, 115, 561.

[327] R. Karimzadeh , M. Assar , M. Jahanbakhshian , Mater. Res. Bull. 2018, 100, 42.

[328] M. A. Khan , K. L. Kumawat , K. K. Nanda , S. B. Krupanidhi , J. Nanopart. Res. 2018, 20, 293.

[329] Abid , P. Sehrawat , S. S. Islam , P. Mishra , S. Ahmad , Sci. Rep. 2018, 8, 3537. 29476091 10.1038/s41598-018-21686-2 PMC5824820

[330] H. Y. Yang , H. J. Lee , Y. Jun , Y. J. Yun , Thin Solid Films 2020, 697, 137785.

[331] F. Xia , T. Mueller , Y.‐M. Lin , A. Valdes‐Garcia , P. Avouris , Nat. Nanotechnol. 2009, 4, 839. 19893532 10.1038/nnano.2009.292

[332] T. Mueller , F. Xia , P. Avouris , Nat. Photonics 2010, 4, 297.

[333] P. G. Collins , Science 2001, 292, 706. 11326094 10.1126/science.1058782

[334] S. K. Lai , L. Tang , Y. Y. Hui , C. M. Luk , S. P. Lau , J. Mater. Chem. C 2014, 2, 6971.

[335] F. A. Chowdhury , T. Mochida , J. Otsuki , M. S. Alam , Chem. Phys. Lett. 2014, 593, 198.

[336] G. E. Fernandes , J. H. Kim , D. Oller , J. Xu , Appl. Phys. Lett. 2015, 107, 111111.

[337] X. Li , X. Wang , L. Zhang , S. Lee , H. Dai , Science 2008, 319, 1229. 18218865 10.1126/science.1150878

[338] Y.‐W. Son , M. L. Cohen , S. G. Louie , Phys. Rev. Lett. 2006, 97, 216803. 17155765 10.1103/PhysRevLett.97.216803

[339] C. Gómez‐Navarro , R. T. Weitz , A. M. Bittner , M. Scolari , A. Mews , M. Burghard , K. Kern , Nano Lett. 2007, 7, 3499. 17944526 10.1021/nl072090c

[340] D. Pan , J. Zhang , Z. Li , M. Wu , Adv. Mater. 2010, 22, 734. 20217780 10.1002/adma.200902825

[341] S. Stankovich , D. A. Dikin , R. D. Piner , K. A. Kohlhaas , A. Kleinhammes , Y. Jia , Y. Wu , S. T. Nguyen , R. S. Ruoff , Carbon 2007, 45, 1558.

[342] L. Peng , L. Hu , X. Fang , Adv. Mater. 2013, 25, 5321. 24089350 10.1002/adma.201301802

[343] J.‐H. Zhang , T. Sun , A. Niu , Y.‐M. Tang , S. Deng , W. Luo , Q. Xu , D. Wei , D.‐S. Pei , Biomaterials 2017, 133, 49. 28433937 10.1016/j.biomaterials.2017.04.026

[344] T. Ghosh , E. Prasad , J. Phys. Chem. C 2015, 119, 2733. 10.1021/acs.jpca.5b08522 26580460

[345] M. A. Velasco‐Soto , S. A. Pérez‐García , J. Alvarez‐Quintana , Y. Cao , L. Nyborg , L. Licea‐Jiménez , Carbon 2015, 93, 967.

[346] H. Aguilar‐Bolados , A. Contreras‐Cid , M. Yazdani‐Pedram , G. Acosta‐Villavicencio , M. Flores , P. Fuentealba , A. Neira‐Carrillo , R. Verdejo , M. A. López‐Manchado , J. Colloid Interface Sci. 2018, 524, 219. 29655140 10.1016/j.jcis.2018.04.030

[347] W. Feng , P. Long , Y. Feng , Y. Li , Adv. Sci. 2016, 3, 1500413. 10.1002/advs.201500413 PMC5115570 27981018

[348] V. Mazánek , O. Jankovský , J. Luxa , D. Sedmidubský , Z. Janoušek , F. Šembera , M. Mikulics , Z. Sofer , Nanoscale 2015, 7, 13646. 26214601 10.1039/c5nr03243a

[349] V. Patil , A. Capone , S. Strauf , E.‐H. Yang , Sci. Rep. 2013, 3, 2791. 24071929 10.1038/srep02791 PMC3784941

[350] M. Freitag , T. Low , P. Avouris , Nano Lett. 2013, 13, 1644. 23452264 10.1021/nl4001037

[351] J. Wen , Y. Niu , P. Wang , M. Chen , W. Wu , Y. Cao , J.‐L. Sun , M. Zhao , D. Zhuang , Y. Wang , Carbon 2019, 153, 274.

[352] S. Pei , H.‐M. Cheng , Carbon 2012, 50, 3210.

[353] F. Hao , D. Fang , Z. Xu , Appl. Phys. Lett. 2011, 99, 041901.

[354] S. Evlashin , P. Dyakonov , R. Khmelnitsky , S. Dagesyan , A. Klokov , A. Sharkov , P. Timashev , S. Minaeva , K. Maslakov , S. Svyakhovskiy , N. Suetin , ACS Appl. Mater. Interfaces 2016, 8, 28880. 27704776 10.1021/acsami.6b10145

[355] P. Kang , M. C. Wang , P. M. Knapp , S. Nam , Adv. Mater. 2016, 28, 4639. 27061899 10.1002/adma.201600482

[356] E. Hutter , J. H. Fendler , Adv. Mater. 2004, 16, 1685.

[357] S. K. Ghosh , T. Pal , Chem. Rev. 2007, 107, 4797. 17999554 10.1021/cr0680282

[358] N. Toshima , T. Yonezawa , New J. Chem. 1998, 22, 1179.

[359] D. C. Look , C. Coşkun , B. Claflin , G. C. Farlow , Phys. B 2003, 340–342, 32.

[360] C. Soci , A. Zhang , B. Xiang , S. A. Dayeh , D. P. R. Aplin , J. Park , X. Y. Bao , Y. H. Lo , D. Wang , Nano Lett. 2007, 7, 1003. 17358092 10.1021/nl070111x

[361] A. Goudarzi , G. M. Aval , S. S. Park , M.‐C. Choi , R. Sahraei , M. H. Ullah , A. Avane , C.‐S. Ha , Chem. Mater. 2009, 21, 2375.

[362] R. Yousefi , A. K. Zak , F. Jamali‐Sheini , Mater. Sci. Semicond. Process. 2013, 16, 771.

[363] J. Suehiro , N. Nakagawa , S.‐I. Hidaka , M. Ueda , K. Imasaka , M. Higashihata , T. Okada , M. Hara , Nanotechnology 2006, 17, 2567. 21727506 10.1088/0957-4484/17/10/021

[364] M. Asad , M. Fathipour , M. H. Sheikhi , M. Pourfath , Sens. Actuators, A 2014, 220, 213.

[365] Y. Wu , C. Wadia , W. Ma , B. Sadtler , A. P. Alivisatos , Nano Lett. 2008, 8, 2345. 18651779 10.1021/nl801817d

[366] B. G. Kumar , B. Srinivas , K. Muralidharan , Mater. Res. Bull. 2017, 89, 108.

[367] X. An , J. C. Yu , Y. Wang , Y. Hu , X. Yu , G. Zhang , J. Mater. Chem. 2012, 22, 8525.

[368] K. Huang , Q. Zhang , F. Yang , D. He , Nano Res. 2010, 3, 281.

[369] W.‐W. Xiong , J.‐Q. Chen , X.‐C. Wu , J.‐J. Zhu , J. Mater. Chem. C 2014, 2, 7392.

[370] D. M. Berg , R. Djemour , L. Gütay , G. Zoppi , S. Siebentritt , P. J. Dale , Thin Solid Films 2012, 520, 6291.

[371] J. S. Park , W.‐J. Maeng , H.‐S. Kim , J.‐S. Park , Thin Solid Films 2012, 520, 1679.

[372] M. A. Hossain , S. Islam , F. A. Chowdhury , T. G. Mohiuddin , K. Uchida , T. Tamura , K. Sugawa , T. Mochida , J. Otsuki , M. S. Alam , Fullerenes, Nanotubes, Carbon Nanostruct. 2016, 24, 43.

[373] H.‐S. Kim , C.‐R. Lee , J.‐H. Im , K.‐B. Lee , T. Moehl , A. Marchioro , S.‐J. Moon , R. Humphry‐Baker , J.‐H. Yum , J. E. Moser , M. Grätzel , N.‐G. Park , Sci. Rep. 2012, 2, 591. 22912919 10.1038/srep00591 PMC3423636

[374] C. Wehrenfennig , G. E. Eperon , M. B. Johnston , H. J. Snaith , L. M. Herz , Adv. Mater. 2014, 26, 1584. 24757716 10.1002/adma.201305172 PMC4722848

[375] X. Hu , X. Zhang , L. Liang , J. Bao , S. Li , W. Yang , Y. Xie , Adv. Funct. Mater. 2014, 24, 7373.

[376] H.‐R. Xia , J. Li , W.‐T. Sun , L.‐M. Peng , Chem. Commun. 2014, 50, 13695. 10.1039/c4cc05960c 25247451

[377] X. Tang , Z. Zu , H. Shao , W. Hu , M. Zhou , M. Deng , W. Chen , Z. Zang , T. Zhu , J. Xue , Nanoscale 2016, 8, 15158. 27500438 10.1039/c6nr01828a

[378] L. Lv , Y. Xu , H. Fang , W. Luo , F. Xu , L. Liu , B. Wang , X. Zhang , D. Yang , W. Hu , A. Dong , Nanoscale 2016, 8, 13589. 27378539 10.1039/c6nr03428d

[379] M. Tegze , G. Faigel , Nature 1996, 380, 49.

[380] M. J. Yaffe , J. A. Rowlands , Phys. Med. Biol. 1997, 42, 1. 9015806 10.1088/0031-9155/42/1/001

[381] S. Yakunin , M. Sytnyk , D. Kriegner , S. Shrestha , M. Richter , G. J. Matt , H. Azimi , C. J. Brabec , J. Stangl , M. V. Kovalenko , W. Heiss , Nat. Photonics 2015, 9, 444. 28553368 10.1038/nphoton.2015.82 PMC5444515

[382] M. A. Franzman , C. W. Schlenker , M. E. Thompson , R. L. Brutchey , J. Am. Chem. Soc. 2010, 132, 4060. 20201510 10.1021/ja100249m

[383] W. J. Baumgardner , J. J. Choi , Y.‐F. Lim , T. Hanrath , J. Am. Chem. Soc. 2010, 132, 9519. 20578741 10.1021/ja1013745

[384] J. Henry , J. Livingstone , Adv. Mater. 2001, 13, 1022.

[385] A. B. P. Lever , M. R. Hempstead , C. C. Leznoff , W. Liu , M. Melnik , W. A. Nevin , P. Seymour , Pure Appl. Chem. 1986, 58, 1467.

[386] Q. Su , S. Pang , V. Alijani , C. Li , X. Feng , K. Müllen , Adv. Mater. 2009, 21, 3191.

[387] W. Luo , Y. Feng , C. Cao , M. Li , E. Liu , S. Li , C. Qin , W. Hu , W. Feng , J. Mater. Chem. A 2015, 3, 11787.

[388] N. Peimyoo , J. Li , J. Shang , X. Shen , C. Qiu , L. Xie , W. Huang , T. Yu , ACS Nano 2012, 6, 8878. 22966836 10.1021/nn302876w

[389] M. Kaur , D. H. Choi , Chem. Soc. Rev. 2015, 44, 58. 25186723 10.1039/c4cs00248b

[390] F. Xia , H. Wang , D. Xiao , M. Dubey , A. Ramasubramaniam , Nat. Photonics 2014, 8, 899.

[391] D. Hanlon , C. Backes , E. Doherty , C. S. Cucinotta , N. C. Berner , C. Boland , K. Lee , A. Harvey , P. Lynch , Z. Gholamvand , S. Zhang , K. Wang , G. Moynihan , A. Pokle , Q. M. Ramasse , N. Mcevoy , W. J. Blau , J. Wang , G. Abellan , F. Hauke , A. Hirsch , S. Sanvito , D. D. O'regan , G. S. Duesberg , V. Nicolosi , J. N. Coleman , Nat. Commun. 2015, 6, 8563. 26469634 10.1038/ncomms9563 PMC4634220

[392] J. O. Island , G. A. Steele , H. S. J. V. D. Zant , A. Castellanos‐Gomez , 2D Mater. 2015, 2, 011002.

[393] G. Abellán , S. Wild , V. Lloret , N. Scheuschner , R. Gillen , U. Mundloch , J. Maultzsch , M. Varela , F. Hauke , A. Hirsch , J. Am. Chem. Soc. 2017, 139, 10432. 28675300 10.1021/jacs.7b04971 PMC5578363

[394] F. Xia , H. Wang , J. C. M. Hwang , A. H. C. Neto , L. i Yang , Nat. Rev. Phys. 2019, 1, 306.

[395] M. Pumera , Z. Sofer , Adv. Mater. 2017, 29, 1605299. 10.1002/adma.201605299 28185366

[396] S. Zhang , Z. Yan , Y. Li , Z. Chen , H. Zeng , Angew. Chem., Int. Ed. 2015, 54, 3112. 10.1002/anie.201411246 25564773

[397] Y. Zhang , T. Mori , L. Niu , J. Ye , Energy Environ. Sci. 2011, 4, 4517.

[398] J. Ran , T. Y. Ma , G. Gao , X.‐W. Du , S. Z. Qiao , Energy Environ. Sci. 2015, 8, 3708.

[399] F. Ghasemi , S. Mohajerzadeh , ACS Appl. Mater. Interfaces 2016, 8, 31179. 27792304 10.1021/acsami.6b07211

[400] R. Zhang , M. Hummelgård , V. Forsberg , H. Andersson , M. Engholm , T. Öhlund , M. Olsen , J. Örtegren , H. Olin , Sci. Rep. 2018, 8, 3296. 29459668 10.1038/s41598-018-21688-0 PMC5818540

[401] R. A. Wells , H. Johnson , C. R. Lhermitte , S. Kinge , K. Sivula , ACS Appl. Nano Mater. 2019, 2, 7705.

[402] J. A. Desai , N. Adhikari , A. B. Kaul , RSC Adv. 2019, 9, 25805. 10.1039/c9ra03644j PMC9070084 35530073

[403] T. Leng , K. Parvez , K. Pan , J. Ali , D. Mcmanus , K. S. Novoselov , C. Casiraghi , Z. Hu , 2D Mater. 2020, 7, 024004.

[404] Abid , P. Sehrawat , S. S. Islam , J. Appl. Phys. 2019, 125, 154303.

[405] P. Ramasamy , D. Kwak , D.‐H. Lim , H.‐S. Ra , J.‐S. Lee , J. Mater. Chem. C 2016, 4, 479.

[406] J. Li , J. Han , H. Li , X. Fan , K. Huang , Mater. Sci. Semicond. Process. 2020, 107, 104804.

[407] S. A. Bhakhar , N. F. Patel , C. K. Zankat , M. Tannarana , G. K. Solanki , K. D. Patel , V. M. Pathak , P. Pataniya , Mater. Sci. Semicond. Process. 2019, 98, 13.

[408] S. Ghosh , A. Winchester , B. Muchharla , M. Wasala , S. Feng , A. L. Elias , M. B. M. Krishna , T. Harada , C. Chin , K. Dani , S. Kar , M. Terrones , S. Talapatra , Sci. Rep. 2015, 5, 11272. 26175112 10.1038/srep11272 PMC4502394

[409] J. Lee , R. Hahnkee Kim , S. Yu , D. Babu Velusamy , H. Lee , C. Park , S. M. Cho , B. Jeong , H. Sol Kang , C. Park , 2D Mater. 2017, 4, 041002.

[410] R. Ahmad , R. Srivastava , S. Yadav , D. Singh , G. Gupta , S. Chand , S. Sapra , J. Phys. Chem. Lett. 2017, 8, 1729. 28350471 10.1021/acs.jpclett.7b00243

[411] Y. Wei , V.‐T. Tran , C. Zhao , H. Liu , J. Kong , H. Du , ACS Appl. Mater. Interfaces 2019, 11, 21445. 31185567 10.1021/acsami.9b01515

[412] M. Chhowalla , H. S. Shin , G. Eda , L.‐J. Li , K. P. Loh , H. Zhang , Nat. Chem. 2013, 5, 263. 23511414 10.1038/nchem.1589

[413] A. Kuc , N. Zibouche , T. Heine , Phys. Rev. B: Condens. Matter Mater. Phys. 2011, 83, 245213.

[414] P. Tonndorf , R. Schmidt , P. Böttger , X. Zhang , J. Börner , A. Liebig , M. Albrecht , C. Kloc , O. Gordan , D. R. T. Zahn , S. Michaelis De Vasconcellos , R. Bratschitsch , Opt. Express 2013, 21, 4908. 23482024 10.1364/OE.21.004908

[415] W. Zhao , Z. Ghorannevis , L. Chu , M. Toh , C. Kloc , P.‐H. Tan , G. Eda , ACS Nano 2013, 7, 791. 23256505 10.1021/nn305275h

[416] W.‐J. Ong , L.‐L. Tan , Y. H. Ng , S.‐T. Yong , S.‐P. Chai , Chem. Rev. 2016, 116, 7159. 27199146 10.1021/acs.chemrev.6b00075

[417] J. Wang , Z. Guan , J. Huang , Q. Li , J. Yang , J. Mater. Chem. A 2014, 2, 7960.

[418] V. K. Sangwan , M. C. Hersam , Annu. Rev. Phys. Chem. 2018, 69, 299. 29463170 10.1146/annurev-physchem-050317-021353

[419] F. Liu , H. Shimotani , H. Shang , T. Kanagasekaran , V. Zólyomi , N. Drummond , V. I. Fal'ko , K. Tanigaki , ACS Nano 2014, 8, 752. 24364508 10.1021/nn4054039

[420] D. A. Bandurin , A. V. Tyurnina , G. L. Yu , A. Mishchenko , V. Zólyomi , S. V. Morozov , R. K. Kumar , R. V. Gorbachev , Z. R. Kudrynskyi , S. Pezzini , Z. D. Kovalyuk , U. Zeitler , K. S. Novoselov , A. Patanè , L. Eaves , I. V. Grigorieva , V. I. Fal'ko , A. K. Geim , Y. Cao , Nat. Nanotechnol. 2017, 12, 223. 27870843 10.1038/nnano.2016.242

[421] G. W. Mudd , S. A. Svatek , T. Ren , A. Patanè , O. Makarovsky , L. Eaves , P. H. Beton , Z. D. Kovalyuk , G. V. Lashkarev , Z. R. Kudrynskyi , A. I. Dmitriev , Adv. Mater. 2013, 25, 5714. 23966225 10.1002/adma.201302616 PMC4065344

[422] Z. Q. Zheng , J. D. Yao , G. W. Yang , J. Mater. Chem. C 2016, 4, 8094.

[423] J. O. Island , S. I. Blanter , M. Buscema , H. S. J. Van Der Zant , A. Castellanos‐Gomez , Nano Lett. 2015, 15, 7853. 26540135 10.1021/acs.nanolett.5b02523

[424] K. Park , J. Korean Phys. Soc. 2018, 73, 817.

[425] H. Peng , W. Dang , J. Cao , Y. Chen , D. Wu , W. Zheng , H. Li , Z.‐X. Shen , Z. Liu , Nat. Chem. 2012, 4, 281. 22437712 10.1038/nchem.1277

[426] H. Zhang , C.‐X. Liu , X.‐L. Qi , X. Dai , Z. Fang , S.‐C. Zhang , Nat. Phys. 2009, 5, 438.

[427] M. Ghidiu , M. R. Lukatskaya , M.‐Q. Zhao , Y. Gogotsi , M. W. Barsoum , Nature 2015, 516, 78. 10.1038/nature13970 25470044

[428] E. Lee , A. Vahidmohammadi , B. C. Prorok , Y. S. Yoon , M. Beidaghi , D.‐J. Kim , ACS Appl. Mater. Interfaces 2017, 9, 37184. 28953355 10.1021/acsami.7b11055

[429] S. Lai , J. Jeon , S. K. Jang , J. Xu , Y. J. Choi , J.‐H. Park , E. Hwang , S. Lee , Nanoscale 2015, 7, 19390. 26535782 10.1039/c5nr06513e

[430] S. Ahn , T.‐H. Han , K. Maleski , J. Song , Y.‐H. Kim , M.‐H. Park , H. Zhou , S. Yoo , Y. Gogotsi , T.‐W. Lee , Adv. Mater. 2020, 32, 2000919. 10.1002/adma.202000919 32350958

[431] D. Pang , M. Alhabeb , X. Mu , Y. Dall'agnese , Y. Gogotsi , Y. Gao , Nano Lett. 2019, 19, 7443. 31536705 10.1021/acs.nanolett.9b03147

[432] M. Khazaei , M. Arai , T. Sasaki , A. Ranjbar , Y. Liang , S. Yunoki , Phys. Rev. B: Condens. Matter Mater. Phys. 2015, 92, 075411.

[433] O. Mashtalir , K. M. Cook , V. N. Mochalin , M. Crowe , M. W. Barsoum , Y. Gogotsi , J. Mater. Chem. A 2014, 2, 14334.

[434] M. Naguib , O. Mashtalir , M. R. Lukatskaya , B. Dyatkin , C. Zhang , V. Presser , Y. Gogotsi , M. W. Barsoum , Chem. Commun. 2014, 50, 7420. 10.1039/c4cc01646g 24821374

[435] S. Hong , G. Zou , H. Kim , D. Huang , P. Wang , H. N. Alshareef , ACS Nano 2020, 14, 9042. 32538614 10.1021/acsnano.0c04099 PMC7467806

[436] L. Sun , Y. Qi , C.‐J. Jia , Z. Jin , W. Fan , Nanoscale 2014, 6, 2649. 24442108 10.1039/c3nr06104c

[437] C. C. Coleman , H. Goldwhite , W. Tikkanen , Chem. Mater. 1998, 10, 2794.

[438] N. Preda , L. Mihut , M. Baibarac , I. Baltog , Acta Phys. Pol. A 2009, 116, 81.

[439] T. Sasaki , M. Watanabe , H. Hashizume , H. Yamada , H. Nakazawa , J. Am. Chem. Soc. 1996, 118, 8329.

[440] A. Takagaki , D. Lu , J. N. Kondo , M. Hara , S. Hayashi , K. Domen , Chem. Mater. 2005, 17, 2487.

[441] N. Nhlapo , T. Motumi , E. Landman , S. M. C. Verryn , W. W. Focke , J. Mater. Sci. 2008, 43, 1033.

[442] Q. Wu , A. O. Sjåstad , Ø. B. Vistad , K. D. Knudsen , J. Roots , J. S. Pedersen , P. Norby , J. Mater. Chem. 2007, 17, 965.

[443] T. Alzoubi , H. Qutaish , E. Al‐Shawwa , S. Hamzawy , Opt. Mater. (Amst). 2018, 77, 226.

[444] D. O'suilleabhain , V. Vega‐Mayoral , A. G. Kelly , A. Harvey , J. N. Coleman , ACS Appl. Mater. Interfaces 2019, 11, 8545. 30698947 10.1021/acsami.8b21416

[445] A. G. Kelly , C. Murphy , V. Vega‐Mayoral , A. Harvey , A. S. Esmaeily , T. Hallam , D. Mccloskey , J. N. Coleman , 2D Mater. 2017, 4, 041006.

[446] A. Armin , R. D. Jansen‐Van Vuuren , N. Kopidakis , P. L. Burn , P. Meredith , Nat. Commun. 2015, 6, 6343. 25721323 10.1038/ncomms7343

[447] M. B. Johnston , Nat. Photonics 2015, 9, 634.

[448] Q. Lin , A. Armin , P. L. Burn , P. Meredith , Nat. Photonics 2015, 9, 687.

[449] Y. Fang , Q. Dong , Y. Shao , Y. Yuan , J. Huang , Nat. Photonics 2015, 9, 679.

